# Glucocorticoid receptors in oligodendrocyte precursor cells regulate hippocampal network plasticity and stress-induced behavior in mice

**DOI:** 10.1073/pnas.2614867123

**Published:** 2026-07-20

**Authors:** Lorenzo Mattioni, Giulia Poggi, Celine Gallagher, Katrin Becker, Linh Le, Maja Papic, Jasmin Engbers, Maija-Kreetta Koskinen, Ali Abdollahzadeh, David P. Herzog, Leonardo Nardi, Andrea Conrad, Sarah Winterberg, Christa Merte-Grebe, Liana Melo-Thomas, Hyonseung Lee, Hans Schwarzbach, Jennifer Klüpfel, Ralf Kinscherf, Jan Engelmann, Beat Lutz, Ari Waisman, Iiris Hovatta, Thomas Mittmann, Michael J. Schmeisser, Marianne B. Müller, Giulia Treccani

**Affiliations:** ^a^Institute of Anatomy, University Medical Center of the Johannes Gutenberg-University, Mainz 55128, Germany; ^b^Focus Program Translational Neurosciences, University Medical Center of the Johannes Gutenberg-University, Mainz 55128, Germany; ^c^Department of Psychiatry and Psychotherapy, University Medical Center of the Johannes Gutenberg-University, Mainz 55128, Germany; ^d^Institute of Physiology, University Medical Center of the Johannes Gutenberg-University, Mainz, Germany; ^e^ Leibniz Institute for Resilience Research, Mainz 55128, Germany; ^f^Institute for Molecular Medicine, University Medical Center of the Johannes Gutenberg-University, Mainz 55128, Germany; ^g^ SleepWell Research Program, Faculty of Medicine, University of Helsinki, Helsinki 00014, Finland; ^h^ Department of Psychology, Faculty of Medicine, University of Helsinki, Helsinki 00014, Finland; ^i^ A. I. Virtanen Institute for Molecular Sciences, University of Eastern Finland, Kuopio 70211, Finland; ^j^ Institute of Physiological Chemistry, University Medical Center of the Johannes Gutenberg University, Mainz 55128, Germany; ^k^ Department of Systemic Neuroscience, Institute of Anatomy and Cell Biology, Philipps University, Marburg 35032, Germany; ^l^Department of Medical Cell Biology, Institute for Anatomy and Cell Biology, Medical Faculty, Philipps University Marburg, Marburg 35032, Germany

**Keywords:** NG2-glia, OPCs, glucocorticoid receptor, hippocampus, learning and memory

## Abstract

Glucocorticoid receptors (GRs) are key mediators of how the stress hormone glucocorticoids (GCs) shape postnatal brain development and adaptive plasticity. Because GC signaling is critical during this period, postnatal GC concentrations are tightly regulated in the brain, whereas excessive levels of circulating GCs can disrupt developmental trajectories and increase the risk of psychiatric disorders later in life. GR function influences multiple neural cell types, but its cell-specific roles, particularly early in development, remain poorly understood. Oligodendrocyte precursor cells (OPCs), which generate myelinating oligodendrocytes and actively modulate neuronal networks, express GRs and can therefore respond to fluctuations in GC levels. Although excessive GC exposure during early life adversity has been linked to changes in OPC development, the physiological role of GR signaling specifically within OPCs remains unclear. To address this, we conditionally deleted GRs in postnatal OPCs in mice to investigate the role of physiological GC signaling in OPC proliferation and maturation, as well as in neuronal network activity and behavior. This deletion resulted in reduced oligodendrocyte and myelinated axon density in the hippocampus, sex-specific alterations in hippocampal activity and long-term potentiation following acute challenge, and impairments in memory formation in adulthood. Our findings reveal an OPC-specific role for GRs and suggest that physiological GR activity in the oligodendrocyte lineage contributes to normal hippocampal plasticity, learning, and memory.

## Significance

Glucocorticoids shape postnatal brain development and adaptive plasticity through glucocorticoid receptors (GRs), yet their actions in non-neuronal cells remain poorly understood. Here, we identify oligodendrocyte precursor cells (OPCs) as key targets of GR signaling during early life. Deleting GR in postnatal OPCs reduced oligodendrocyte and myelinated axon density in the hippocampus, altered hippocampal network plasticity in response to glucocorticoid challenge, and impaired memory formation in adulthood. Notably, several functional consequences were sex specific, including altered hippocampal plasticity and impaired performance under aversive learning conditions. These findings reveal an OPC-specific role for GRs and suggest that physiological GR activity in the oligodendrocyte lineage contributes to normal hippocampal plasticity, learning, and memory.

The early postnatal period is a critical developmental stage, when exposure to hormones influences brain maturation (1). Glucocorticoids (GCs), the main hormones released in response to stress, are tightly regulated during this time of development that is defined as the stress-hyporesponsive period (SHRP) and is characterized by reduced secretion of GCs following mild stress conditions (2). Although exposure to negative stress in early life can override the SHRP and expose the brain to harmful surges in GCs, endogenous GCs are also present postnatally and play a beneficial role in brain development (3). Nevertheless, the specific cellular effects of GCs on postnatal brain maturation remain poorly understood.

Importantly, different cell populations of the brain are endowed with glucocorticoid receptors (GRs), making them sensitive to physiological fluctuations, but also to stress-induced increase of GCs (4). One glial cell population that has been identified both clinically and preclinically as an emerging player in stress-related psychiatric disorders is oligodendrocyte precursor cells (OPCs) (5–9). OPCs express several markers, including the chondroitin sulfate proteoglycan 4 (CSPG4) or neuron–glial antigen 2 (NG2), and are therefore also referred to as NG2-glia (10, 11). They are homogeneously distributed throughout the CNS, both during development and adulthood, and they are highly responsive to external stimuli, including neuronal activity (12). Through their canonical pathway, OPCs can give rise to myelinating oligodendrocytes. However, a large proportion of OPCs remain undifferentiated and survey the surrounding extracellular environment (13). These resident OPCs are thought to play a critical role in modulating and fine-tuning neuronal networks through noncanonical (and myelination-independent) pathways (14–18) and are indeed integrated into the neuronal network via synapses with neighboring neurons (19–21). Mice exposed to maternal separation [a model of early life adversity (ELA)] have an increased number of mature oligodendrocytes at postnatal day (PD)15 and a reduction in OPCs as adults (22). In postmortem studies on individuals exposed to ELA, increased numbers of mature myelinating oligodendrocytes (OLs) and decreased numbers of OPCs were found (23). Taken together, these findings suggest enhanced maturation of OPCs as a result of their early exposure to excess GCs. Recently, we have shown that ELA not only alters the density of OPCs or OLs, but has a direct effect on the OPC gene expression patterns and their electrical properties and that these effects are largely GR-dependent (24).

OPCs remain responsive to stress signals throughout the adult lifespan (25–27) and express GRs in both gray and white matter regions (28–30), indicating that GCs may play a physiological role in the regulation of OPC function and differentiation (31). Although there is accumulating evidence for the influence of GCs on OPC proliferation and maturation, the specific contribution of constitutive GR signaling in OPCs to neuronal network function and behavior remains unclear. Therefore, the aim of this study was to determine how physiological developmental GR expression in OPCs regulates their proliferation and differentiation, and whether GRs in OPCs contribute to modulating hippocampal network activity and fine-tuning of behavioral phenotypes, including those behaviors induced by stress in adulthood. We conditionally deleted GRs in OPCs during the first postnatal week, a developmental window characterized by peak OPC proliferation (32), and examined the long-term consequences on oligodendrocyte dynamics, hippocampal network excitability, and adult behavior. Our findings reveal that postnatal GR signaling in OPCs is required for OPC maturation and axon myelination, fine-tuning of neuronal network potentiation in responses to challenge, and the establishment of adult memory.

## Results

### Conditional Deletion of GR in OPCs Impairs Lineage Maturation and Density of Myelinated Axons Without Affecting Proliferation and Morphology.

**Fig. 1.**
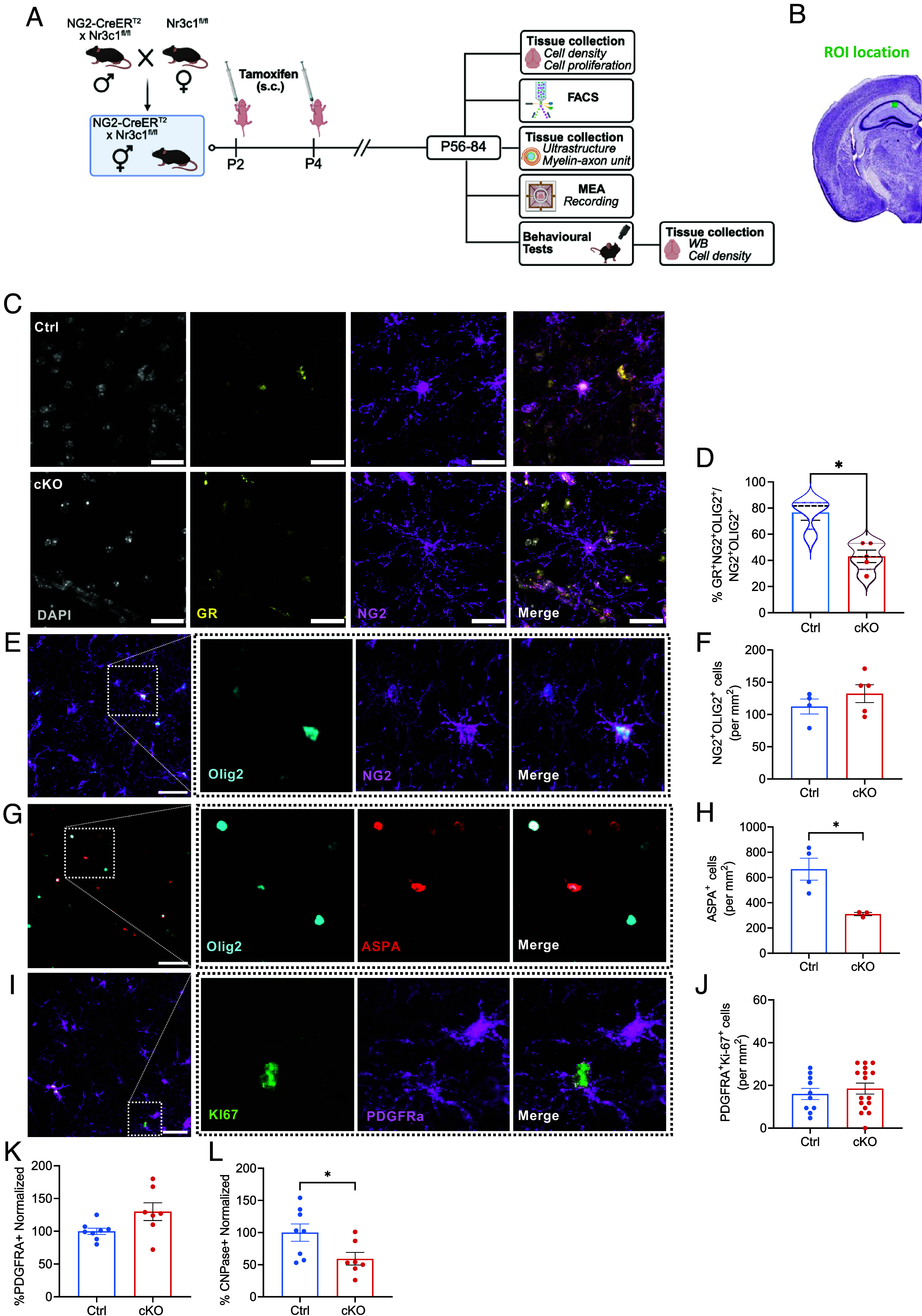
OPCs-specific GR cKO alters oligodendrocyte density but does not induce changes in OPC density and proliferation. (*A*) Breeding strategy and experimental timeline for tamoxifen injections, behavioral studies, MEA recordings, and immunohistochemical analysis. (*B*) Region of interest (ROI, Cornu ammonis 1 (CA1), Bregma: −1.79 to −2.53 mm, image adapted from Paxinos and Franklin mouse brain atlas). (*C*–*J*) Immunofluorescence in the hippocampus. (*C*) Representative confocal images of the coexpression of GR (yellow) and NG2 (magenta) in the hippocampus of Ctrl and cKO mice. (Scale bar, 20 µm.) (*D*) Percentage of GR + NG2+ cells (Mann–Whitney test: U = 0, *P* = 0.0159; n = 4 Ctrl and n = 5 cKO mice). Representative confocal images with digital magnification of the boxed area and related quantification for (*E* and *F*) NG2 (magenta) and (*G* and *H*) ASPA (red), each costained with Olig2 (cyan). (Scale bar, 20 µm.) (*F*) Density of NG2+ Olig2+ cells (unpaired *t* test: *P* = 0.3202; n = 4 Ctrl and n = 5 cKO mice). (*H*) Density of ASPA+ cells (unpaired *t* test with Welch’s correction: t(3.129) = 4.038, *P* = 0.0252; n = 4 Ctrl and n = 5 cKO mice). (*I*) Representative confocal images with digital magnification of the boxed area and related quantification for PDGRFA+ (magenta) and Ki-67+ (green) cells. (Scale bar, 20 µm.) (*J*) Density of PDGRFA + Ki67+ cells (unpaired *t* test: *P* = 0.5153; n = 10 Ctrl and n = 16 cKO mice). Brightness and contrast of the micrographs have been adjusted for display purposes. (*K* and *L*) Flow cytometry in hippocampal tissue. (*K*) Percentage of PDGRFA+ cells in the CD1145−CD11b− population (unpaired *t* test with Welch’s correction: *P* = 0.073; n = 8 Ctrl and n = 7 cKO mice). (*L*) Percentage of CNPase+ cells in the CD1145−CD11b− population (unpaired *t* test: t(13) = 2.384, *P* = 0.0331; n = 8 Ctrl and n = 7 cKO mice). Data are expressed as the mean ± SEM (with graph *D* also including an overlaid violin plot for median and data range) and each dot represents one animal. *P < 0.05.

To investigate the role of an early conditional knockout (cKO) of GR on OPC differentiation, morphology, as well as behavioral phenotypes in adult mice, we generated an OPC-specific GR cKO mouse strain. We obtained the cKO mice by crossing the Ng2CreER^T2^ mice with Nr3c1^fl/fl^ mice and inducing recombination by tamoxifen injection at PDs 2 and 4 (Fig. 1*A*; *Materials and Methods*). As previously reported (28, 30), we confirmed by fluorescence-activated cell sorting (FACS) analysis that GRs are expressed by the hippocampal and cortical OPCs and mature oligodendrocytes (OLs), with greater concentration in OPCs (*SI Appendix*, Fig. S1 *A* and *B*). We then assessed the efficiency of GR deletion in OPCs by performing immunofluorescence (IF) staining in the dorsal hippocampus (Fig. 1*B*) and the dorsally located somatosensory cortex of the cKO and control littermates. Immunostaining revealed a decrease in the percentage of GR+ NG2+ Olig2+ OPCs in cKO mice in the hippocampus (Fig. 1 *C* and *D*) and cortex (*SI Appendix,* Fig. S2*B*), where there were no sex differences (*SI Appendix,* Fig. S3 *A* and *B*). To confirm the specificity of GR deletion in OPCs, we performed costaining of GR with several cell-specific markers, including Glial fibrillary acidic protein (GFAP) for astrocytes (*SI Appendix*, Fig. S4 *A*–*C*), ionized calcium binding adaptor molecule 1 (Iba1) for microglia (*SI Appendix*, Fig. S4 *D*–*F*), NeuN for neurons (*SI Appendix*, Fig. S4 *G*–*I*), and platelet-derived growth factor receptor beta (PDGFRB) for pericytes (*SI Appendix*, Fig. S4 *J*–*L*). We did not observe a reduction of GRs in any of these cell populations in cKO mice, either in the hippocampus or in the cortex (*SI Appendix*, Fig. S4).

We then tested for effects of cKO of GR on hippocampal density of OPCs and OLs, stained with NG2 and Aspartoacylase (ASPA, marker for mature myelinating OLs), respectively. While we found no change in the density of OPCs (Fig. 1 *E* and *F*), there was a decrease in the density of mature OLs (Fig. 1 *G* and *H*) in the hippocampus of cKO mice. These data were confirmed by FACS analysis, which showed no difference in the number of PDGFRA+ OPCs and a decrease in CNPase+ OLs in the CD45-CD11b- global populations (Fig. 1 *K* and *L*).

Given an imbalance in OPC and OL ratio may be a consequence of an alteration in OPC proliferation (33), we performed IF staining for Ki-67, a marker for cell proliferation, and PDGFRA, a marker for OPCs, and quantified the overall density of OPCs (PDGFRA+ cells), proliferative cells (Ki-67+ cells), and proliferative OPCs (PDGFRA+ Ki-67+ cells). The density of proliferative OPCs was comparable between cKO and littermate controls (Fig. 1 *I* and *J*), in contrast, a slightly different pattern was observed in the cortex. Indeed, while a reduction of OPC density was observed when employing the NG2 marker (*SI Appendix*, Fig. S2*C*), no changes were observed in OL density (*SI Appendix*, Fig. S2*D*), OPC proliferation (*SI Appendix*, Fig. S2*E*), or OPC density, when using PDGFRA as a marker (*SI Appendix*, Fig. S2*G*). The absence of difference in the density of OPCs and OLs in the cortex was further validated by FACS analysis (*SI Appendix*, Fig. S2 *H* and *I*).

A fundamental feature of OPCs is their morphological complexity that allows for continuous and efficient surveillance of the surrounding environment and has been shown to diminish when OPCs undergo apoptosis or differentiation into mature oligodendrocytes (13, 26). Critically, Sholl analysis showed that GR KO in OPCs did not influence filament cell length (*SI Appendix*, Fig. S5 *A* and *B*) or complexity of the arborization (*SI Appendix*, Fig. S5*C*). Qualitative analysis of single OPC complexity did not suggest heterogeneity within the cell population (*SI Appendix,* Fig. S5 *D* and *E*). This indicates that loss of GR is unlikely to severely alter OPCs surveillance capabilities and, in line with the density readouts, does not lead to cell atrophy, such as in response to stressors prior to apoptosis (26).

**Fig. 2.**
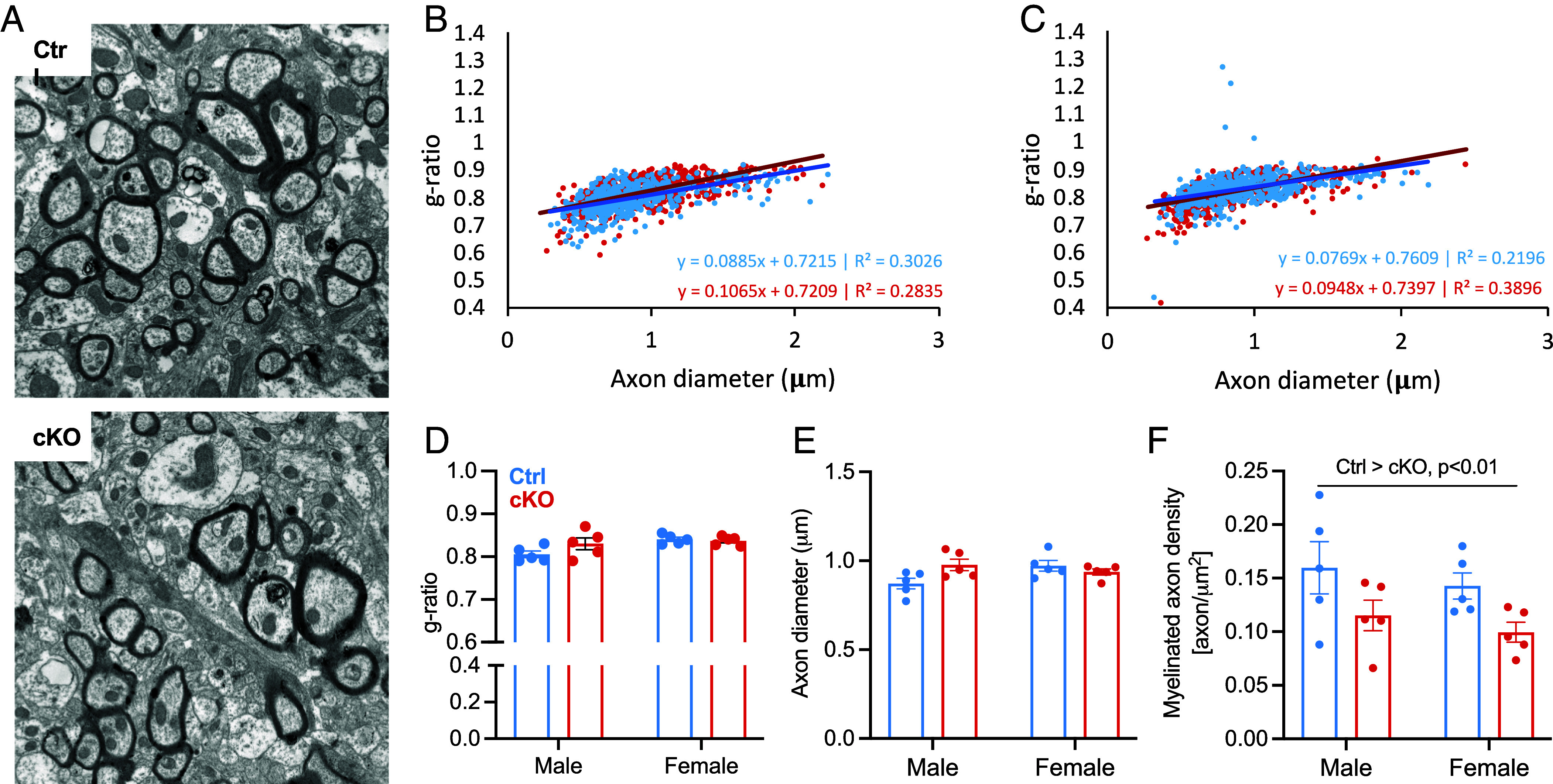
OPC-specific GR cKO reduces the density of myelinated axons in the hippocampus of male and female mice. (*A*) Representative transmission electron microscopy micrograph from Ctrl vs. cKO male. (Scale bar, 1 µm.) (*B*) Relation between g-ratio and axonal diameter in Ctr and cKO male mice. (*C*) Relation between g-ratio and axonal diameter in Ctr and cKO female mice. (*D*) g-ratio (two-way ANOVA, genotype: F(1, 16) = 1.559, *P* = 0.2298; sex: F(1, 16) = 5.878, *P* = 0.0275; genotype × sex: F(1, 16) = 2.760, *P* = 0.1161). (*E*) Axon diameter (two-way ANOVA, genotype: F(1, 16) = 1.520, *P* = 0.2354; sex: F(1, 16) = 1.131, *P* = 0.3034; genotype × sex: F(1, 16) = 6.245, *P* = 0.0237. Tukey’s multiple comparisons test, n.s.). (*F*) Density of myelinated axons (two-way ANOVA, genotype: F(1, 16) = 7.468, *P* = 0.0147; sex: F (1, 16) = 1.028, *P* = 0.3258; genotype × sex: F(1, 16) = 0.001840, *P* = 0.9663). Data are expressed as the mean ± SEM. *P < 0.05.

To assess whether the reduction in mature oligodendrocyte density was reflected in an overall difference in myelin content at ultrastructural level, we performed electron microscopy on hippocampal specimens (Fig. 2*A*). The relation between g-ratio and axon diameter was estimated in male (Fig. 2*B*) and female mice (Fig. 2*C*). While there were small sex-differences in the g-ratio (slightly higher in females) (Fig. 2*D*), but no genotype or sex differences in axon diameter (Fig. 2*E*), we found a decrease in the density of myelinated axons in both sexes of cKO mice (Fig. 2*F*). When analyzing myelin-related proteins, we did not detect any differences in MBP (all isoforms analyzed together), PLP (34), CNP, or ASPA analyzed by western blot (WB), in the hippocampus and cortex of cKO and control mice (*SI Appendix*, Figs. S6 and S7). We also performed 3D reconstruction and morphological analysis of the paranodes, nodes of Ranvier, and length of the internodes in control vs. cKO mice and found no differences in sex and genotypes between the groups (*SI Appendix*, Fig. S8).

Overall, these data demonstrate that loss of GR in OPCs in early life reduces the density of oligodendrocytes and of myelinated axons in the hippocampus of adult mice, without affecting myelin ultrastructure. The absence of changes in myelin protein content, axon-myelin units, and overall internode architecture further indicates that GR loss selectively alters specific features of the OL lineage and myelination within the hippocampus.

### Early Conditional Deletion of GR in OPCs Alters the Excitability of the Neuronal Network in Response to Acute Challenge Later in Life.

**Fig. 3.**
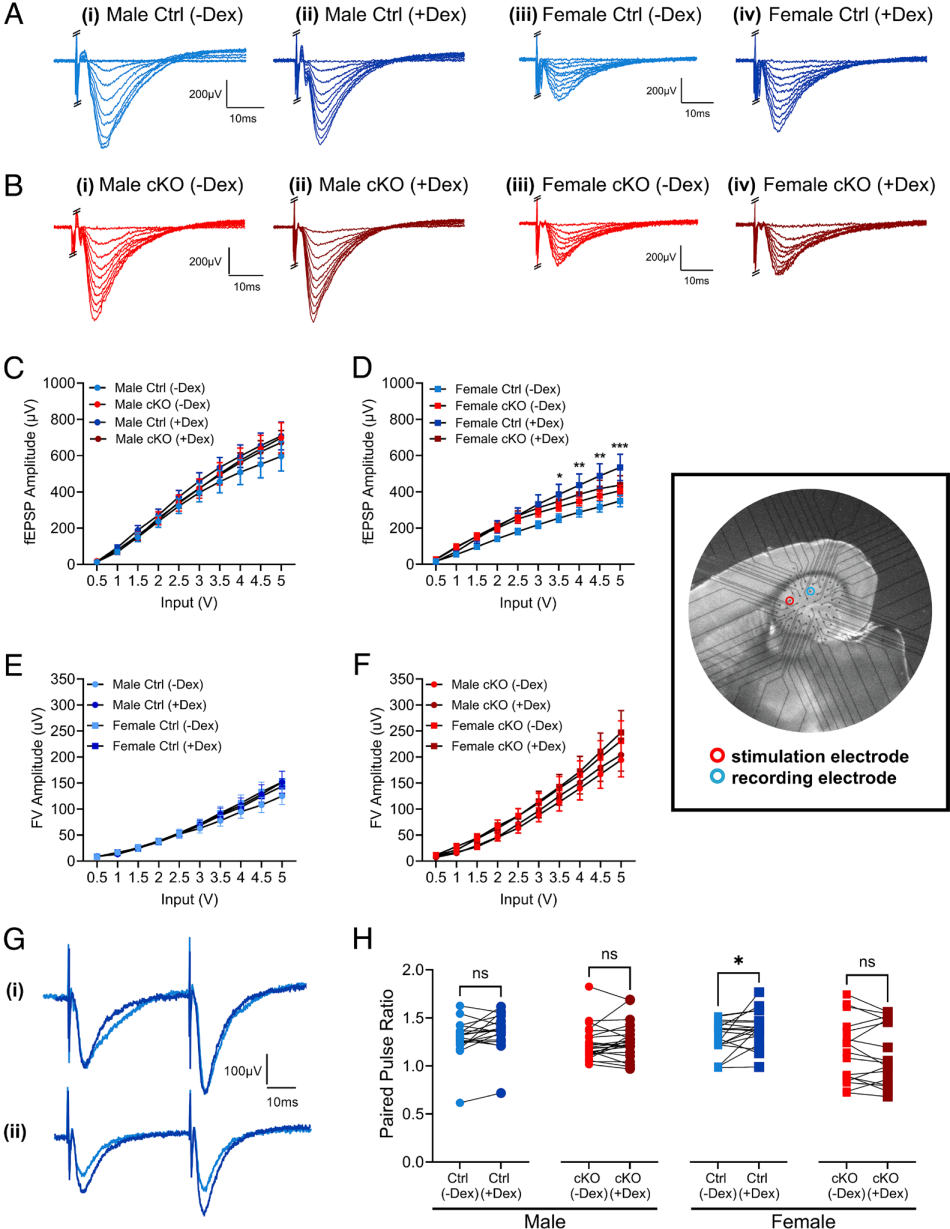
OPCs-specific GR affects hippocampal network excitability under acute stress conditions selectively in adult female mice. (*A*) Representative overlaid voltage traces of the evoked I/O responses from male wild-type mice before (*i*) and after (*ii*) dexamethasone application, and from female wild-type mice before (*iii*) and after (*iv*) dexamethasone application. (*B*) Representative overlaid voltage traces of the evoked I/O responses from male cKO mice before (*i*) and after (*ii*) dexamethasone application, and from female cKO mice before (*iii*) and after (*iv*) dexamethasone application. (*C*) Mean evoked fEPSP amplitudes at different stimulus intensities in the CA1 region of adult male wild-type and cKO mice before (light blue/red, respectively) and 30 min after (dark blue/red, respectively) bath application of dexamethasone [Three-factor statistical model, male. Three-way RM ANOVA: input, F(9.000, 342.0) = 166.0, *P* < 0.0001; n = 18 WT (−Dex), n = 22 cKO (−Dex), n = 18 WT (+Dex), n = 22 KO (+Dex)]. (*D*) Same recordings as in (*A*), but here performed in adult female wild-type and cKO mice (*Three-factor statistical model, female*. Mixed-effects model: Input × Genotype × Dex, F(1.162, 35.88) = 3.622, *P* = 0.0539; Statistical analysis per genotype – Female, Ctrl. Mixed-effects model: Input × Dex, F(1.128, 16.80) = 5.702. Paired *t* test as post hoc comparison: input 3.5: (−Dex) < (+Dex), *P* = 0.0406; input 4: (−Dex) < (+Dex), *P* = 0.0329; input 4.5: (−Dex) < (+Dex), *P* = 0.0249; input 5: (−Dex) < (+Dex), *P* = 0.0238; n = 17 (−Dex), n = 16 (+Dex). *Statistical analysis per genotype – Female, cKO*. Two-way RM ANOVA, Input, F(1.402, 22.43) = 90.15, *P* < 0.0001; Dex and interaction, *P* = 0.2581; n = 17 (−Dex), n = 17 (+Dex)). (*E*) Mean fiber volley (FV) amplitudes recorded at different stimulus intensities in the CA1 region of adult male and female wild-type mice before (light blue circles/squares, respectively) and 30 min after (dark blue circles/squares, respectively) bath application of dexamethasone (three-way RM ANOVA, input F(9, 279) = 103.3, *P* < 0.0001; drug, sex *P* > 0.05). (*F*) Same recordings as in (*E*), but here performed in cKO animals (three-way RM ANOVA, input F(9, 288) = 76.00, *P* < 0.0001; drug, sex *P* > 0.05). (*G*) (*i*) Representative voltage traces of male Ctrl paired-pulse stimulation responses before (light blue) and after (dark blue) dexamethasone application. (*ii*) Representative voltage traces of female Ctrl paired-pulse stimulation responses before (light blue) and after (dark blue) dexamethasone application. (*H*) Summary plots of the paired-pulse ratio (PPR) of fEPSP-amplitudes recorded in male and female Ctrl- and cKO-mice, before and after 30-min dexamethasone application (Female, Ctrl. Paired *t* test: t(18) = 2.247, *P* = 0.0374; n = 19 (−Dex), n = 19 (+Dex). Female, cKO. Wilcoxon matched-pairs signed rank test: W = −31.00, *P* = 0.4874; n = 17 (−Dex), n = 17 (+Dex)). The *Inset* shows a representative acute hippocampal brain slice section on the MEA chip positioned with the stimulating electrode below the Schaffer Collaterals in area CA3 and the recording electrode below area CA1. Data are expressed as the mean ± SEM. *P < 0.05.

We tested whether the deletion of GR in OPCs impacts neuronal network functions, using electrophysiological multielectrode array (MEA) recordings in acute hippocampal slices under control conditions and in the presence of the potent GR agonist dexamethasone (35). This experimental condition mimics in vitro the activation of the hypothalamic–pituitary–adrenal (HPA) axis and, thus, the GR-related pathway. Horizontally sectioned brain slices were placed on the MEA chip to allow measurement of extracellular field excitatory postsynaptic potentials (fEPSPs) along the Schaffer Collateral CA3 to CA1 pathway (Fig. 3, *Inset*; for details see *Materials and Methods*). Electrically evoked fEPSPs were recorded in the CA1 region, with gradually increasing signal amplitudes plotted for male and female adult mice (Fig. 3 *A* and *B*). While there were no effects of dexamethasone treatment on the evoked mean fEPSP amplitudes in control and cKO male mice (Fig. 3*C*), increases were observed in control, but not cKO females for stimulation inputs ranging from 3.5 to 5 V (Fig. 3*D*). The missing increase of the fEPSP amplitude in female NG2-specific GR cKO mice implies that this receptor in OPCs mediates acute neuronal stress responses in females. In addition, the dexamethasone-induced increase of fEPSP amplitudes in female control mice indicated, at least in part, a sex-specific change of the postsynaptic function during acute stress.

Importantly, we cannot exclude that the sex and/or GR cKO affected the axonal excitability of the presynaptic Schaffer collaterals, so we further analyzed the FV component in our recorded fEPSP signals (Fig. 3 *E* and *F*). Here, we failed to detect any significant sex-specific changes in the mean amplitudes of the FV-component before and in presence of dexamethasone treatment (Fig. 3 *E* and *F*). This indicates that axonal excitability does not mediate the observed increase of fEPSP-amplitudes in female mice. Still, slices from GR cKO mice generally reached larger mean FV-amplitudes, linking to an additional contribution of the genotype. This was also visible when plotting the mean fEPSP amplitudes against the FV signal (*SI Appendix*, Fig. S9). When we further inspected the voltage traces of evoked fEPSPs before and after 30-min application of dexamethasone, we observed a sex-specific dichotomy that was evident prior to application of dexamethasone, given the higher evoked hippocampal excitability at baseline in both male wild-type and cKO animals as compared to female mice (*SI Appendix*, Fig. S10 *A* and *B*). We also performed a paired-pulse stimulation protocol to investigate whether dexamethasone application, mimicking acute stress exposure, induces changes in short-term synaptic plasticity at the Schaffer collateral pathway (Fig. 3*G*). Dexamethasone application caused a larger increase in the PPR only in the female control mice, whereas no effects were recorded in male control mice, cKO mice, and in female cKO mice (Fig. 3*H*). These results reflect those from the evoked fEPSP experiments above (Fig. 3 *C* and *D*). Representative traces of the paired-pulse stimulation responses, with a 50 ms interstimulus interval, show a lack of change in amplitude of either the first or second paired-pulse stimulation responses following application of dexamethasone for male controls (Fig. 3 *G, i*). In contrast, an increase in the amplitude of both the first and second paired-pulse stimulation responses following dexamethasone application was observed in female controls, with the second stimulation undergoing a relatively larger increase (Fig. 3 *G, ii*). The changes in paired-pulse stimulation responses indicated an altered synaptic facilitation, in which the probability of neurotransmitter release by the second stimulation remains high (Fig. 3*H*). We also found an overall slight genotype difference in PPR prior to dexamethasone application (*SI Appendix,* Fig. S10*C*, see *SI Appendix,* Table S1 for the detailed Statistics). We found no changes in the rate of spontaneous network activity in the hippocampal slices of either sex or genotype after dexamethasone application (*SI Appendix*, Fig. S10*D*). The neuronal spontaneous activity in these MEA recordings was verified by application of Tetrodotoxin (TTX), which blocked all signs of spontaneous activity (*SI Appendix*, Fig. S11). A sex-specific dichotomy was again observed in the mean spontaneous spike frequency before dexamethasone was applied, with female mice tending to have a higher mean spike frequency than male mice (*SI Appendix*, Fig. S10*E*).

In summary, the electrophysiological experiments suggest that acute stress primarily affects the hippocampal network in female control mice, divulging a fundamental sex-specific function of the NG2-cell-specific GR in the hippocampal Schaffer collateral pathway.

### Altered Long-term Potentiation in Acutely Stressed Female cKO Mice.

**Fig. 4.**
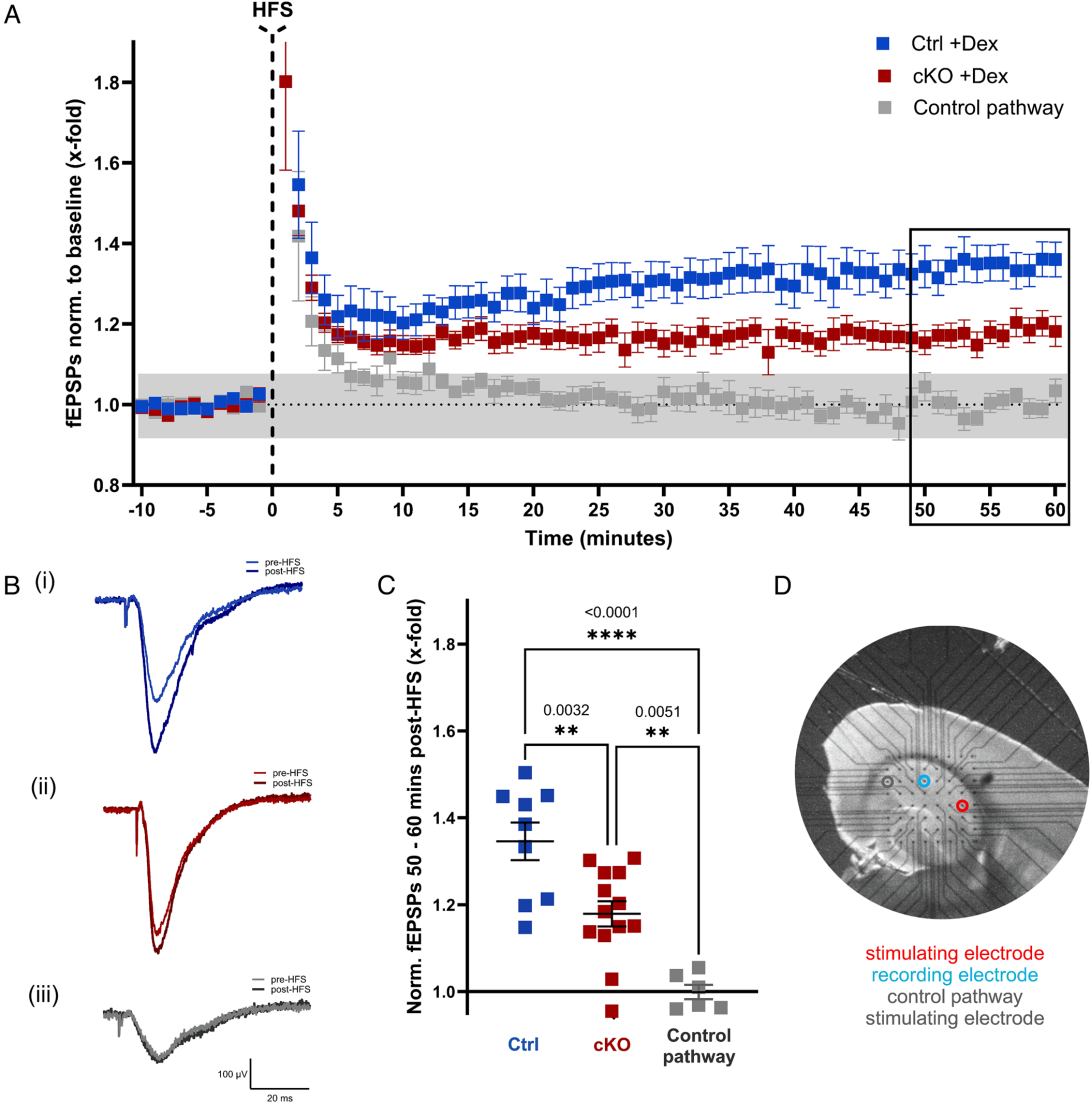
Female NG2-cKO mice show an impaired hippocampal LTP compared to female control mice under dexamethasone treatment. (*A*) LTP was induced at the Schaffer collaterals using a 100 Hz high-frequency stimulation (HFS) protocol and recorded in area CA1 using a microelectrode array (MEA) in female control and female NG2 cKO mice. Blue points represent the control female mice, red points represent the NG2 cKO female mice, and the gray dots represent the recordings taken from the independent control pathway. The graph shows mean ± SEM of the relative strength of potentiation of fEPSPs. Female control n = 9 slices from 3 mice, female cKO n = 13 slices from 5 mice, independent control pathway recordings n = 6 slices from 3 mice. (*B*) Representative fEPSP traces from both female control (blue) and female cKO (red) genotypes along with traces from the independent control pathway (gray). The darker of each color shows fEPSPs before the 100 Hz stimulation, the lighter of each color shows fEPSPs 60 min after the stimulation. (*C*) The relative strength of potentiation of the fEPSPs recorded at 50 to 60 min after the 100 Hz stimulation shows that both female genotypes had an LTP that was significantly greater than the control pathway (one-way ANOVA, F(2,25) = 19, *P* < 0.0001. Tukey’s multiple comparison test: female control > control pathway, *P* < 0.0001, female cKO > control pathway, *P* = 0.0051). However, an impairment of LTP in the female cKO genotype compared to the female control genotype can be seen from their significantly lower fEPSP potentiation post-HFS (one-way ANOVA, F(2,25) = 19, *P* < 0.0001. Tukey’s multiple comparison test: female Ctrl vs. female cKO, *P* = 0.0032). (*D*) Representative hippocampal section with an example of the location of stimulating electrode (red), recording electrode (blue), and stimulating electrode of the independent control pathway (gray) along the Schaffer Collaterals. Statistical analysis using one-way ANOVA with post hoc Tukey’s multiple comparisons test. The graph shows mean ± SEM.

As the electrophysiological experiments indicated a relevance of the NG2-GR for acute stress responses in female mice, female wild-type and cKO mice were then further tested for alterations in long-term potentiation (LTP) (Fig. 4*A*). We found that both wild-type and cKO mouse lines were capable of LTP induction and maintenance in the presence of bath-applied dexamethasone that mimics an acute in vivo stressor in our in vitro slice preparation (Fig. 4*A*). However, LTP in cKO slices exposed to dexamethasone was lower than in wild-type slices (Fig. 4 *B* and *C*). Thus, the cKO of GR in NG2 cells impairs hippocampal LTP along the Schaffer Collateral pathway, from the CA3 to the CA1 region, where reduced LTP may indicate impairments in behavioral tasks assessing learning and memory in female cKO mice under acute stress.

### Postnatal Deletion of GR in OPCs Induces Impairment in Memory and Aversive Learning Tasks but Does Not Alter Anxiety-relevant Behavior or Sociability in Adulthood.

**Fig. 5.**
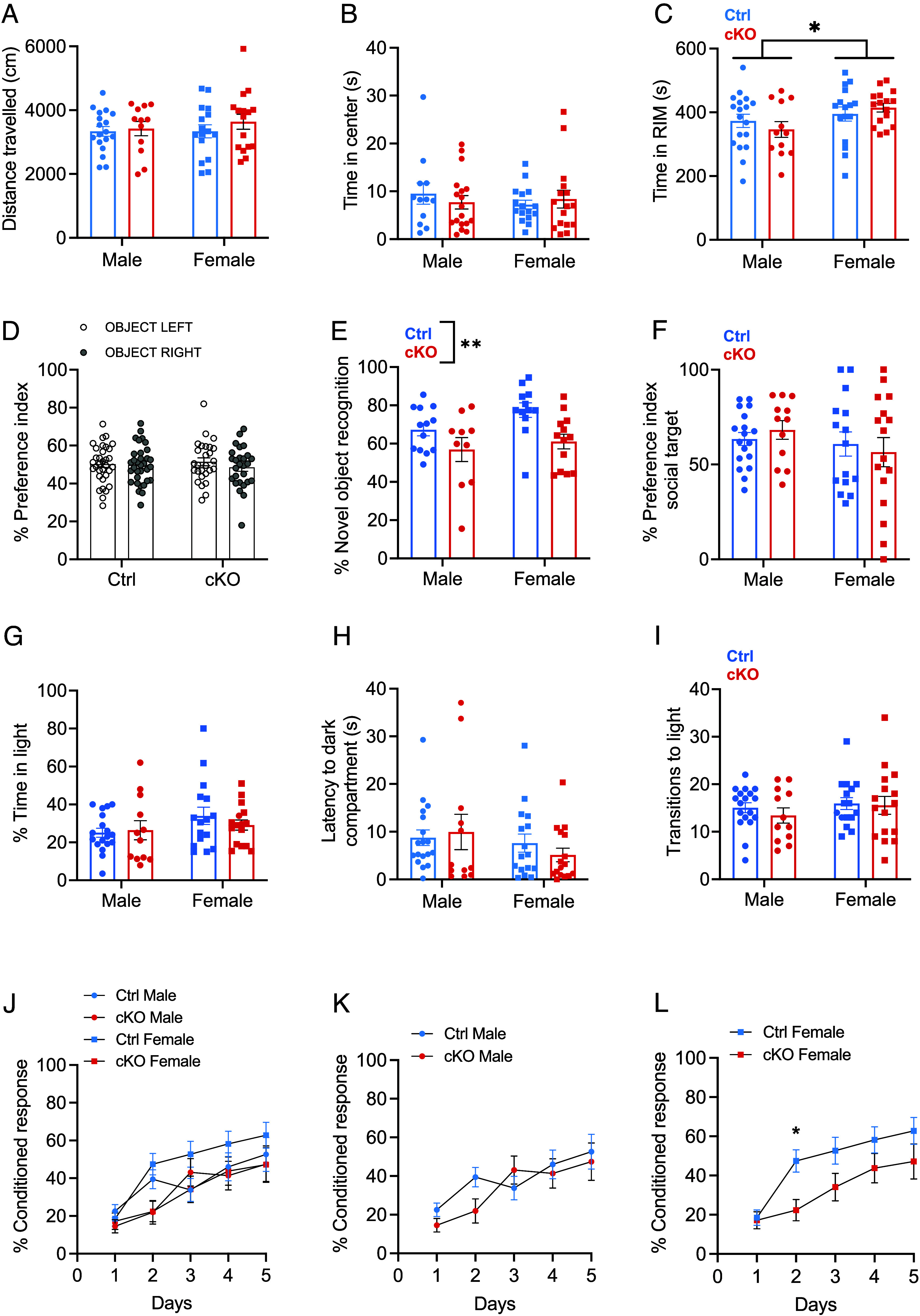
GR deletion in OPCs induces impairment in memory and aversive learning tasks. (*A*–*C*) Open field test. (*A*) Distance traveled (two-way ANOVA: *P* = 0.2718). (*B*) Time spent in the center (two-way ANOVA: *P* = 0.605). (*C*) Time spent in the RIM (two-way ANOVA: sex, F(1, 58) = 4.707, *P* = 0.0342; n = 18F/16F Ctrl and n = 12 M/16F cKO). (*D* and *E*) Novel object recognition test. (*D*) Preference index (%) for two identical objects (two-way ANOVA: *P* = 0.3910), and (*E*) novel object recognition (%) (two-way ANOVA: genotype, F(1, 44) = 10.02, *P* = 0.0028; n = 13 M/12F Ctrl and n = 10 M/13F cKO). (*F*) Sociability index (two-way ANOVA: *P* = 0.229; n = 18 M/16F Ctrl and n = 12 M/16F cKO). (*G*–*I*) Light-Dark Box Test. (*G*) Time spent in the lit compartment (two-way ANOVA: *P* = 0.1177). (*H*) Latency to enter the dark compartment (two-way ANOVA: *P* = 0.1677). (*I*) Frequency of light–dark transitions (two-way ANOVA: *P* = 0.3087; n = 18 M/16F Ctrl and n = 12 M/16F cKO). (*J*–*L*) Two-way active avoidance test. (*J*) Percentage of CR over days in both sexes (three-way ANOVA: genotype × day × sex, F(4, 224) = 2.555, *P* = 0.0398; n = 17 M/16F Ctrl and n = 12 M/15F cKO). (*K*) Percentage of CR over days in males (two-way RM ANOVA: days × genotype, F(4, 108) = 2.664, *P* = 0.0363; Šídák’s multiple comparisons test: day 2: cKO = Ctrl). (*L*) Percentage of CR over days in females (two-way RM ANOVA: days × genotype, F(4, 116) = 3.010, *P* = 0.021; Šídák’s multiple comparisons test: day 2: cKO < Ctrl, *P* = 0.0339; n = 17 M/16F Ctrl and n = 12 M/15F cKO. Data are expressed as the mean ± SEM. *P < 0.05, **P < 0.01.

Given changes in neuronal network activity and LTP may have consequences for behavioral outcomes and are linked with the processes of learning and memory formation (36), we performed a battery of tests encompassing a variety of behavioral domains in control littermate and cKO mice. We tested mice from the less to the more aversive tests and performed an open field (OF) test to assess locomotor activity, novel object recognition test (NORT) for cognitive performance, three-chamber sociability test for sociability, dark-light box (LB) for anxiety-relevant behavior, ending with a two-way active avoidance test (TWA) for aversive learning. When mice were tested in the OF arena, there were no genotype differences in locomotor activity (Fig. 5*A*), neither in the time spent in the more anxiogenic region of the arena at the center (Fig. 5*B*) nor at the lower anxiogenic region of the arena at the periphery (rim) (Fig. 5*C*); however, females of both genotypes spent more time in the lower anxiogenic part of the OF arena (see *SI Appendix,* Table S1 for the Statistics). For assessment of cognitive performance, we first checked in the training phase of the NORT that the animals, which were randomly assigned to an identical object pair, did not show a preference for one of the two identical object positions (Fig. 5*D*). After an intertrial time of 24 h, one of the two objects was replaced and performance in novel object recognition was calculated and found to be lower in both male and female cKO mice than the control littermate (Fig. 5*E*). Additional automatically annotated motion tracking analyses via DeepLabCut (37) revealed a general sex difference in mouse positioning relative to the objects over time (*SI Appendix,* Fig. S12). Then we found that neither genotype nor sex showed differences in preference toward a social target in the three-chamber sociability test (Fig. 5*F*) and in the time spent in the lit compartment of the LB (Fig. 5*G*), where there was equal first latency to the dark compartment (Fig. 5*H*) and a similar number of lit-dark compartment transitions in the LB test (Fig. 5*I*). Finally, both control and cKO mice in the TWA test learned the task over time and increased progressively the number of conditioned responses (i.e. to avoid foot-shock upon a tone presentation) (Fig. 5*J*), with an interaction between day of the test, sex, and genotype. To explain the roles of sex and genotype in this aversive learning test, we separated the learning curves of males (Fig. 5*K*) from females (Fig. 5*l*) and found that learning was poorer on day 2 in female cKO mice, which needed more trials to learn the task compared to the control littermates (Fig. 5*l*). Furthermore, when looking at the daily learning curves we found additional differences. The performance of both male and female cKO mice was worse on the second day of the test (*SI Appendix,* Fig. S13) and they were thus slower in learning the task. Importantly, these cognitive alterations were specific to GR deletion during postnatal development and were not observed following GR deletion in adulthood (*SI Appendix,* Fig. S14). The efficiency of the cKO in adulthood was confirmed by IF showing a reduction of GR in OPC (*SI Appendix,* Fig. S15). To control for the effects of tamoxifen injection, a batch of mice without tamoxifen-induced GR deletion was subjected to the same behavioral tests and showed no behavioral alterations (*SI Appendix,* Fig. S16).

These data demonstrate that loss of GR in OPCs in early life does not impact on anxiety-relevant behaviors, but leads to deficits in hippocampus-dependent nonaversive and aversive tasks for learning and memory in adulthood, under moderate stress activation of the HPA axis (38–41).

## Discussion

**Fig. 6.**
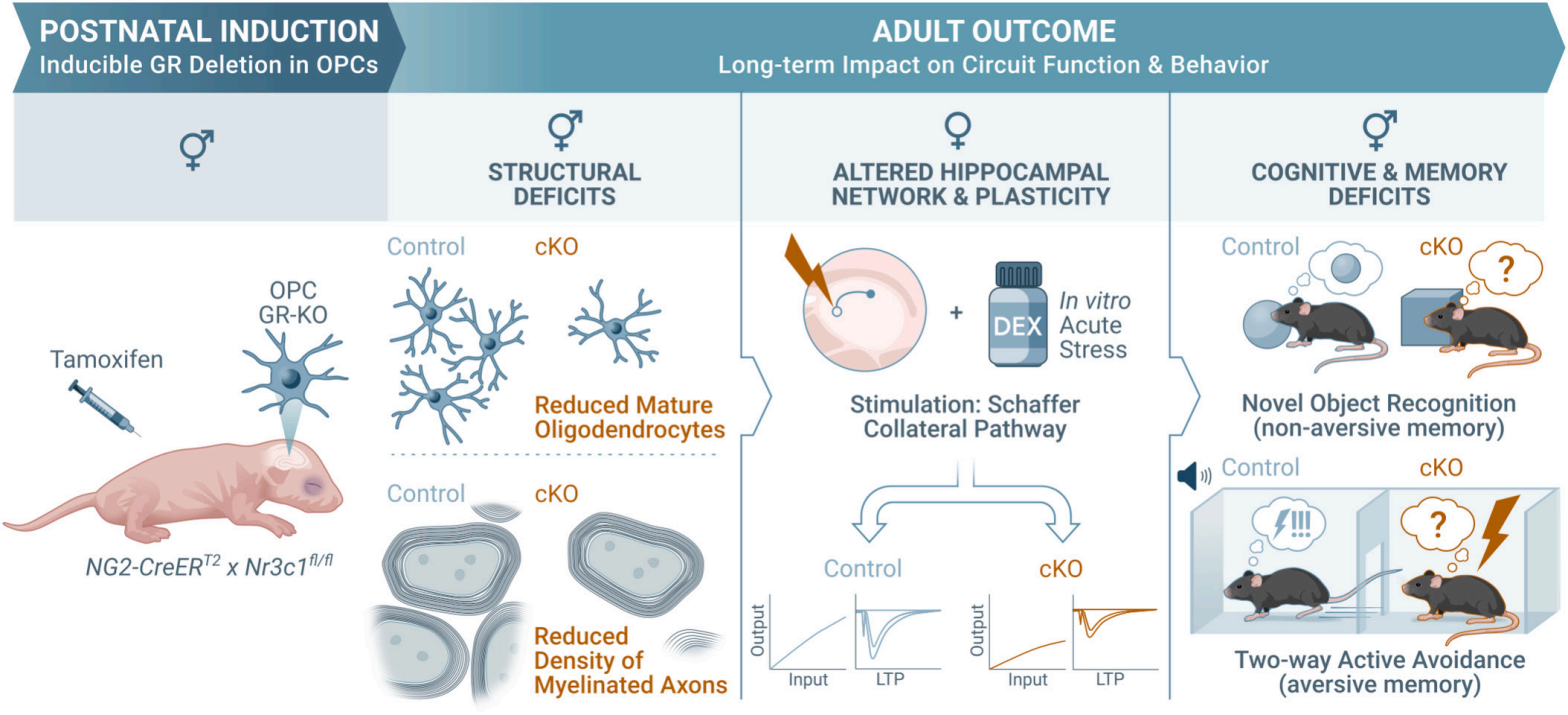
Graphical summary of the findings.

We established here a physiological role of GR-signaling in OPCs during postnatal brain development. Specifically, our findings demonstrate that early postnatal deletion of the GR in OPCs influences the density of hippocampal OLs and of myelinated axons in adulthood, alters neuronal network excitability and synaptic potentiation in response to dexamethasone challenge and affects aversive learning later in life. Collectively, our data highlight that constitutive expression of GR in OPCs is critical for maintaining a physiological OPCs-OLs ratio and for supporting hippocampal activity and plasticity and ensuring hippocampus-related cognitive function (Fig. 6).

First, we demonstrated that the cKO of GR in postnatal OPCs alters the density of OLs in the adult hippocampus, without affecting the density and proliferation of OPCs (Fig. 1). Previous studies have examined the role of GCs on the proliferation-maturation dynamics of the OLs in murine models. For instance, the exposure to ELA, which activates the HPA axis and elicits an increase in GCs (24, 42), leads to precocious OLs differentiation in pups, and is associated with a depletion of the OPCs pool in adult mice (22). These findings have also been corroborated by postmortem studies from human individuals with a history of childhood trauma (23). In contrast, in our experimental design, mice were not exposed to stress, and we focused instead on the physiological function of GCs by conditionally deleting GRs in OPCs during the early postnatal period. Our data suggest that physiological GC levels are essential for proper maturation of OPCs into OLs. Furthermore, GCs appear to play a key role in coordinating the timing of differentiation, as previously shown in vitro, via signaling clocks in precursor cells during development (43). In support of this role of GCs in vitro studies have shown differences in OPC proliferation and differentiation in response to corticosterone treatment (44) and reduction in myelination in mixed central nervous system cell culture after GC treatment (45). In our study, we did not detect changes in myelin ultrastructure and thickness or internode length (Fig. 2; *SI Appendix*, Fig. S8), but we observed a decreased density of myelinated axons in both male and female cKO mice (Fig. 2). A reduced density of myelinated axons has previously been reported in different models, including in postmortem tissue of patients with progressive multiple sclerosis but in normal-appearing white matter (46). It is tempting to speculate that the decrease in oligodendrocyte density is linked to the decrease in myelinated axon density, with consequences on the development of neuronal circuits.

While our approach represents a mouse model in which GRs have been cKO specifically in OPCs (Fig. 1), with an absence of recombination in other GR-expressing cell types in the CNS (*SI Appendix*, Fig. S4), off-target recombination has been reported for this inducible Cre line in other cell types (47). Therefore, our results may depend on limited off-target recombination in other cells that express NG2 and GR.

Postnatal GC concentrations are tightly controlled to shield the developing brain from stress-induced GC surges, a phenomenon known as the SHRP, during which moderate stressful challenges fail to trigger a significant stress hormone response (2). Our data highlight a critical role for GR signaling in OPCs during this postnatal period, when basal levels of GCs are extremely low. We conclude that postnatal OPCs depend on a delicate regulation of circulating GCs. Elevated levels of GCs, as observed during ELA, may disrupt OL lineage dynamics, resulting in changes associated with negative health outcomes. Conversely, maintaining homeostatic levels of GCs is likely crucial for supporting proper oligodendrocyte lineage maturation.

Beyond the canonical role of OPCs in sustaining oligodendrogenesis and myelination of axons, our data also revealed a sex-specific role of GR in OPCs in modulating neuronal network excitability and potentiation upon challenge. We demonstrated that acute application of the synthetic GC agonist, dexamethasone, increased fEPSPs, PPR in control female mice, but not in cKO mice. The dexamethasone-induced increase in fEPSP amplitudes observed in female control mice suggests, at least in part, changes in the postsynaptic function that are predominantly mediated by glutamatergic AMPA receptors. However, evoked fEPSP amplitudes do not represent isolated AMPA receptor-mediated responses and may also be influenced by other synaptic mechanisms. We also showed that female cKO mice have a lower LTP compared to control mice after incubation with dexamethasone. Importantly, as LTP was only assessed in the presence of dexamethasone, we cannot exclude the possibility that cKO mice exhibit a baseline impairment in LTP independent of the acute GC challenge. These data raised two important points: first, they revealed a clear sex-specific electrophysiological response under acute challenging conditions (i.e., ex vivo dexamethasone application), indicating that female mice might be more sensitive to slight increases in circulating stress hormone and potentially more responsive to HPA activity (48–51). Second, GR in OPCs appears to be necessary to induce changes in neuronal network excitability and synaptic potentiation in response to a mild and/or acute stressor. It has been shown previously that GCs modulate learning and memory processes. GCs also modulate synaptic transmission by slowly enhancing the amplitude of miniature excitatory postsynaptic currents (mEPSCs) (52) and rapidly facilitating synaptic potentiation in the mouse hippocampal CA1 area (53); however, these data were always obtained from patch-clamped neurons. Here, we have shown that a specific non-neuronal cell type is required for such excitability and it is mechanistically involved in synaptic plasticity, which may be relevant for memory and learning. Whether this phenomenon is mostly driven by the dorsal or ventral region of the hippocampus remains an open question that cannot be addressed through our dataset. Although dexamethasone is a potent GR agonist, it can also bind to mineralocorticoid receptors (MR) with low affinity. In our study, we did not perform additional experiments in the presence of a GR antagonist such as RU486, therefore, the possibility that MR receptors in neighboring neurons may also be activated in cKO mice as compensatory effects, due to the lack of GR in OPCs cannot be completely ruled out. However, previous studies have shown that dexamethasone at the dose used in our study induces LTP-related mechanisms that are completely blocked by RU486 (54), supporting a predominant role of GR in mediating these plasticity effects. Based on our data that showed changes in the density of myelinated axons and differences in network excitability and synaptic plasticity in response to dexamethasone, we speculate that postnatal deletion of GR in OPCs may have long-lasting consequences for neuronal network formation. Rechallenging such neuronal networks in the presence of dexamethasone is also impaired, possibly indicating a direct OPC–neuron interaction, conceivably involving GR signaling in OPCs.

The sex- and brain region-specific alterations observed ex vivo in the GR cKO mice in network excitability and LTP occurred coincidently with an impaired performance in the nonaversive memory test (NORT) and in an aversive learning paradigm (TWA), especially in female mice. In the NORT, mice are exposed to novelty, which can be arousing, but is unlikely to lead to substantial increase of circulating GCs (55). In the TWA, mice are exposed to a mild stressful situation which triggers the activation of the HPA axis (41). It has previously been shown that fear conditioning induces OPCs to proliferate and differentiate into myelinating oligodendrocytes (56) and that myelin formation is necessary for remote fear memory retrieval. Similar data were obtained for nonaversive learning, including motor learning or hippocampal-related learning and memory in the Morris water maze (57). Clinical data also support the importance of experience-dependent myelin formation. Indeed, structural MRI studies have shown that white matter changes are required for the acquisition of both new motor and cognitive skills (e.g., learning to read, juggling, piano playing) (58–60). Moreover, cognitive deficits have been associated with (dys)function of the oligodendrocyte lineage and alterations in myelin content and ultrastructure. Our cKO mice, which have a reduced density of OLs and of myelinated axons and impairment in LTP, showed deficits in the NORT and were slower than the controls in learning the task in the TWA. In these mice, the reduction in mature OLs was associated with a decrease in the density of myelinated axons, however the myelinated axons showed normal myelin content and g-ratio. This suggests that the extant OLs, despite being reduced in the cKO mice, may not be sufficient to myelinate all hippocampal axons. These changes in neuronal circuitries could impair baseline cognitive performance, such as those observed in the NORT, and may also affect the excitability and plasticity of the circuitry in response to dexamethasone treatment and of the behaviors of the mice under mild stressful situations, such those observed in the TWA.

GCs play a critical role in memory formation (61, 62): both memory-enhancing and -impairing effects have been reported (61, 63). Extending the classical view that GCs exert their cognition-modulating effects exclusively by modulating neuronal function, a recent study showed that the astrocyte-specific ablation of GR led to impaired aversive memory expression in two different paradigms of Pavlovian learning (64). Thus, it is tempting to speculate that proper memory formation, initiated by activation of the HPA axis, requires GR expression in OPCs during development. Specifically, GR signaling in OPCs may regulate the proper formation of neuronal circuitry and its capacity to respond to acute challenges later in life; disruption of this process could impair OPC-neuron communication with consequences on memory formation and learning in adulthood.

In conclusion, our data demonstrate that GRs are essential from the very early stages of postnatal development to regulate OL differentiation and axon myelination. While the specific developmental check-points of the GC pathway in OPC remain unexplored, we demonstrated that the loss of GRs in early life OPCs appears to prime hippocampal network excitability and plasticity and impair learning abilities, particularly under mild stress in adulthood. Based on our histological findings, it is apparent that the lack of GR in OPCs impacts on their canonical, myelin-related functions. However, the reduced responsiveness of the hippocampal network to dexamethasone application in cKO suggests that the GC pathway in OPCs might also be essential for noncanonical, myelin-independent regulation of network excitability. Future studies are needed to further clarify the conditions regulating each of these functions and how they orchestrate cognitive processes in response to environmental challenges.

## Materials and Methods

A succinct description of the experimental procedures related to the main results is provided below. Comprehensive materials and methods descriptions, including methods related to supplementary results, are reported in *SI Appendix*, *Supplementary Material and Methods*.

### Mice.

NG2-CreER^T2^ male mice (B6.Cspg4^tm1.1(cre/ERT2)Fki^) (47) were crossed with female Nr3c1^fl/fl^ (B6.Cg-Nr3c1^tm1.1Jda/J^, Jacksons Lab, #021021) to obtain NG2-CreER^T2^ x Nr3c1^fl/fl^ mice (cKO mice), which expressed tamoxifen-dependent Cre recombinase under the control of the NG2 transcriptional regulatory elements and Nr3c1 gene flanked by two loxP sites. NG2-CreER^T2^ × Nr3c1^fl/fl^ male mice were then bred with Nr3c1^fl/fl^ female mice to obtain the experimental mice. Littermate Nr3c1^fl/fl^ were employed as control (Ctrl).

#### Early postnatal deletion cohort.

Tamoxifen (Sigma-Aldrich, T5648) was dissolved in pure ethanol (Laborchem® international, LC-8657.1) and diluted in corn oil (Sigma-Aldrich, C8267) at a working concentration of 20 mg mL^−1^. All the pups included in the experiment received i.p. injection of 0.1 mg tamoxifen in 5 μL vehicle at PDs 2 and PD 4 (65). The mice were allowed to reach adulthood undisturbed, except for the conventional caretaking routine procedure in the mouse unit.

Genotyping was performed as described before (47) or adapted from the recommended protocol on the JAX datasheet via standard PCR of genomic DNA. Primer sequences are reported in the supplementary materials and representative genotyping output in *SI Appendix,* Fig. S2*A*. All experiments were then conducted on adult mice of both sexes (8 to 12 wk old). Mice of the same sex were group-housed (3 to 5 per cage) and maintained at a 12/12 h light–dark cycle (lights on at 7:00 A.M.), at controlled temperature and humidity (temperature = 22 ± 2 °C, relative humidity = 50 ± 5%). Food and water were available ad libitum. All experiments were performed in accordance with the European directive 2010/63/EU for animal experiments and were approved by the local authorities (Animal Protection Committee of the State Government, Landesuntersuchungsamt Rheinland-Pfalz, Koblenz, Germany).

### Flow Cytometry.

Naïve mice were perfused with PBS, and hippocampi and cortices were dissociated into single-cell suspensions. After filtration, density-gradient purification, and antibody staining, cells were analyzed by flow cytometry (FACSCanto II, BD Biosciences) and processed using FlowJo software. Detailed protocols and reagents are provided in *SI Appendix*, *Supplementary Material and Methods*.

### Immunohistochemistry.

Mice were transcardially perfused with PBS followed by 4% PFA. Brains were postfixed, cryoprotected in 30% sucrose, embedded in OCT, and sectioned coronally (30 to 35 μm) to include the somatosensory cortex and dorsal hippocampus. Free-floating sections were processed for immunohistochemistry as previously described (25). A detailed description of the workflow of the immunohistochemistry protocol and microscopy and image analysis is provided in *SI Appendix*, *Supplementary Material and Methods*.

### Electron Microscopy.

Freshly harvested brains were cut, fixed, postfixed with osmium tetroxide, dehydrated, and embedded in EPON. Semithin sections were used for orientation, and ultrathin sections (60 nm) were imaged by transmission electron microscopy. Axon diameter, myelin thickness (g-ratio), and myelinated axon density were quantified in FIJI. The g-ratio was defined as inner axon diameter divided by total outer diameter. At least 100 axons per region per mouse were analyzed, excluding artifacts and nontransverse sections. Detailed experimental procedures, reagents, and analysis are provided in *SI Appendix*, *Supplementary Material and Methods*.

### Electrophysiological Recordings: MEA.

Naïve 9- to 12-wk-old mice that received tamoxifen at PDs 2 and 4, were used. After deep anesthesia, animals were perfused with choline-based artificial cerebrospinal fluid (c-ACSF). The brains were removed and placed into ice-cold c-ACSF perfused with carbogen and sectioned into 350 μm horizontal hippocampal slices. Slices were recovered in normal ACSF for at least 40 min before recording. Recordings were performed on a multichannel MEA system (60MEA200/30iR; MEA2100 System, MCS GmbH) with continuous perfusion of carbogenated ACSF containing 0.01% ethanol. The stimulating electrode was positioned on the CA3 region and the recording electrode in the CA1 along the Schaffer Collateral pathway. All recording protocols were generated and applied by Multi Channel Experimenter 2.2 software. Details regarding the measurement of spontaneous neuronal activity, evoked activity, and paired-pulse stimulation with and without dexamethasone treatment are reported in *SI Appendix*, *Supplementary Material and Methods*.

### LTP recordings.

Female control (N = 3) and female cKO (N = 5) mice of 9 to 12 wk were used. Slices were prepared and treated as above and continuously perfused with ACSF containing 200 nM dexamethasone (0.01% ethanol). After 10 min of baseline recordings in the CA1 region, with an interstimulus interval of 60 s in the CA3 region, a 100 Hz high-frequency stimulation (HFS) was given to induce LTP. This was followed by 60 min of baseline-intensity stimulation, every 60 s. The level of HFS-induced LTP was analyzed by normalizing the mean amplitudes from the last 10 min of baseline recordings after HFS (50 to 60 min post-HFS) to the mean of the amplitude of the initial 10 min baseline recordings before the HFS. The data from the control mice and the cKO mice were compared against pooled recordings from the independent control pathway that was generated by a second stimulation electrode located in the direction of the subiculum, as it cannot generate long-term plasticity changes in CA1. For details, see *SI Appendix*, *Supplementary Material and Methods*.

### Behavioral Testing.

Mice were handled for 2 min daily for 3 to 5 d before the start of the testing. The latter was performed between 8:00 A.M and 2:00 P.M, in sound-attenuating boxes under consistent lighting conditions (37 lx), except for the light–dark box test (600 lx). All mice were randomized and experimenters were blinded during the tests. The behavioral tests were videotaped (Basler camera) and mouse performance automatically tracked with EthoVisionXT software 15.0 (Noldus). Manual scoring of the recorded behavior was performed by a blinded experiment using The Observer XT12 software (Noldus Information Technology). The tests were performed in the following order: OFT, NORT, three-chambers social interaction test, LD Box Test, and TWA test. Detailed descriptions of the protocols are provided in *SI Appendix*, *Supplementary Material and Methods*.

### Statistical Analysis.

Analyses were performed using GraphPad Prism 10.0 and R (v4.4.2). Data are presented as mean ± SEM. Statistical tests were selected according to data distribution and experimental design, with significance set at *P* < 0.05. Detailed information of the statistical analyses is provided in the figure legends and in *SI Appendix,* Table S1.

## Supplementary Material

Appendix 01 (PDF)

## Data Availability

All custom codes used for data analysis are provided in the *SI Appendix*. The code used for the analysis of the Node of Ranvier is publicly available at GitHub (66).
