# Supplementary material for: Glucocorticoid receptors in oligodendrocyte precursor cells regulate hippocampal network plasticity and stress-induced behavior in mice
Source: Proc Natl Acad Sci U S A. 2026 Jul 20;123(30):e2614867123. doi: 10.1073/pnas.2614867123 (PMC13416226; doi:10.1073/pnas.2614867123)
Supplement: Supplementary file 1 — Appendix 01 (PDF) [file pnas.2614867123.sapp.pdf]

PNAS

Supporting Information for

Glucocorticoid receptors in oligodendrocyte precursor cells regulate hippocampal network plasticity and stress-induced behavior in mice

Authors:

Lorenzo Mattioni[1,2]*, Giulia Poggi[1,3]*, Celine Gallagher[4]*, Katrin Becker[3,5], Linh Le[3], Maja Papic[6], Jasmin Engbers[3], Maija-Kreetta Koskinen[7,8], Ali Abdollahzadeh[9], David P. Herzog[3], Leonardo Nardi[1], Andrea Conrad[10], Sarah Winterberg[11], Christa Merte-Grebe[12], Liana Melo-Thomas[11], Hyonseung Lee[11], Hans Schwarzbach[12], Jennifer Klüpfel[3,5], Ralf Kinscherf[12], Jan Engelmann[3], Beat Lutz[5,10], Ari Waisman[6], Iiris Hovatta[7,8], Thomas Mittmann[4], Michael J. Schmeisser[1,2], Marianne B Müller[2,3,5]*, Giulia Treccani[1,3,11]*

1. Institute of Anatomy, University Medical Center of the Johannes Gutenberg-University Mainz, Mainz, Germany

2. Focus Program Translational Neurosciences, University Medical Center of the Johannes Gutenberg-University, Mainz, Germany

3. Department of Psychiatry and Psychotherapy, University Medical Center of the Johannes Gutenberg-University Mainz, Mainz, Germany

4. Institute of Physiology, University Medical Center of the Johannes Gutenberg-University, Mainz, Germany

5. Leibniz Institute for Resilience Research, Mainz, Germany

6. Institute for Molecular Medicine, University Medical Center of the Johannes Gutenberg-University Mainz, Mainz, Germany

7. SleepWell Research Program, Faculty of Medicine, University of Helsinki, Helsinki, Finland

8. Department of Psychology, Faculty of Medicine, University of Helsinki, Helsinki, Finland

9. A. I. Virtanen Institute for Molecular Sciences, University of Eastern Finland, Kuopio, Finland

10. Institute of Physiological Chemistry, University Medical Center of the Johannes Gutenberg University

11. Department of Systemic Neuroscience, Institute of Anatomy and Cell Biology, Philipps University, Marburg, Germany

12. Department of Medical Cell Biology, Institute for Anatomy and Cell Biology, Medical Faculty, Philipps University Marburg, Marburg, Germany

* Indicates equal contributions

Corresponding author

Univ.-Prof Dr. Giulia Treccani

Department of Systemic Neuroscience

Institute of Anatomy and Cell Biology

University of Marburg

Robert-Koch-Straße 8

35032 Marburg, Germany

Tel. 06421-28 26507

Email: treccani@staff.uni-marburg.de

This PDF file includes:

Supporting text

Figures S1 to S16
Tables S1
SI References

Supplementary material and methods

NG2-CreER^{T2} x Nr3c1$^{fl/fl}$ mouse line genotyping

Genotyping of NG2-CreER^{T2} mice was performed using PCR with genomic DNA extracted from ear and tail samples. The following primers were used: 1 (5′ - GGC AAA CCC AGA GCC CTG CC - 3′), 2 (5′ - GCT GGA GCT GAC AGC GGG TG - 3′), and 3 (5′ - GCC CGG ACC GAC GAT GAA GC - 3′). These primers distinguish between the NG2 wild-type allele (557 bp) and the mutant allele (829 bp). Genotyping for Nr3c1$^{fl/fl}$, to check for the presence of the loxP alleles (fl/fl) flanking the Nr3c1 gene (GR) and to confirm the deletion, the following primers were used: 1 (5′ - ATG CCT GCT AGG CAA ATG AT - 3′), 2 (5′- TTC CAG GGC TAT AGG AAG CA - 3′), and 3 (5′ -TGG TTG TTG GTG CTT TTG GCT AAA TC - 3′). These primers distinguish between homozygosity of the GR$^{wt/wt}$ alleles (250 bp), the GR$^{fl/fl}$ alleles (300 bp) and for the deletion (del) of the GR gene (GR fl/del), which shows an additional band at 400 bp (*SI Appendix*, Fig. S2 A).

Mice

Adult GR deletion cohort. Tamoxifen (TAM, Sigma-Aldrich, T5648) was dissolved in pure ethanol (Laborchem® international, LC-8657.1) and diluted in corn oil (Sigma-Aldrich, C8267) at a working concentration of 20 mg ml^{-1}. All the young adult (> P60) mice included in the experiment received 75 mg/kg TAM daily for 5 consecutive days via i.p. injection. Body weight and physical status were monitored throughout. The mice were left undisturbed for additional 3 weeks, to allow for recombination and complete depletion of the endogenous GR.

Mouse line control cohort. To control for any confounding effects from the lack of one NG2 allele, independently of the deletion of GR, and to validate the selected control group, male and female adult (>10-week-old) NG2-CreER^{T2} x Nr3c1$^{fl/fl}$ and Nr3c1$^{fl/fl}$ mice underwent physical and behavioral assessment, without TAM treatment.

Flow cytometry

Naïve mice were sacrificed with isoflurane (Piramal Critical Care Deutschland; Cat# 4150097146757) and perfused with ice-cold 0.1 M pH 7.4 phosphate buffer saline (PBS) (ThermoFisher Scientific, 10010023). The hippocampi and cortices were rapidly dissected and coarsely chopped in Hank's Balanced salt solution (HBSS + $CaCl_2$, $MgCl_2$) containing 1 mg/ml of papain (from papaya latex, dnase I Sigma-Aldrich, 51807363), including 40 µg/ml of DNase I (grade II from bovine pancreas, Sigma-Aldrich, 10104159001 (51743558)). Tissue was dissociated using gentleMACS™ Octo Dissociator (Miltenyi) using a built-in ready-to-use program optimized for small quantities (<100 mg) of the CNS tissue. The cells were then resuspended in 8 ml DMEM/ 1% horse serum and filtered through 70-µm cell strainers. The resulting cell pellet was then re-suspended in 25% Percoll solution (Cytiva) and centrifuged (25 min, room temperature (RT), 300 *g*). Cells were resuspended in MACS Neuro Medium (Miltenyi Biotec, 130-093-570) containing 1x MACS NeuroBrew-21 (Miltenyi, 130-093-566), 100 U/ml Penicillin Streptomycin (Thermo Fisher Scientific, 15140122) and 2 mM L-Glutamine (Thermo Fisher

Scientific, 25030-024 (51709381), and incubated with slow rotation at 37 °C for two hours. After washing the cells in FACS buffer (0,5% bovine serum albumin (PAN-Biotech P06-1391500 (51809570)), 2 mM Ethylenediaminetetraacetic Acid (EDTA, Sigma-Aldrich) in PBS), Fc receptors were blocked for 10 min with Fc-block (1:100 in FACS buffer, BioXCell, BE0307). Surface antigens were then labelled for 20 min on ice with FACS buffer containing following reagents: 1:1000 Fixable Viability Dye eFluor™ 780 (eBioscience, 65-0865), PE/Cyanine7 anti-mouse/human CD11b 1:1000 (BioLegend 101216), Brilliant Violet 510™ anti-mouse CD45 (BioLegend, 103138) and CD140a (PDGFRA) APC 1:10 (Miltenyi Biotec, 130-102-473). For intracellular staining cells were fixed and permeabilized with eBioscience™ Foxp3/transcription factor staining buffer set (ThermoFisher Scientific, 00552300) according to the manufacturer's instructions. Cells were analyzed by using a FACSCanto II device (BD Biosciences). Post-acquisition analysis was performed by using FlowJo software. A list of the employed antibodies is provided in the list of employed antibody section.

Protein quantification via western blot

The samples were collected from mice that underwent behavioral testing. The entire cortex and hippocampus of 7 control (Ctlr) (4 males (M), 3 females (F)) and 7 cKO (4M, 3 F) were dissected, homogenized in ice-cold RIPA lysis buffer (ThermoFisher Scientific, 89900) and protease and phosphatase inhibitors (Roche, 04906837001, 04693132001), and centrifuged at 12,000 rpm for 20 min at 4 °C. Supernatants were collected and protein concentration assessed through

bicinchoninic acid (BCA) assay (ThermoFisher Scientific, 23225). The samples were diluted in 4X Laemmli-buffer and incubated at 95°C for 5 minutes, except for the samples probed with oligodendroglial proteolipid protein (PLP) antibody, which were incubated with β-mercaptoethanol at 65°C. A total of 10-25 µg protein per sample (Fig.S6-S7) was separated on sodium dodecyl sulfate-polyacrylamide gel electrophoresis (SDS-PAGE, precast gels Bio-Rad, L007043). Proteins were then electrotransferred onto nitrocellulose membranes (GE Healthcare Life science, 10600002) in ice-cold transfer buffer and stained for total protein using Revert™ protocol (LiCor Biosciences). The membranes were blocked with 5% non-fat milk powder dissolved in Tris-buffered saline containing 0.05% Tween-20 (TBST) for 1 h at RT and subsequently incubated in primary antibody diluted in blocking solution at 4°C ON. The membranes were washed three times in TBST and then incubated with secondary antibody, diluted 1:10,000 in blocking solution, for 1 hour at RT. After three washes in TBST, signals were acquired using Odyssey Fc imaging system and image Studio 5.0 (LiCor Biosciences). Signal intensity for each sample was quantified by densitometric analysis and normalized to the corresponding total protein lane using Empiria Studio® Software 2.0 (LiCor Biosciences) (1). Data are expressed as a percentage of Ctrl. A list of the employed antibodies is provided in the List of employed antibody section.

List of employed antibodies

AB	Company | Provider	Cat. Nr. | ID	Dilution
**Flow Cytometry**			
mouse anti-CD140a (Pdgfra)	Miltenyi Biotec	130125521	1:10

rat anti-CD45	BioLegend	103138	1:200
rat anti-CD11b	BioLegend	101215	1:1000
mouse anti-GR	Santa Cruz Biotechnology	sc-393232	1:500
chicken anti-CNPase	Sigma-Aldrich	AB9342	1:1000
goat anti-mouse	Sigma-Aldrich	SAB4600066	1:500
donkey anti-chicken	Sigma-Aldrich	SAB4600031	1:500
**Histology**			
Primary			
rat anti-AN2	Kind gift from Prof. J. Trotter, University of Mainz		1:250
mouse anti-GR	Santa Cruz Biotechnology	sc-393232	1:250
goat anti-Olig2	RD Systems	AF2418	1:500
rabbit anti-ASPA	Kind gift from Lutz Lab, University of Mainz		1:1000
rabbit anti-NeuN	Abcam	ab177487	1:100
rabbit anti-GFAP	Dako	Z0334	1:500
rabbit anti-Iba1	Wako	019-19741	1:1000
rabbit anti-PDGFRα	Cell Signaling Technology	3174	1:200
mouse anti-PDGFRβ	R&D Systems	AF1042	1:500
rat anti-Ki-67	ThermoFisher Scientific	SolA15.	1:200
mouse anti-CASPR	NeuroMab	#75-001	1:1000
rabbit anti-Nav1.6	Alomone labs	#ASC-009	1:250
rat anti-Mbp	Serotec	MCA409S	1:200
Secondary			
goat anti-chicken	Aves Labs	F1005	1:500
goat anti-rabbit	Invitrogen	A11011	1:500
goat anti-mouse	Life Technologies	A11001	1:500
goat anti-rabbit	Life Technologies	A11008	1:500
goat anti-mouse	Invitrogen	A21236	1:500
donkey anti-rabbit	Invitrogen	A31573	1:500
donkey anti-goat	Life Technologies	A21432	1:500
donkey anti-mouse	Life Technologies	A21202	1:500
donkey anti-rabbit	Life Technologies	A21206	1:500
goat anti-rat	Life Technologies	A21247	1:500
donkey anti-mouse	Life Technologies	A31571	1:500
goat anti-mouse	Abcam	ab150115	1:400
goat anti-mouse	Life Technologies	A28175	1:400
donkey anti-rat	Life Technologies	A21208	1:500

Western Blot			
Primary			
rabbit anti-NG2	Merck Millipore	AB5320	1:500
rabbit anti-ASPA	Merck Millipore	ABN1698	1:1000
mouse anti-CNPase	Merck Millipore	C5922	1:500
rat anti-Myelin Basic Protein	Merck Millipore	MAB386	1:1000
rat anti-PLP	Kind gift from Prof. E-M. Krämer-Albers(2) University of Mainz	cloneaa3	1:100
Secondary			
goat anti-rat	LiCor Biosciences	92568076	1:10000
goat anti-rat	LiCor Biosciences	92532219	1:10000
goat anti-rabbit	LiCor Biosciences	92532211	1:10000
goat anti-mouse	LiCor Biosciences	92532210	1:10000

Immunohistochemistry

Mice were anesthetized via i.p. of Ketamin/Xylaxin (200 mg/kg, 20 mg/kg) and intracardially perfused with ice-cold PBS followed by 4% paraformaldehyde (ChemCruz). Brains were post-fixed in 4% PFA at 4°C for 2 hours, with the exception of Pdgfra and Ki-67 staining and for Caspr, Mbp and Nav1.6 (post-fixed 24 hours), then transferred into 30% sucrose in 0.1 M PBS, pH 7.4 for 48 h and afterwards embedded in a cryoprotective medium (Tissue-Tek, O.C.T., 4583, Sakura Finetek Europe). Free-floating coronal sections (30-35 µm) including the somatosensory cortex and dorsal hippocampus (bregma from -1.79mm to -2.53mm) were washed in PBS (3x20min), then incubated for 90 minutes in 0.5% (v/v) Triton X-100 in, followed by 1 hour of blocking and permeability with 10% goat or donkey serum in 0.5% Triton X-100 in PBS at RT. Pdgfra and Ki-67 and Mbp/Caspr1 immunostaining slices were permeabilized and blocked for 60 min

and 150 minutes, respectively. The sections were then incubated overnight (ON) at 4°C in primary antibody, diluted in 3% serum in 0.5% Triton X-100 in PBS. On the following day, sections were washed in PBS (3x20 min) and incubated for 2 hours at RT with secondary antibody diluted in 3% serum in 0.5% Triton X-100 in PBS. The sections were then washed in PBS (3 x 20 min), counterstained with DAPI (1:10000 in ddH20, SIGMA, D9542) and mounted on SuperFrost slides (Epredia, K5800AMNZ72) with fluorescence mounting medium (Dako, S3023). For the paranodes and nodes of Ranvier immunohistochemistry, free-floating coronal sections (30-35 μm) including the somatosensory cortex and dorsal hippocampus (bregma from -1.79 mm to -2.53 mm) were washed in PBS (3x10 min), blocked in 5% NGS, 2,5% BSA, 0,25% TritonX-100 in PBS for 1 hour at RT and incubated ON at 4°C in primary antibody diluted in 5%NGS, 2,5% BSA, 0,25% TritonX-100 in PBS. On the following day, sections were washed in PBS (4x10 min) and incubated for 2 hours at RT with secondary antibodies diluted in 5%NGS, 2,5% BSA, 0,25% TritonX-100 in PBS. The sections were then washed in PBS (4 x 10 min) and mounted on slides with Vectashield plus DAPI (4',6-diamidino-2-phenylindole) mounting medium (#H-1200, Vector Laboratories). The used antibodies were: rabbit anti-Nav1.6 (1:250, #ASC-009, Alomone labs), mouse anti-CASPR (1:1000, #75-001, Neuromab), goat anti-rabbit Alexa Fluor 594 (1:500, 111-585-003, Jackson Immuno Research), donkey anti-mouse Alexa Fluor 488 (1: 500, 715-545-150, Jackson Immuno Research). A list of the employed antibodies is provided in the List of employed antibody section. Naïve mice were employed for proliferation and PDGFRA+ cells density assessment and for the analysis of

paranodes and nodes of Ranvier length; the remaining samples were collected from mice that underwent behavioral testing (see Fig. 1 *A*).

Microscopy and image analysis

Images were acquired at a Leica TCS SP8 confocal laser scanning microscope. For cell quantification (density or percentage), confocal micrographs were acquired with a 20x/0.75 (HC PL APO IMM CORR CS2) or 40x/1.30 (HC PL APO OIL CS2) immersion objectives, at a format of 1024 × 1024 pixels. The pinhole was set to 1 AU, the field of view ranged from 276.09 µm × 276.09 µm to 87.87 µm × 87.87 µm, and the voxel size from 270x 270 x 1000 nm^3 (xyz) to 637nm x 637nm x 1000 nm^3 (xyz). For each mouse, 4-6 micrographs (2-3 brain sections) from the dorsal CA1 and L2/3 of the somatosensory cortex, were acquired and Z-stack images were processed and analyzed with ImageJ (NIH) software by a blinded experimenter. Recombination efficiency was calculated as the proportion of $AN2^+Olig2^+$ cells co-expressing GR. Recombination specificity was estimated based on the proportion of neurons ($NeuN^+$), microglia ($Iba1^+$), astrocytes ($GFAP^+$), and the area covered by pericytes ($PDGFRB^+$) co-expressing GR. Cell density was calculated as the number of cells per unit area (cells/mm^2) by dividing the total cell count by the area of the region of interest (ROI). OPCs proliferation was calculated as the percentage of $PDGFRA^+$ cells co-expressing Ki-67^+. Only cells with a detectable DAPI signal were included in the analysis. For OPCs morphology analysis, confocal micrographs were acquired with a HC PL APO CS2

63x/1.40 oil objective; pinhole was set to 1AU, the image format to 1024 x 1024 pixel, the pixel size to 86nm x 86nm and the z-stack interval to 150nm.

Myelin content and internode length

For the analysis of myelin content and internode length, confocal micrographs were acquired with CS2 63x/1.40 oil objective, at a format of 1024 × 1024 pixels, the pixel size to 101nm x 101nm and the z-stack interval to 150nm. The pinhole was set to 1 AU. The size of the overall field of view, acquired via tail scan, was 288.50um x 195.33um. For each mouse, 2 micrographs (1 brain section, both hemispheres) from the dorsal CA1 and L2/3 of the somatosensory cortex, were acquired and Z-stack images were processed and analyzed with ImageJ (NIH) software by a blinded experimenter (2) and with IMARIS Software (https://imaris.oxinst.com). For the overall myelin content quantification, the optical plane with the maximum mean grey value was identified. This optical plane and the 10 optical planes above and below were included in the analysis, so that 6 µm of the total section thickness was used for quantification. Semi-automated segmentation of Mbp+ fibers was performed via a custom-written macro and the area covered by the MBP+ signal was employed as proxy for myelin content (3). For the analysis of the internode length, semi-automated 3D surface reconstruction of Mbp signal was performed in IMARIS. Internodes flanked by paranodal structures at both extremities were manually identified and labelled by a blinded experimenter. The length of this subset of paranode-flanked internodes was estimated via *BoundingBoxOO length C* parameter.

Analysis of paranodes and nodes of Ranvier

For the analysis of paranodes and nodes of Ranvier, imaging was performed with ZEISS LSM 880 Confocal Laser Scanning microscope with AiryScan (Zeiss). To image individual nodes within a field of view, a region around a node was cropped, and a z-stack of the cropped region was acquired. Z-stacks were acquired and pixel size set at 0.04 × 0.04 × 0.10 μm (x,y,z). For each mouse, 4-5 brain sections including CA1 region were analysed. An automated pipeline to segment and analyze the length of paranodes and nodes of Ranvier in the acquired 3D microscopy images was implemented (4).

OPC morphology: confocal acquisition and analysis parameters

For the confocal acquisition, the field of view was located so that the soma of the NG2-cell of interest was in the center of the micrograph. A minimum of ten cells for each mouse was acquired by a blinded experimenter. The semi-automated segmentation of the cell processes was performed with IMARIS software 10.2.0 (https://imaris.oxinst.com) through the Filament Tracing Wizard (Detection type: Starting Point; Starting point diameter: 10um) combined with AI-training based-Seed Point Classification (Segment Seed Point diameter: 0.5um). The reconstruction accuracy was evaluated by a blinded experimenter and manual refinement of the segmentation was carried out in the rare cases that required it. A final filtering based on the *filament length sum* was employed to exclude from the analysis any artefact and/or process that did not belong to the analyzed cell. The employed algorithm settings are outlined below.

[Algorithm]
Name = Autopath (no loops)
Segment Start Point = true
Detect Spines = false
Enable Regions of Interest = false
Track (over time) = false
[Soma Starting Points]
Segment Channel Index = 2
Segment Starting Point Diameter = 10.0 μm
Calculate Soma Model = true
[Filter Starting Points]
Segment Starting Point Threshold Low = 9.321
Segment Starting Point Threshold High = Automatic
Render Soma Model = false
[Seed Points for Segments]
Segment Seed Point Diameter = 0.500 μm
[Filter Seed Points for Segments]
Segment Seed Point Threshold = 33.886
Diameter around Starting Point(s) to remove Seed Points = 15.0 μm
Segment Diameter Filter Strengthness = 2
[Seed Points Classification]
Group Name = Filter
Input = All Spot
No. of Classes = 2
Class:: Name = Keep
Color = 0.000 1.000 1.000
Class:: Name = Discard
Color = 1.000 0.000 0.000
FilterType = ML
Training Data
Class:: Name = Keep
Size = 22
Class:: Name = Discard
Size = 48
[Segment Classification]
Group Name = Filter
Input = All Segment
No. of Classes = 2
Class:: Name = Keep
Color = 0.000 1.000 1.000
Class:: Name = Discard
Color = 1.000 0.500 0.000
FilterType = ML
Training Data
Class:: Name = Keep
Size = 93
Class:: Name = Discard
Size = 63
[Tree Build]
[Terminal Segment Postfilter]

Electron microscopy

Freshly harvested brains were cut into ~1.5 mm thick slices and fixed in a solution containing 2.5% glutaraldehyde, 1.25% paraformaldehyde, 0.05% picric acid in 0.1 M cacodylate buffer pH 7.2, for a minimum of 24h and then trimmed into smaller areas containing the region of interest (hippocampus with cornu ammonis), washed in a 0.1 M cacodylate buffer and then afterwards post-fixed in 2% Osmium tetroxide, dehydrated with ethanol/propylene oxide, and embedded in glycidyl ether 100 (EPON 812, SERVA Electrophoresis GmbH, Heidelberg, Germany). 0.5 µm semi-thin sections were collected, stained with toluidine blue pyronin and trimmed again in smaller regions. The EPON blocks were then cut using a Reichert Ultracut S ultramicrotome (Leica Microsystems, Wetzlar, Germany) and ultrathin sections (60 nm) were mounted on copper grids, contrasted with 4% uranyl acetate and lead citrate to confirm the brain region and observed with a Zeiss EM 10 C transmission electron microscopy (Carl Zeiss GmbH, Jena, Germany). Images were taken with ImageSP System (SYSPROG, Minsk, Belarus) from each section with a scale of 4.92 nm/ pixel. Myelin thickness (g-ratio), axon diameter and myelinated axon density were quantified manually using FIJI software. The myelin g-ratio was calculated as the ratio of the inner axon diameter (including non-compacting myelin)/ outer axon diameter (including the myelin sheath). For each image, all g-ratios were averaged, and these image means were subsequently averaged across all images collected from a given animal. All the axons contained in the acquired micrographs – total ≥100 axons/region/mouse - were quantified, except for any myelinated axons that presented obvious fixation artefacts or did not have a coronal cut, which were excluded from the analysis.

Electrophysiological Recordings – Multielectrode Array

Naïve mice were employed for this experiment. Three adult mice per group and per sex were employed. For each mouse, 3-9 paired recordings were taken, with each recording coming from a new individual slice. The mice were deeply anesthetized (i.p. 200 mg/kg ketamine) and transcardially perfused with choline-based artificial cerebrospinal fluid (cACSF) solution containing in mM: NaCl (87), KCl (2.5), $NaH_2PO4+H2O$ (1.25), choline chloride (37.5), NaHCO3 (25) and d-glucose (25), CaCl *$2H_2O$ (0.5), $MgCl_2$ (7) (pH of 7.4). The mouse was then decapitated, and the brain was quickly removed and placed into icy (4°C) cACSF perfused with carbogen (95% oxygen (O_2) and 5% carbon dioxide (CO_2)). After 1 minute, 350μm-thick sections were collected and placed in normal ACSF, containing (in mM) NaCl (125), KCl (3), MgSO4 x 7H2O (1.3), CaCl2 x H2O (2.5), NaH2PO4 x H2O (1.25), NaHCO3 (26) and d-glucose (13), at RT for at least 40 minutes before use. Individual slices were then transferred to a multielectrode array (MEA) chip of a two-chamber MEA system (MEA2100 System, Multi-Channel Systems MCS GmbH; Chip: 60MEA200/30iR). Here, the slice was constantly perfused with carbogenated normal ACSF [125 mM NaCl, 3 mM KCl, 1.3 mM $MgSO_4$ x $7H_2O$, 2.5 mM $CaCl_2$ x H_2O, 1.25 mM NaH_2PO_4 x H_2O, 26 mM $NaHCO_3$ and 13 mM d-glucose at a temperature of 32°C. The normal ACSF also contained 0.01% EtOH to control for any ethanol-induced electrophysiological changes (see below). Each slice was precisely placed to position a stimulating electrode in the CA3 region and a recording electrode in the CA1 region of the hippocampus, along the Schaffer Collateral pathway. A platinum grid was placed

on top of the slice to ensure electrode-tissue contact before starting the recordings. All electrophysiological recording protocols were generated and applied by Multi Channel Experimenter 2.2 software (Multi Channel Systems MCS GmbH), which had a 50 kHz sampling rate and a 200Hz cut-off Butterworth highpass 2.0 Order Filter. The recording of the reported outputs was performed and quantified via Multi-Channel Analyzer 2.2 (Multi Channel Systems MCS GmbH). Spontaneous neuronal activity was measured by recording extracellular spike frequency whereby an event counted as a spike exceeding the downward threshold of five standard deviations from baseline activity. Events were recorded for 5 minutes and quantified by the Multi Channel Analyzer 2.2. For evoked activity and FV analysis, an input-output curve was generated by applying step-wise voltage stimulations that ranged from 0.5V to 5V in 500mV steps. The maximum amplitude of both the FV and each evoked fEPSP was recorded by the electrode in the CA1 region for each slice. The paired-pulse stimulation protocol, which had a 50ms inter-stimulus interval, used the voltage stimulation intensity which produced an fEPSP equal to ~30% of the maximum evoked fEPSP amplitude from the input/output curve protocol, for each slice. After, the perfusion input was switched to carbogenated ACSF containing 200nM of Dexamethasone diluted in ethanol (0.01%). After wash-in of 30 minutes all protocols were repeated. On finishing, the slice was removed and the drug-infused ACSF completely washed out, before placing the next slice. To verify that detected spontaneous spiking activity relied on sodium channel function and not electrical noise or artefacts, tetrodotoxin (TTX) at a

concentration of 1μM was washed on for a further 10 minutes, resulting in a silencing of spiking activity

For LTP experiments, female control (N = 3) and female cKO (N = 5) mice of 9-12 weeks were anaesthetised and the brain removed, sliced and incubated in the same way as above stated. 200nM of Dexamethasone diluted in ethanol (0.01%) was consistently present in the ACSF perfusion of the slices. Again, a voltage stimulation intensity which produced an fEPSP equal to ~30% of the maximum evoked fEPSP amplitude from the input/output curve protocol was used in the CA3 region, for each slice. After 10 minutes of baseline recordings in the CA1 region, with an interstimulus interval of 60 seconds in the CA3 region, a 100 Hz high frequency stimulation (HFS) was given to induce LTP. This was followed by 60 minutes of baseline intensity stimulations, every 60 seconds. The level of HFS-induced LTP was analysed by normalising the mean amplitudes from the last 10 minutes of baseline recordings after HFS (50-60 min post-HFS) to the mean of the amplitude of the initial 10 minutes baseline recordings before the HFS. The data from the control mice and the cKO mice were compared against pooled recordings from the independent control pathway that was generated by a second stimulation electrode located in the direction of the subiculum, as it cannot generate long-term plasticity changes in CA1. The Multi Channel Analyzer 2.2 (Multi Channel Systems MCS GmbH) software was used to record the peak value of each fEPSP amplitude. All data were analysed using the Multi Channel Analyzer 2.2 software, Microsoft Office Excel (Microsoft), and GraphPad Prism (GraphPad Software).

Behavioral testing – detailed

Open field test (OFT). The mice were placed in the center of a square, non-transparent polyvinyl chloride (PVC) arena (44 cm x 44 cm x 40 cm) and allowed to explore freely for 10 minutes. Total distance travelled was scored.

Novel object recognition test (NORT). In the same OFT arena, the experimental mouse was allowed for 10-min exploration of two identical objects placed at equidistant locations (*training phase*) and was then returned to the homecage. After 24-hour, the mouse was reintroduced into the arena for 5-min exploration of one familiar and one novel object, which were similar in size but differed in shape, material, texture, and contrast (*testing phase*). Object exploration was defined as direct or proximal interactions with the object, including sniffing or touching it with the nose or forepaws. The ratio of time spent exploring the novel object to the total exploration time of both objects was calculated as the novel object recognition index (expressed as a percentage). Trials in which the total exploration time was less than 5 seconds were considered insufficient and excluded from the analysis.

Three-chambers social interaction test. The three-chambers social interaction test was conducted as previously described (5). Briefly, the experimental mouse was habituated for 5 minutes to the central chamber of a three-chambered apparatus. Afterwards, while the experimental mouse was briefly returned to the homecage, a same-sex juvenile (or young adult in case of the *adult deletion cohort*) C57BL/6 mouse (social target) was placed under one mesh cylinder located in one of the

two outer chambers of the apparatus and a metal ring (non-social target) under a meshed cylinder in the other outer chamber. The test mouse was returned to the three-chambered apparatus and allowed to freely explore all three chambers for 5 minutes. The activity was videotaped and tracked with a dedicated video-tracking software. Social interaction, defined as any nose entry into a pre-defined interaction zone around each cylinder, was scored by a blinded experimenter. Since social behavior was unaffected in the *Early postnatal deletion cohort* and in the *Adult deletion cohort*, the *Mouse line control cohort* was not assessed in the three-chambers social interaction test.

Light-Dark (LD) Box Test. The LD box test was carried out as previously described (6). Briefly, mice were placed in the lit compartment facing the wall and observed for 5 minutes. The percentage of time spent in the light, the latency to enter the dark compartment, and the frequency of light-dark transitions were analyzed.

Two-way active avoidance (TWA) test. The TWA test was performed using a multi-conditioning system (TSE systems) as previously described (7, 8). Briefly, the mice received one training session per day for five consecutive days. Each session consisted of 60-sec habituation followed by 25 learning trials. Each trial included a 5-sec 1 kHz 60 dB tone (conditioned stimulus, CS) immediately followed by a 0.4 mA E-shock (unconditioned stimulus, US; cut-off: 5-sec), and an additional 60-second delay. The E-shock ceased either when the mouse moved to the other compartment or after 5-sec, if no movement occurred. If the mouse moved during

CS the E-shock was not delivered. An inter-trial interval of 30 seconds was maintained between trials. A conditioned response (CR) implicated an active transfer to the other chamber upon CS presentation; an unconditioned response (UR) implicated an active transfer in response to the US; a failure consisted of the absence of any transfer to the alternate compartment. Learning performance was calculated as the percentage of CR relative to total responses (CR and UR) for each animal in each trial. Mice repeatedly escaping from the test box during trials were excluded (n=2).

Automated behavioral screening via DeepLabCut

The automated behavioral screening was performed with DeepLabCut (9). Each original video (1280 x 1024 pixels, 25 f/s) was cropped and cut to a final format of 256 x 256 pixel and 5 min duration. DeepLabCut was trained to detect and discriminate between the snout, left ear, right ear, and tail base of the mouse in the video. The data were filtered for likelihoods greater than 0.95. and the center point was calculated and exported. The output data included the following items: batch, trial, and arena; for each trial time and xy coordinates were extracted and ID, genotype, sex, and a "switched" attribute were added. The switched attribute identifies the videos where the left toy was replaced with a new one in the testing of the NORT. The traces (i.e., position of the mouse over time) were visualized as line plot and heatmap and were overlaid on the video. The Euclidean distances to the objects were calculated and added to the table for each position (dl/r: position to left/right object [px]). The speed for consecutive positions was calculated as

pixels/minute; the absolute angle between the movement and the direction to each object was calculated (al/r: angle to left/right toy [degrees]).

The angle values of each mouse position (range: 0-180°) were normalized to values from 1 to 0, with 1 indicating direct approach and interaction with the object, and 0 indicating retreat from the object (al/rn: angle left/right normalized). The distance to the object values were normalized so that they would range from 1 to 0, with 1 indicating a direct approach to the object, and 0 representing the maximum distance from the object (dl/rn: distance left/right normalized). The normalized distances and angles were categorized into equal bins for frequency counting (dn/otn[0-9]: distance new/old object normalized bin #, angn/otn[0-9]: angle new/old object normalized bin #). The difference in frequency between the bins for the new and old object was analyzed for angles and distances (d/ant[0-9] – d/aot[0-9]: distance/angle new toy bin – distance/angle old toy).

Statistical analysis

The analyses were performed with GraphPad Prism software10.0 or with R software, version 4.4.2. Significance level was set to $P < 0.05$ and the data are presented as mean ± SEM. Sample distribution was assessed via Shapiro-Wilk normality test. For normally distributed dataset, two-group comparison was performed via unpaired or paired two-tailed t-test. For non-normally distributed dataset, two-group comparison was performed via Mann–Whitney U (unpaired data) or Wilcoxon test (paired data). Correlation was assessed via Pearson’s or Spearman’s (non-parametric) test. Comparison between more than two groups

were carried out by, two-way or three-way ANOVA, followed by Tukey’s or Sidak post-hoc test. Mixed model design was applied for the statistical comparison of the nodes of Ranvier between groups. The number of biological replicates (n), specific statistical tests, and their P-values are indicated in the figure legends. Behavioral experiments were replicated in two independent batches. Detailed information of the statistical analyses is provided in the Supplementary Table S1.

Supplementary references

1. L. Pillai-Kastoori, A. R. Schutz-Geschwender, J. A. Harford, A systematic approach to quantitative Western blot analysis. *Anal Biochem* **593**, 113608 (2020).

2. J. Schindelin, *et al.*, Fiji: an open-source platform for biological-image analysis. *Nat Methods* **9**, 676–682 (2012).

3. G. Poggi, J. Albiez, C. R. Pryce, Effects of chronic social stress on oligodendrocyte proliferation-maturation and myelin status in prefrontal cortex and amygdala in adult mice. *Neurobiology of Stress* **18**, 100451 (2022).

4. M.-K. Koskinen, *et al.*, Node of Ranvier remodeling in chronic psychosocial stress and anxiety. *Neuropsychopharmacology* **48**, 1532–1540 (2023).

5. J. J. Nadler, *et al.*, Automated apparatus for quantitation of social approach behaviors in mice. *Genes Brain and Behavior* **3**, 303–314 (2004).

6. M. A. Van Der Kooij, *et al.*, Chronic social stress-induced hyperglycemia in mice couples individual stress susceptibility to impaired spatial memory. *Proc. Natl. Acad. Sci. U.S.A.* **115** (2018).

7. K. Braun, *et al.*, Imaging of Functional Brain Circuits during Acquisition and Memory Retrieval in an Aversive Feedback Learning Task: Single Photon Emission Computed Tomography of Regional Cerebral Blood Flow in Freely Behaving Rats. *Brain Sciences* **11**, 659 (2021).

8. A. Mannewitz, *et al.*, Comparing brain activity patterns during spontaneous exploratory and cue-instructed learning using single photon-emission computed tomography (SPECT) imaging of regional cerebral blood flow in freely behaving rats. *Brain Struct Funct* **223**, 2025–2038 (2018).

9. A. Mathis, *et al.*, DeepLabCut: markerless pose estimation of user-defined body parts with deep learning. *Nat Neurosci* **21**, 1281–1289 (2018).

Supplementary figures

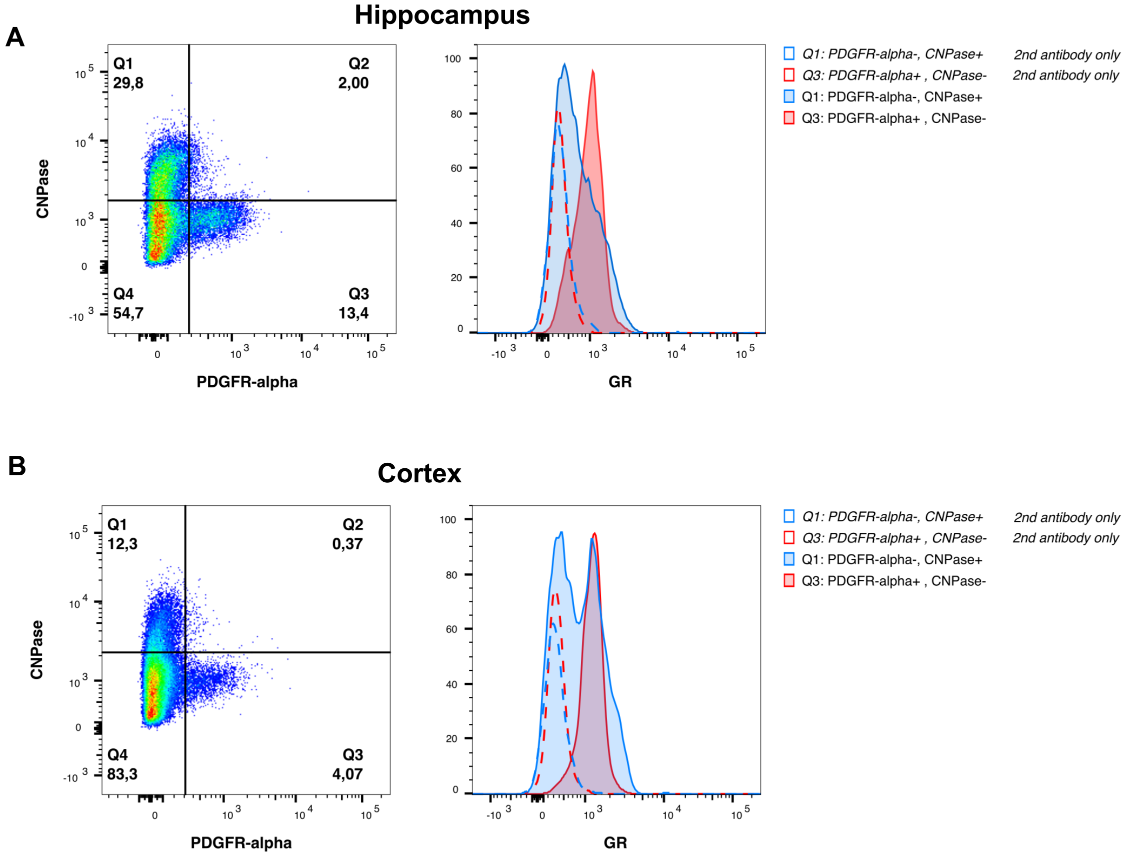

Fig. S1. Glucocorticoid receptor (GR) expression by mature oligodendrocytes and their precursors in the cortex and hippocampus of adult mice. Single-cell suspensions from dissected hippocampi (**A**) and cortex (**B**) were analyzed by flow cytometry. After excluding dead cells, doublets, and CD45+CD11b+ cells, mature oligodendrocytes and precursor cells were identified by expression of CNPase and PDGFR-alpha, respectively (dot-plots, left panel). Each population was then displayed in overlayed histogram plots indicating GR expression (right panel): CNPase+ (blue) and PDGFRA+ (red) populations with (filled) or without (secondary antibody only, dashed) anti-GR antibody.

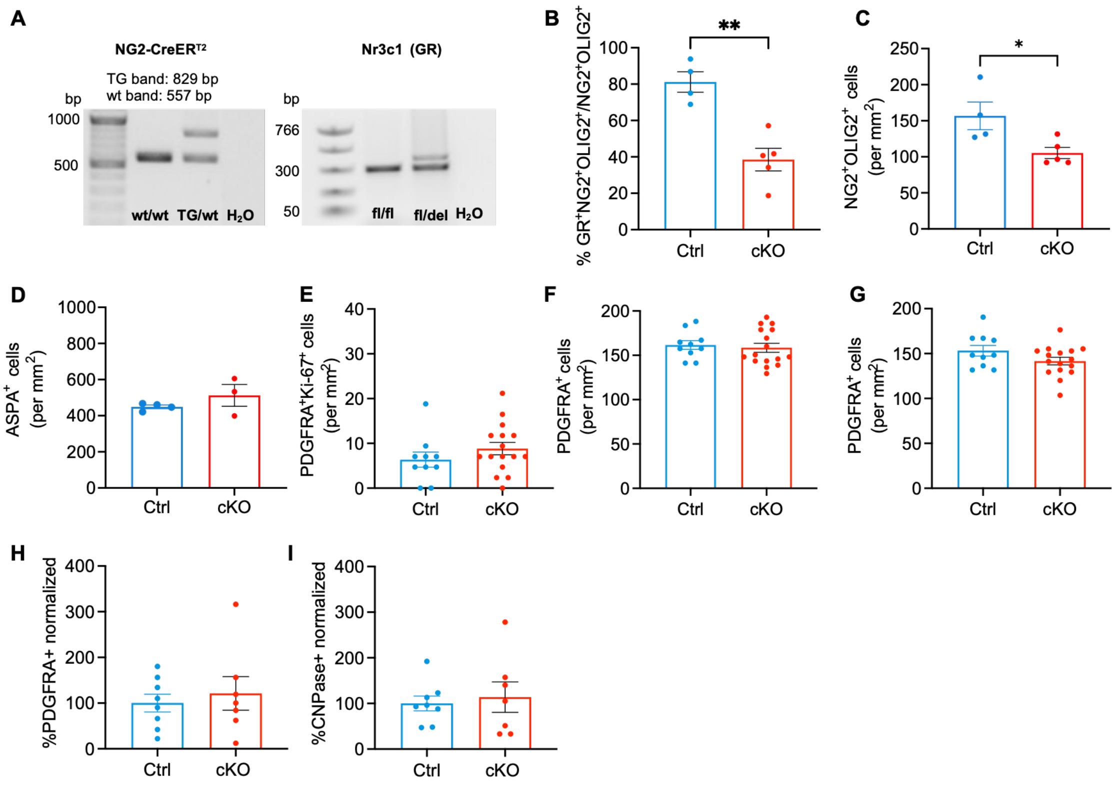

Fig.S2. Generation of NG2-CreERT2; Nr3c1fl/fl mice and quantification of OPCs and oligodendrocyte density in the cortex of adult mice (**A**) PCR genotyping of knock-in mice expressing Cre DNA recombinase variant CreERT2 in the NG2 (Cspg4) locus, and the floxed Nr3c1 (GR) gene. (**B-G**) Quantification of Cre recombinase efficiency and OPCs and myelin marker density in Ctrl and cKO mice. (**B**) Percentage of GR+NG2+ cells the cortex (unpaired t test: $t(7) = 4.956$, $p = 0.0016$; n = 4 Ctrl and n = 5 cKO mice). (**C**) Density of NG2+Olig2+ cells (unpaired t test: $t(7) = 2.718$, $p = 0.0299$; n = 4 Ctrl and n = 5 cKO mice). (**D**) Density of ASPA+ cells in the cortex (unpaired t test with Welch's correction: $p = 0.4041$; n = 4 Ctrl and n = 3 cKO mice). (**E**) Density of PDGFRA+Ki-67+ cells in the cortex (unpaired t test: $p = 0.2725$; n = 10 Ctrl and n = 16 cKO mice). (**F**)

Density of PDGFRA + cells in the hippocampus (unpaired t test: $p = 0.683$), and **(G)** in the cortex (unpaired t test: $p = 0.1626$; n = 10 Ctrl and n = 16 cKO mice). (**H**) Percentage of single PDGFRA + cell population among CD45-CD11b- cells dissected from the cortex (unpaired t test: $p = 0.6042$). (**I**) Percentage of single CNPase+ cell population among CD45-CD11b- cells dissected from the cortex (unpaired t test: $p = 0.7033$; n = 8 Ctrl and n = 7 cKO mice). Data are expressed as the mean ± S.E.M. and each dot represents one animal. *P < 0.05, **P <, 0.01.

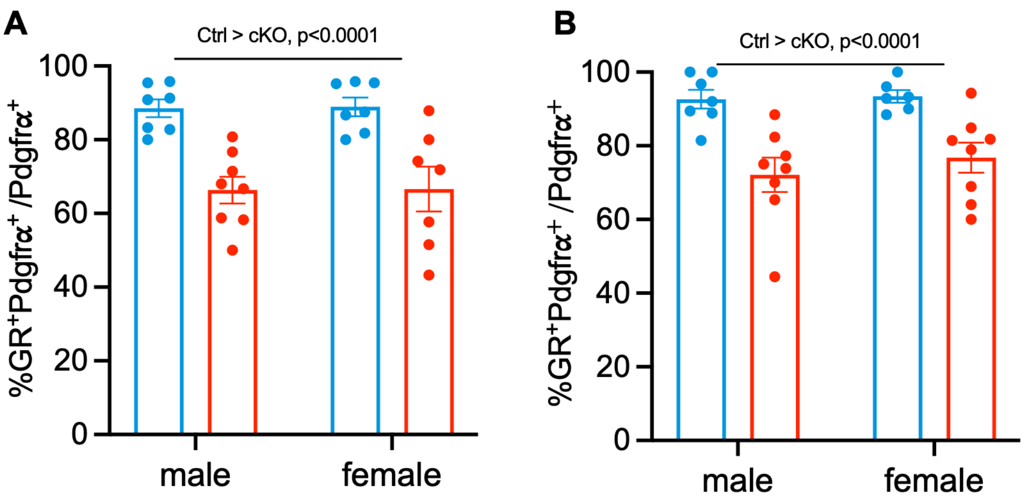

Fig. S3. The loss of GR in OPCs and in mature oligodendrocytes is equally observed in both sexes. (**A**) Percentage of GR+-OPCs with respect to the overall OPC population in the hippocampus (two-way ANOVA, genotype: $F(1, 25) = 31.75$, $p<0.0001$; sex: $F(1, 25) = 0.008369$, $p = 0.9278$; genotype x sex: $F(1, 25) = 0.008369$, $p=0.9888$). (**B**) Percentage of GR+-OPCs with respect to the overall OPC population in the cortex (two-way ANOVA, genotype: $F(1, 25) = 24.58$, $p<0.0001$; sex: $F(1, 25) = 0.5317$, $p = 0.4727$; genotype x sex: $F(1, 25) = 0.2711$, $p=0.6072$). Data are expressed as the mean ± S.E.M. and each dot represents one animal.

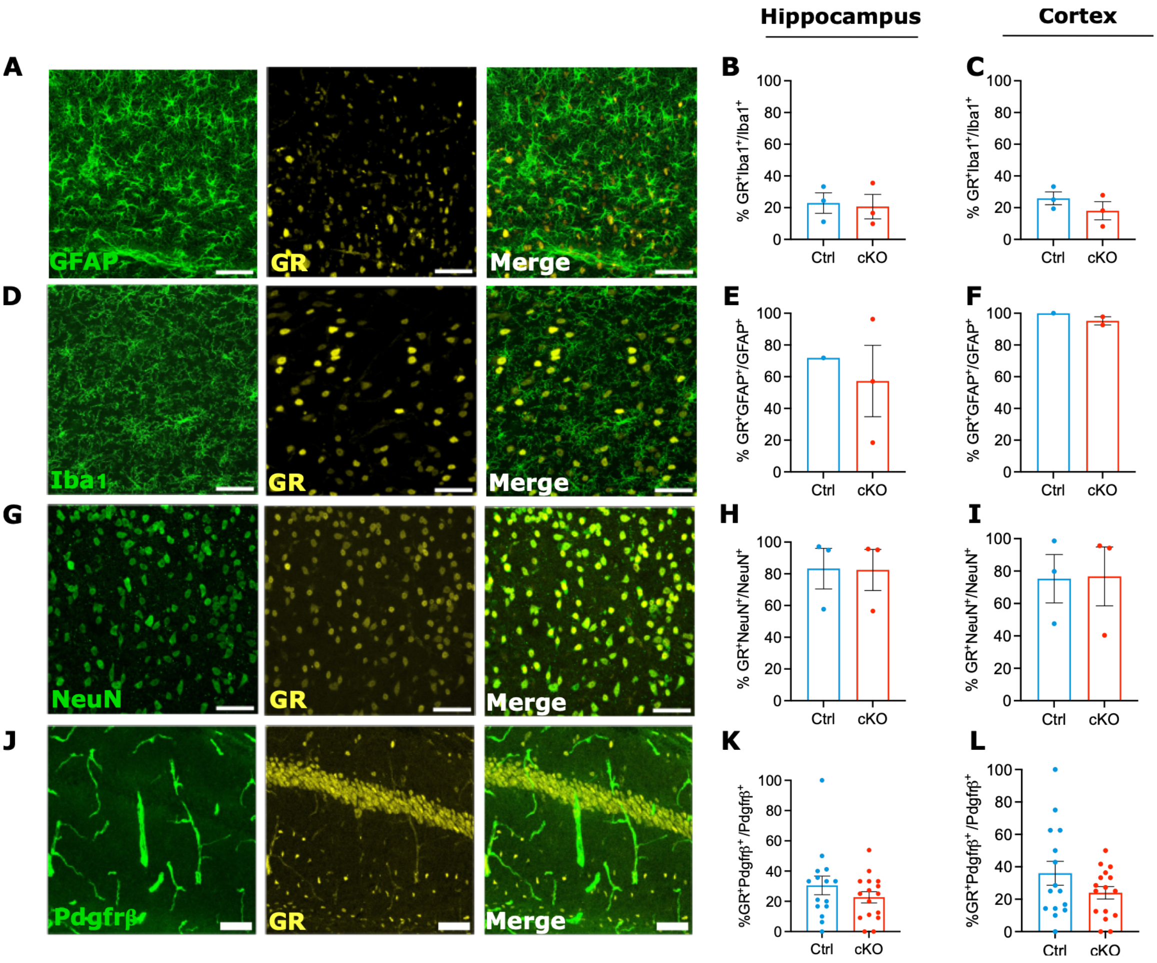

Fig.S4. GR expression in astrocytes, microglia, neurons and pericytes in cKO mice. (**A-C**) Representative confocal images and quantification of the co-expression of GR with astrocytes (GFAP) in hippocampus and cortex, n = 1 Ctrl and n = 3 cKO. **(D-F)** Representative confocal images and quantification of the co-expression of GR with microglia (Iba1) in hippocampus and cortex, n = 3 Ctrl and n = 3 cKO. **(G-I)** Representative confocal images and quantification of the co-expression of GR with neurons (NeuN) in hippocampus and cortex, n = 3 Ctrl and n = 3 cKO. **(J-I)** Representative confocal images and quantification of the co-expression of GR with pericytes (PDGFRB) in hippocampus (Mann-Whitney, U=95.50, p=0.3407) and cortex (Unpaired t test with Welch's correction, t=1.454,

df=21.10, p=0.1606), n = 15 Ctrl and n = 16 cKO. Scale bars = 50 µm. Images acquired in Cornu ammonis 1(CA1) and layer 2/3 of the primary somatosensory cortex, Bregma: -1.79 to -2.53 mm. Data are expressed as the mean ± S.E.M and each dot represents one animal. Brightness and contrast of the micrographs have been adjusted for display purposes.

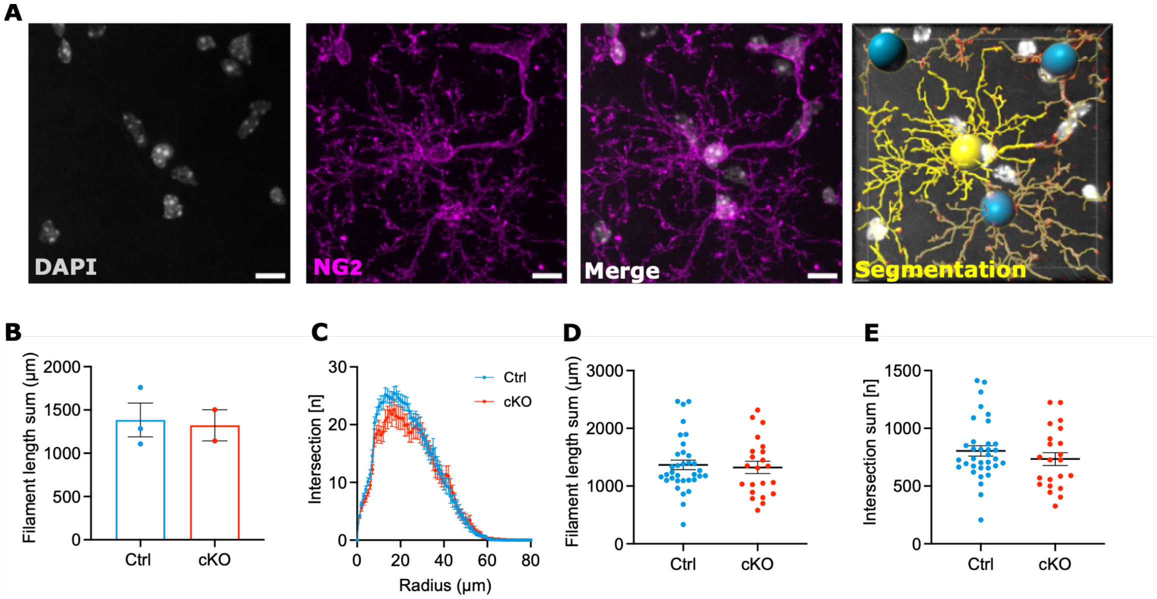

Fig. S5. Morphological characterization of OPCs in cKO mice. (**A**) Representative images of NG2+ cell with segmentation analysis micrograph in hippocampal CA1 subregion. Scale bar = 10 µm. Brightness and contrast have been adjusted for display purposes. (**B**) Mean of filament length sum and (**C**) OPCs branch intersections. (**D**) Filament length sum distribution. (**E**) Intersection sum distribution. Sample size: Ctrl, n=3 (cell, n=34); cKO, n=2 (cell, n=23).

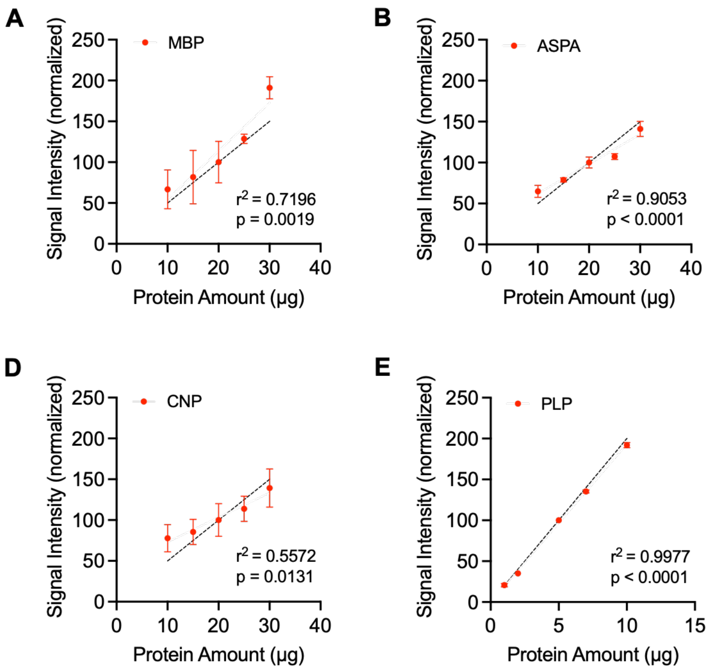

Fig. S6. Evaluation of the linearity in protein-level quantification. The correlation between the amount of loaded protein and the signal intensity is linear within the range utilized to compare Ctrl and cKO mice. Samples from two Ctrl mice. For MBP (**A**), ASPA (**B**), NG2 (**C**), and CNP (**D**), 10, 15, 20, 25, and 30 µg of total protein were loaded. For PLP (**E**), 1, 2, 5, 7, and 10 µg of total protein were loaded. Data points represent the average intensity of all two samples at each amount of total protein (normalized to the signal intensity at 20 µg or 5 µg total protein). The intensity for NG2 deviated slightly from the linear relation, but both signals still showed significant differences with 25% changes in total protein levels. Simple linear regression, error bars represent S.E.M. The dashed line represents the linear regression plot, predicting signal intensities of 50% at 10 µg, 75% at 15 µg, 100% at 20 µg, 125% at 25 µg, and 150% at 30 µg. The measured signal intensity for the synaptic proteins of interest closely aligned with this hypothetical

plot within the range of 20 to 25 µg or 2,5 to 10 µg total protein which was used for the study, confirming the linearity of the protein quantifications (**A-E**). PLP total protein stain for normalization purposes showed a tight linear relationship with the amount of total protein loaded (**E**).

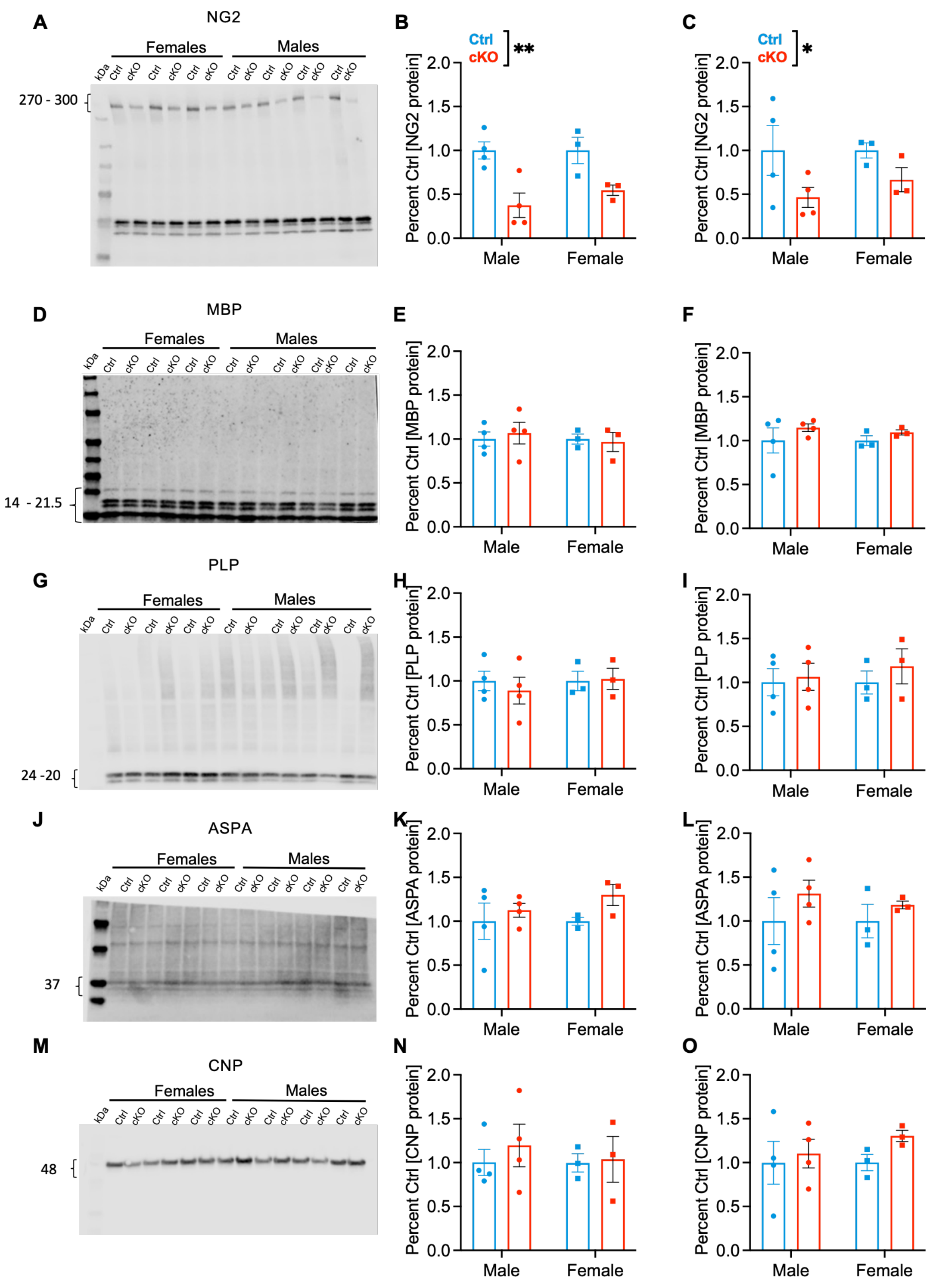

Fig. S7. Quantification of OPCs and myelin markers proteins level in the hippocampus and cortex. Representative western blotting of Hippocampi (HP)

or cortices (CX) lysates stained for oligodendrocyte lineage-related markers (n = 4M/3F Ctrl and n= 4M/3F cKO mice). (**A-C**) **Neural/glial antigen 2 (NG2)** (**A**) Representative blot. (**B**) NG2 protein level in HP (Two-way ANOVA: genotype, $F_{(1, 10)} = 19.76$, $p = 0.0012$; sex and interaction, $p = 0.4953$). (**C**) NG2 protein level in CX (Two-way ANOVA: genotype, $F_{(1,10)} = 5.060$, $p = 0.0482$; sex and interaction, $p \geq 0.6128$). (**D-F**) **Myelin basic** protein**.** (**D**) Representative blot. (**E**) MBP protein level in HP (Two-way ANOVA: $p = 0.6299$. (**F**) MBP protein level in CX (Two-way ANOVA: $p = 0.2279$. (**G-I**) **Proteolipid protein (PLP)**. (**G**) Representative blot. (**H**) PLP protein level in HP (Two-way ANOVA: $p = 0.623$). (**I**) PLP protein level in CX (Two-way ANOVA: $p \geq 0.4687$). (**J-L**) **Aspartoacylase (ASPA)**. (**J**) Representative blot. (**K**) ASPA protein level in HP (Two-way ANOVA: $p \geq 0.1643$). (**L**) ASPA protein level in CX (Two-way ANOVA: $p = 0.2442$). (**M-O**) **2′,3′-cyclic nucleotide 3′-phosphodiesterase, E.C.3.1.4. 37 (CNP).** (**M**) Representative blot. (**N**) CNP protein level in HP (Two-way ANOVA: $p = 0.5839$. (**O**) CNP protein level in CX (Two-way ANOVA: $p = 0.2822$). Data are expressed as the mean ± S.E.M and each dot represents one animal. *$P < 0.05$, **$P < 0.01$.

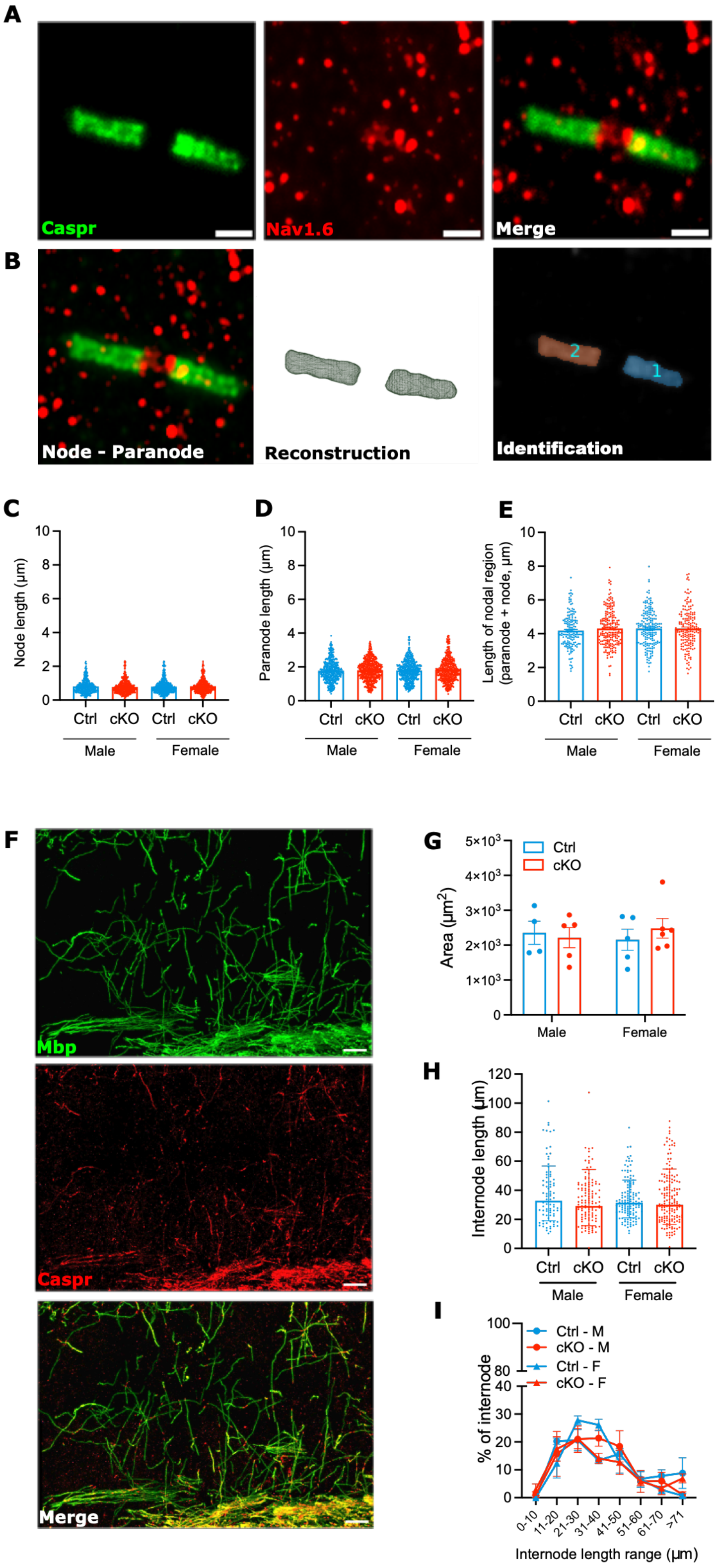
A
Caspr
Nav1.6
Merge
B
Node - Paranode
Reconstruction
Identification
C
Node length (µm)
Ctrl cKO Ctrl cKO
Male Female
D
Paranode length (µm)
E
Length of nodal region (paranode + node, µm)
F
Mbp
Caspr
Merge
G
Ctrl
cKO
Area (µm2)
Male
Female
H
Internode length (µm)
I
Ctrl - M
cKO - M
Ctrl - F
cKO - F
% of internode
Internode length range (µm)

Fig. S8. The length of paranodes, nodes of Ranvier, and internodes is comparable between experimental groups and sexes. (**A**) Representative micrograph of a paranode (Caspr, *green*) and node of Ranvier (Nav1.6, *red*); scale bar = 1 μm. Brightness and contrast have been adjusted for display purposes. (**B**) Representative images of the reconstruction and identification of two paranodes and the related node of Ranvier. (**C**) Node length (Linear mixed model, p>0.05; Ctrl male, n = 390 nodes; cKO male, n = 481 nodes; Ctrl female, n = 465 nodes; cKO female, n = 456 nodes). (**D**) Paranode length (Linear mixed model, p>0.05; Ctrl male, n = 444 nodes; cKO male, n = 584 nodes; Ctrl female, n = 515 nodes; cKO female, n = 517 nodes). (**E**) Length of paranodes and the related node of Ranvier (Linear mixed model, p>0.05; Ctrl male, n = 157 nodes; cKO male, n = 187 nodes; Ctrl female, n = 209 nodes; cKO female, n = 174 nodes). Graph shows mean ± S.E.M, total mice, n= 28 (Ctrl male, n = 6; cKO male, n = 8; ctrl female, n = 7; cKO female, n = 7). (**F**) Representative confocal images, scale bar = 20 μm. (**G**) Myelin content in hippocampal CA1 (Two-Way ANOVA: Sex F (1,16) = 0.01383, p = 0.9078; genotype, F (1, 16) = 0.09833), p = 0.7579; sex x genotype, F (1, 16) = 0.5999, p = 0.4499). (**H**) Internodal length in hippocampal CA1 (Two-Way ANOVA: Sex F (1, 16) = 0.04783, p = 0.8296; genotype, F (1, 16) = 0.5837, p = 0.4560; sex x genotype, F (1, 16) = 0.2305, p = 0.6377). (**I**) Binned internodal length in hippocampal CA1 (Three-way ANOVA: Internode range, F (7, 112) = 19.58, p<0.0001; sex, F (1, 16) = 2.898, P=0.1080; genotype, F (1, 16) = 1.619, P=0.2214; Internode range x sex, F (7, 112) = 0.6832, P=0.6860; Internode range x genotype, F (7, 112) = 0.2093, P = 0.9827; sex x genotype, F (1, 16) = 0.06740,

P=0.7985; Internode range x sex x genotype, F (7, 112) = 2.086, P=0.0507. Data are expressed as the mean ± S.E.M, except for (**H**), where data are expressed as the geometric mean ± S.E.M, *P<0.05, **P < 0.01. Contrast and brightness of the confocal images have been adjusted for display purposes. *Males:* n = 4 Ctrl and n = 5 cKO; *Females*: n = 5 Ctrl and n = 6 cKO.

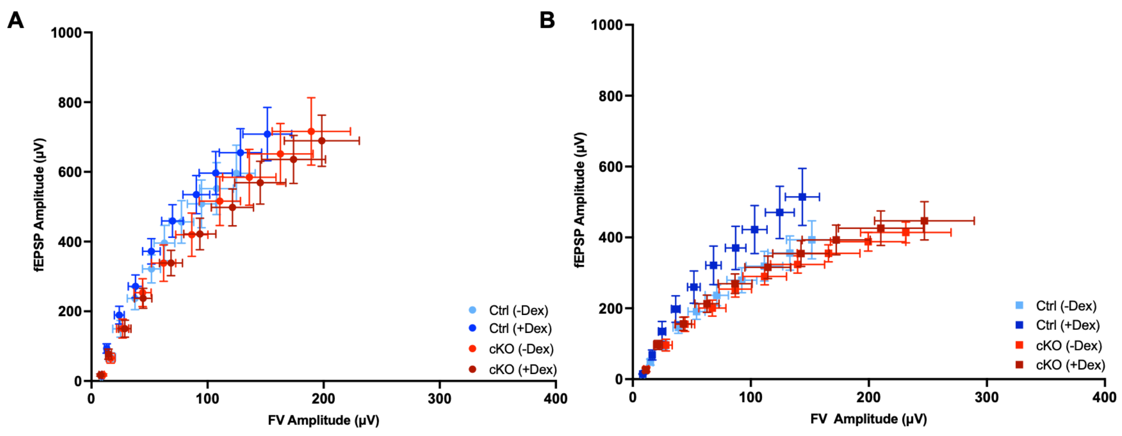

Fig. S9. **Fiber volley**. (**A**) Fiber Volley (FV) Amplitude vs. fEPSP Amplitude in male Ctrl vs. cKO with and without dexamethasone treatment. (**B**) FV Amplitude vs. fEPSP Amplitude in female Ctrl vs. cKO with and without dexamethasone treatment. Graph shows mean ± S.E.M.

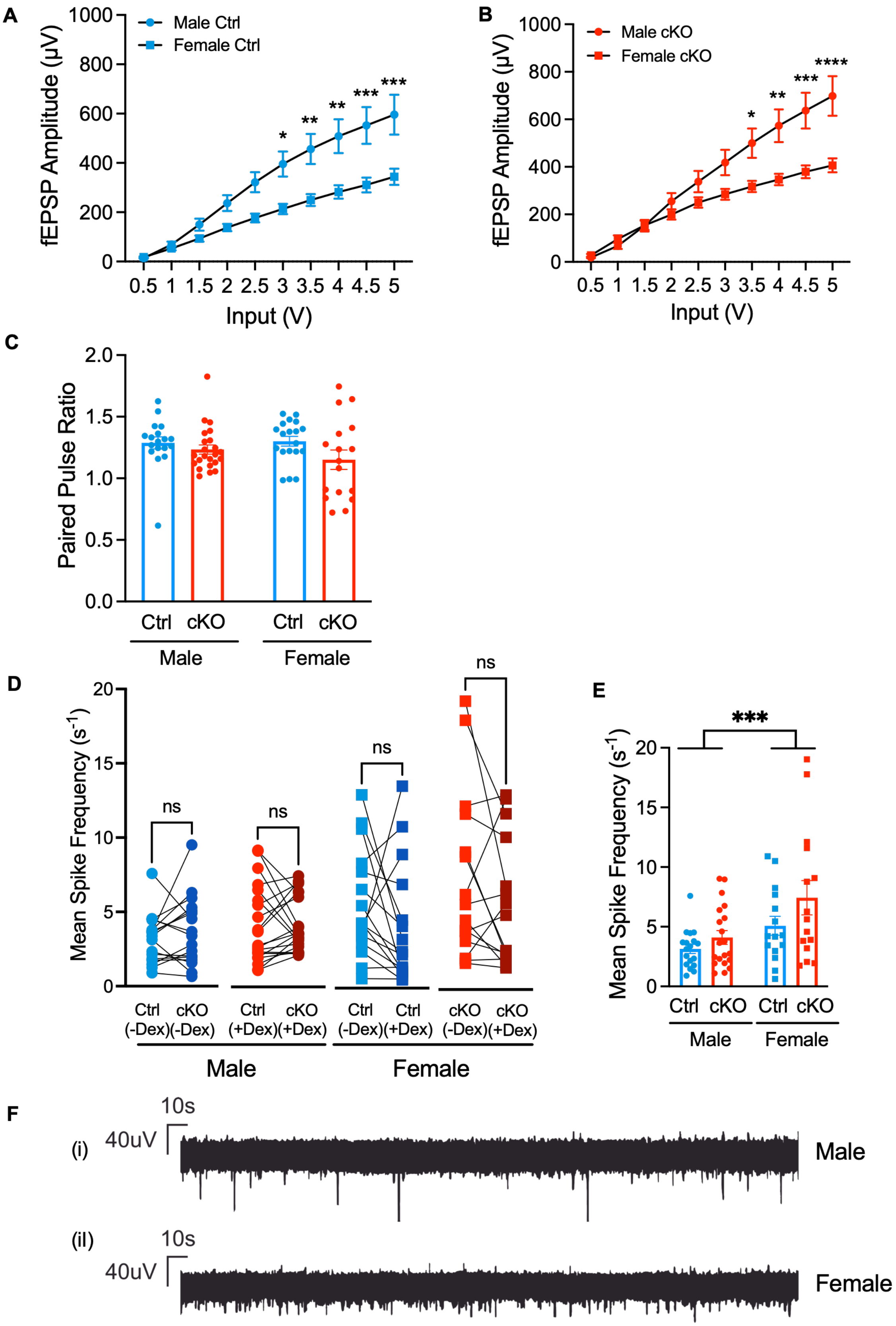

A
Male Ctrl
Female Ctrl
fEPSP Amplitude (µV)
Input (V)
B
Male cKO
Female cKO
fEPSP Amplitude (µV)
Input (V)
C
Paired Pulse Ratio
Ctrl cKO Ctrl cKO
Male Female
D
Mean Spike Frequency (s-1)
ns
Ctrl (-Dex) cKO (-Dex) Ctrl (+Dex) cKO (+Dex) Ctrl (-Dex) Ctrl (+Dex) cKO (-Dex) cKO (+Dex)
Male Female
E
Mean Spike Frequency (s-1)
***
Ctrl cKO Ctrl cKO
Male Female
F
(i) 10s 40uV Male
(il) 10s 40uV Female

Fig. S10. Male mice show higher hippocampal network excitability than female mice under resting conditions and acute stress failed to alter spontaneous network activity of either sex or genotype in the hippocampal CA1 region, but a sex-specific difference was found at the basal status before drug application. (**A**) Evoked field potential in the CA1 regions of males vs. females Ctrl mice. (**B**) Evoked field potential in the CA1 regions of males vs. females cKO mice. (***Ctrl***: Two-Way RM ANOVA: Input x sex, $F(9, 288) = 7.622$, $p < 0.0001$; Šídák's multiple comparisons test: Input 3: M > F, $p = 0.0185$; Input 3.5: M > F, $p = 0.0043$; Input 4: M > F, $p = 0.0012$; Input 4.5: M > F, $p = 0.0004$; Input 5: M > F, $p = 0.0002$; n = 18 male and n = 16 female. ***cKO***: Two-Way RM ANOVA: Input x sex, $F(9, 333) = 9.396$, $p < 0.0001$; Šídák's multiple comparisons test: Input 3.5: M > F, $p = 0.0412$; Input 4: M > F, $p = 0.0039$; Input 4.5: M > F, $p = 0.0006$; Input 5: M > F, $p < 0.0001$; n = 22 male and n = 17 female). (**C**) Baseline paired pulse ratio (Two-way ANOVA: genotype, $F(1, 73) = 3.934$, $p = 0.0511$; sex and interaction, $p \geq 0.3529$). (**D**) Paired recordings within sex and genotype of the mean spike frequency of the hippocampal region before and after the 30-minute wash-in of dexamethasone (Wilcoxon matched-pairs signed rank test or paired t test: $p \geq 0.1297$). (**E**) Spontaneous spiking at basal state. (**F**) Representative trace of a 5-minute recording showing male (i) and female (ii) spiking activity without any drug wash-in. Graph shows mean ± S.E.M. *P < 0.05, **P < 0.01, ***P < 0.001, ****P < 0.001.

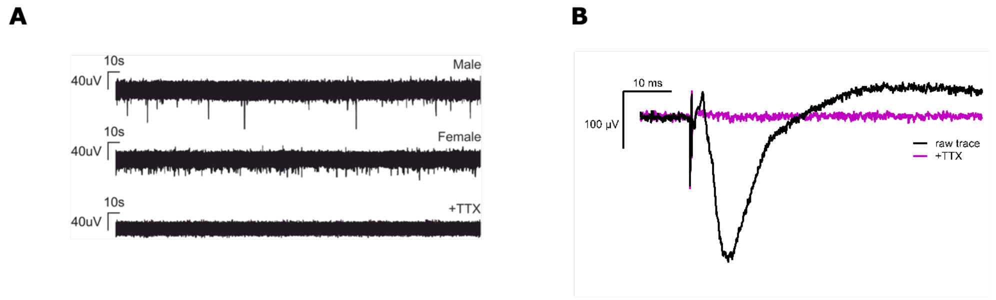

Fig. S11. DNQX/TTX control experimental traces. (**A**) Representative five-minute traces from spontaneous spiking activity recordings of a male and female mouse, and a trace showing the elimination of spiking activity following a 10-minute wash-in of tetrodotoxin (TTX). (**B**) Overlayed evoked fEPSP traces showing the raw signal (black), after a 10-minute wash-in of TTX (pink).

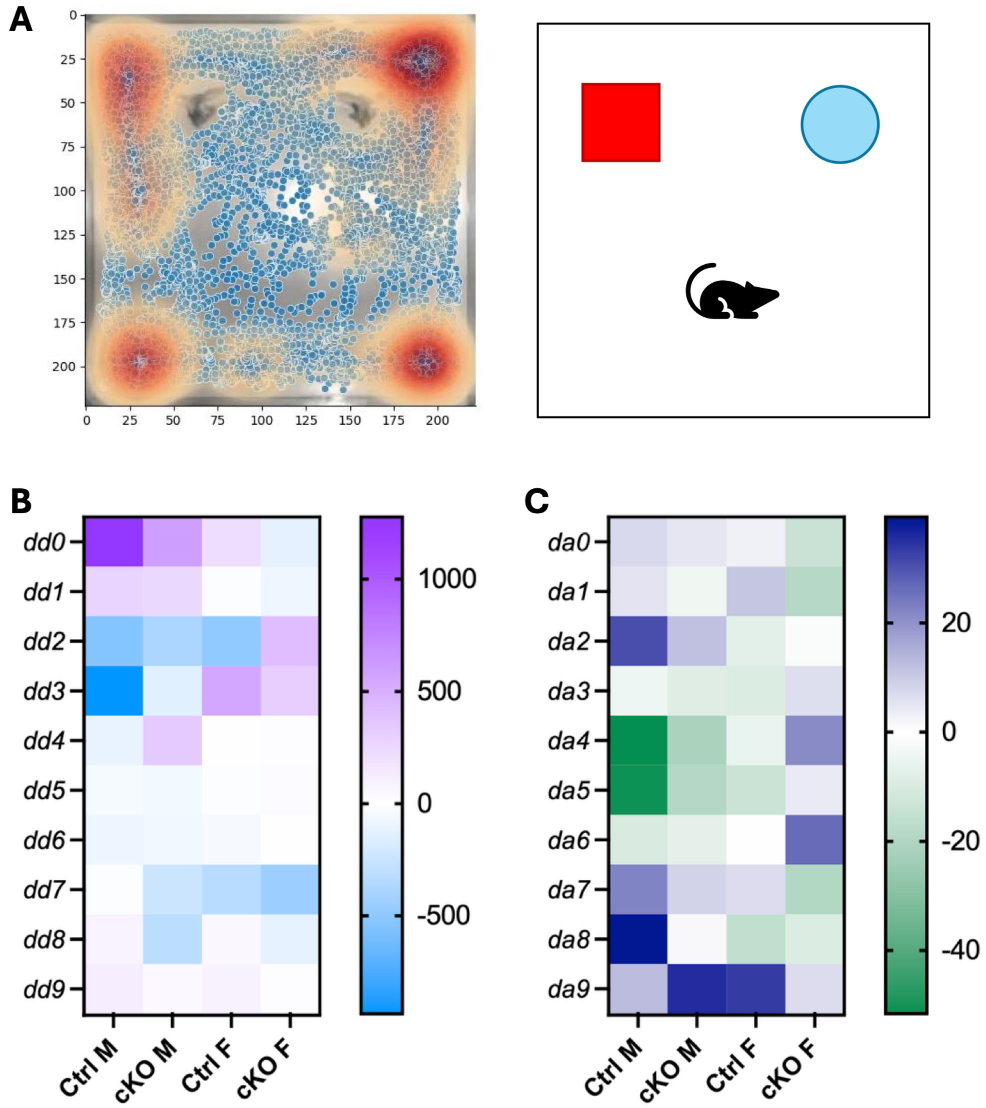

Fig. S12. GR deletion in OPCs does not affect patterns of object exploration during novel object recognition test but reveals a general sex difference in the mouse positioning relative to the objects over time. (**A**) Representative heatmap of arena and object exploration during novel object recognition test (NORT) and representation of NORT setup. (**B**) Heatmap comparing the normalized positioning distance of mice to objects during the testing phase of

NORT (Three-way ANOVA: time $F(2.517, 146.0) = 3.113$, $p = 0.0362$; time x sex $F(9,522)=4.064$, $p<0.0001$; genotype $p>0.9999$). (**c**) Heatmap comparing the normalized turning angle of the mice during the testing phase of NORT (Three-way ANOVA: time x sex $F(9,522)=2.269$, $p=0.0169$; genotype $p>0.9999$).

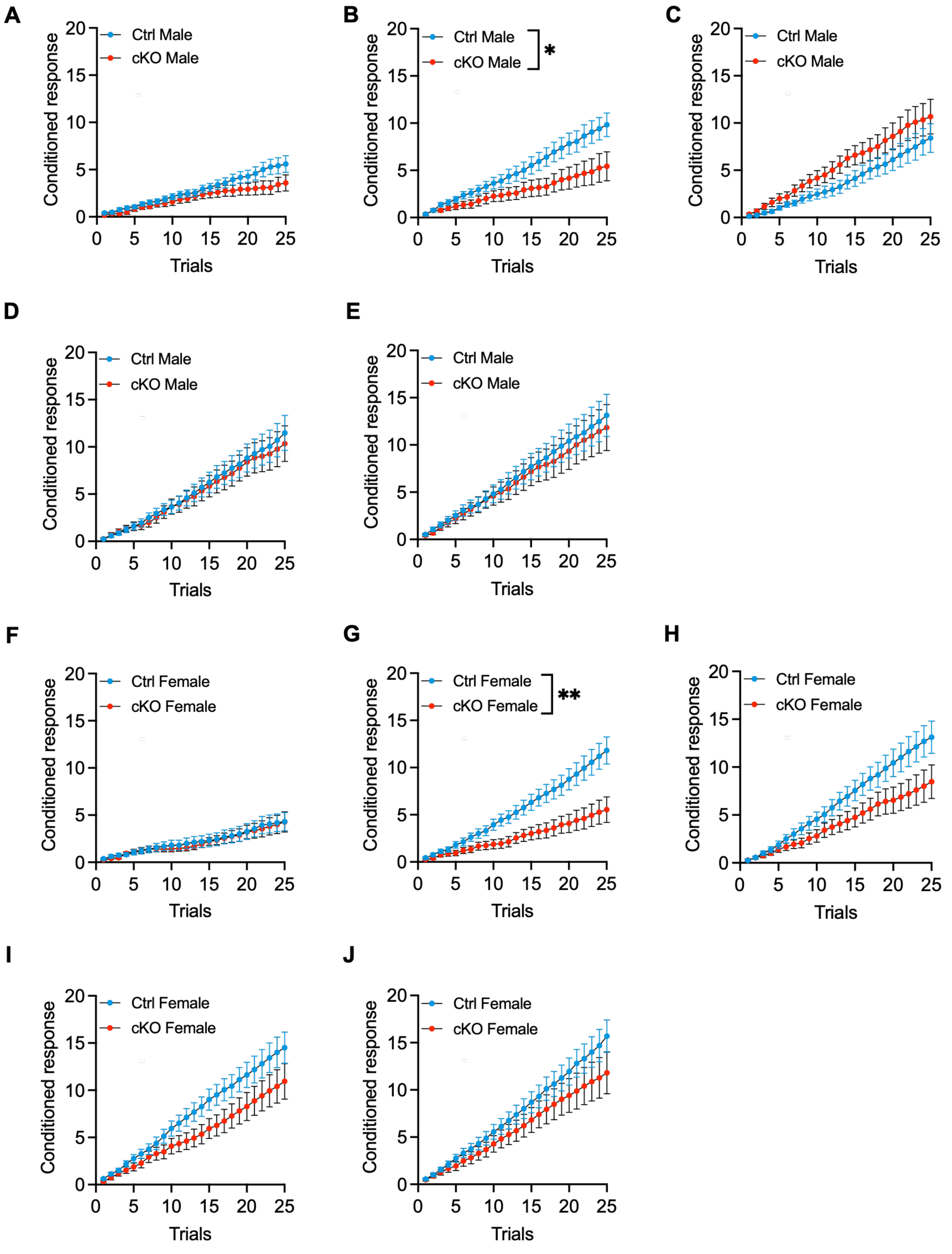
A
B
C
D
E
F
G
H
I
J
Ctrl Male
cKO Male
Ctrl Female
cKO Female
*
**
Conditioned response
Trials
0
5
10
15
20
25

Fig. S13. GR deletion in OPCs affects aversive learning in both adult males and females. Daily conditioned responses (CRs) across 25 trials over five consecutive days in the TWA test. (**A-E**) **Male mice**. (**A**) CR at day 1 (Two-way RM ANOVA: trial x genotype $F(1.351, 36.47) = 35.34$, $p = 0.0027$). (**B**) CR at day 2 (Two-way RM ANOVA: trial x genotype $F(24, 648) = 4.590$, $p < 0.0001$). (**C**) CR at day 3 (Two-way RM ANOVA, trial x genotype, $F(24, 648) = 0.4884$, $p < 0.0001$). (**D**) CR at day 4 (Two-way RM ANOVA: trial $F(1.108, 29.92) = 60.78$, $p < 0.0001$; genotype and interaction $p \geq 0.79279$). (**E**) CR at day 5 (Two-way RM ANOVA: trial $F(24, 648) = 54.07$, $p < 0.0001$; genotype and interaction, $p \geq 0.7426$). **(F-J)** **Female mice**. (**F**) CR at day 1 (Two-way RM ANOVA: trial $F(24, 696) = 21.03$, $p < 0.0001$; genotype and interaction, $p \geq 0.06781$). (**G**) CR at day 2 (Two-way RM ANOVA: trial x genotype $F(24, 696) = 9.818$, $p < 0.0001$). (**H**) CR at day 3 (Two-way RM ANOVA: trial x genotype $F(24, 696) = 3.985$, $p < 0.0001$) . (**I**) CR at day 4 (Two-way RM ANOVA: trial x genotype $F(24, 696) = 2.516$, $p < 0.0001$). (**J**) CR at day 5 (Two-way RM ANOVA: trial $F(1.096, 31.79) = 90.33$, $p < 0.0001$; genotype and interaction, $p \geq 0.0727$). Data are expressed as the mean ± S.E.M, *$P<0.05$, **$P < 0.01$, n = 17M/16F Ctrl and n = 12M/15F cKO.

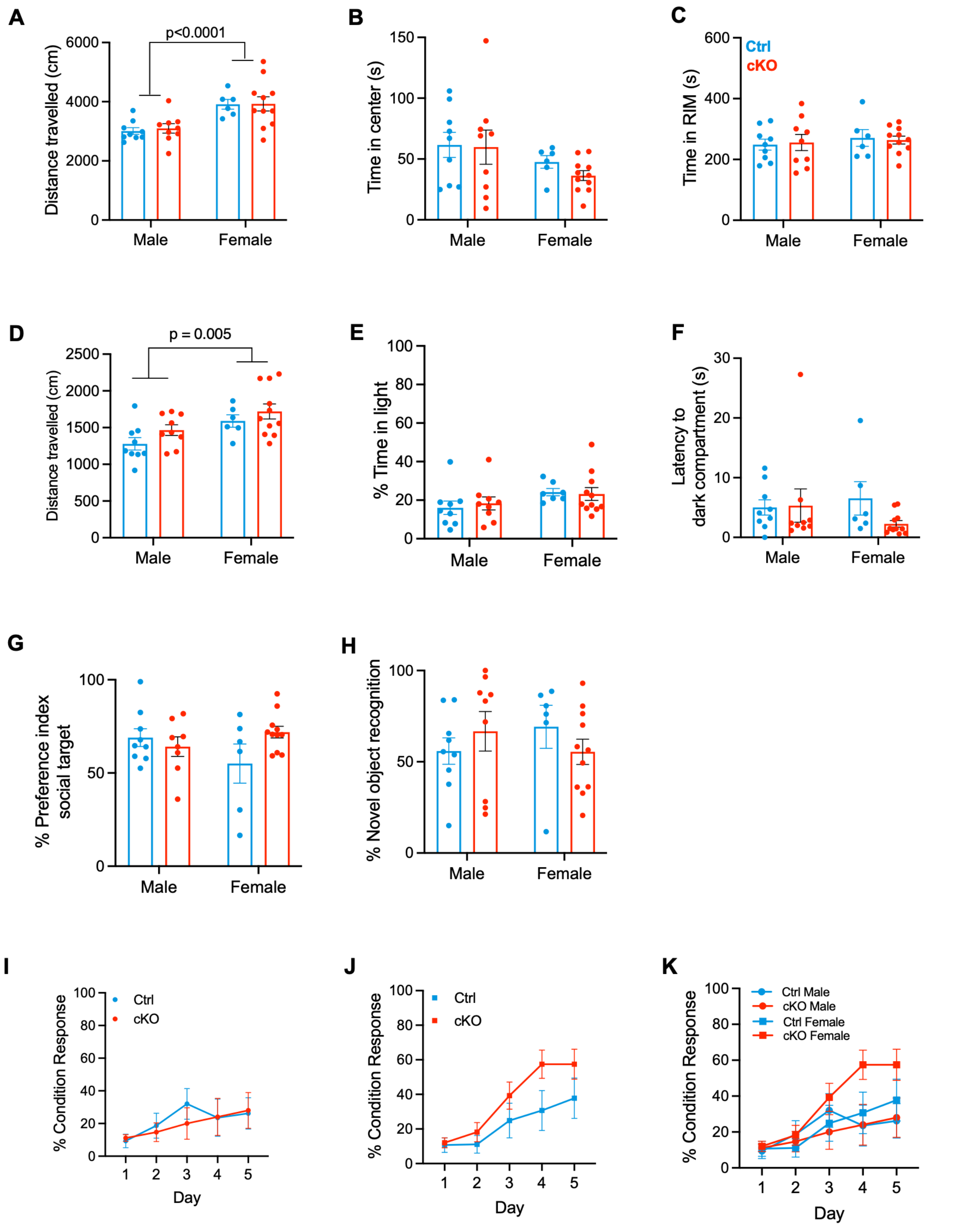

Fig. S14. Deletion of GR in OPC in adulthood does not affect locomotion, cognitive performances, and anxiety-relevant behaviors. (**A-C**) Open field test

(OFT). (**A**) Distance travelled (two-way ANOVA, genotype, F (1, 31) = 0.07048, p=0.7924; sex, F (1, 31) = 19.53, p=0.00001; genotype x sex, F (1, 31) = 0.03289, p=0.8573). (**B**) Time in the center (two-way ANOVA, genotype, F (1, 31) = 0.4649, p=0.5004; sex, F (1, 31) = 3.687, p=0.0641; genotype x sex, F (1, 31) = 0.2312, p=0.6340). (**C**) Time in RIM (two-way ANOVA, genotype, F (1, 31) = 4.474e-007, p=0.9995; sex, F (1, 31) = 0.5076, p=0.4815; genotype x sex, F (1, 31) = 0.1065, p=0.7464). (**D-F**) Light-dark Box Test (LDBT). (**D**) Distance travelled (two-way ANOVA, genotype, F (1, 31) = 2.890, p=0.0991; sex, F (1, 31) = 9.151, p=0.005; genotype x sex, F (1, 31) = 0.09117, p=0.7647). (**E**) Percentage of time in the light compartment (two-way ANOVA, genotype, F (1, 32) = 0.04077, p=0.8413; sex, F (1, 32) = 3.895, p=0.0571; genotype x sex, F (1, 32) = 0.2490, p=0.6212). (**F**) Latency to the dark compartment (two-way ANOVA, genotype, F (1, 31) = 1.065, p=0.31; sex, F (1, 31) = 0.16, p=0.6919; genotype x sex, F (1, 31) = 1.438, p=0.2395). (**G**) Sociability/ Social interaction (two-way ANOVA, genotype, F (1, 30) = 1.158, p=0.2905; sex, F (1, 30) = 0.3074, p=0.5834; genotype x sex, F (1, 30) = 3.773, p=0.0615). (**H**) Percentage of time spent with a novel object (two-way ANOVA, genotype, F (1, 31) = 0.02386, p=0.8782; sex, F (1, 31) = 0.01255, p=0.9115; genotype x sex, F (1, 31) = 1.807, p=0.1886). (**I-K**) Two-way active avoidance. (**I**) Percentage of conditioned response over 5 days in Ctrl vs. cKO males (two-way RM ANOVA, genotype, F (1, 16) = 0.05503, p=0.8175; time, F (2.005, 32.08) = 3.425, p=0.0447; genotype x time, F (2.005, 32.08) = 0.5761, p=0.5682). (**J**) Percentage of conditioned response over 5 days in Ctrl vs. cKO females (two-way RM ANOVA, genotype, F (1, 18) = 2.330, p=0.1443; time, F

(1.980, 35.63) = 19.79, p<0.0001; genotype x time, F (1.980, 35.63) = 1.824, p=0.1765). (**K**) Percentage of conditioned response over 5 days in Ctrl vs. cKO in males vs. females (three-way RM ANOVA, genotype, F (1, 34) = 0.7066, p=0.4065; sex, F (1, 34) = 1.821, p=0.1861; time, F (2.101, 71.44) = 18.65, p<0.0001; time x sex, F (2.101, 71.44) = 3.918, p=0.0226; time x genotype, F (2.101, 71.44) = 1.238, p=0.2971; sex x genotype, F (1, 34) = 1.423, p=0.2411; time x sex x genotype, F (2.101, 71.44) = 1.112; p=0.3367). Data are expressed as the mean ± S.E.M, *P<0.05, **P < 0.01, n = 9M/6F Ctrl and n = 9M/11F cKO.

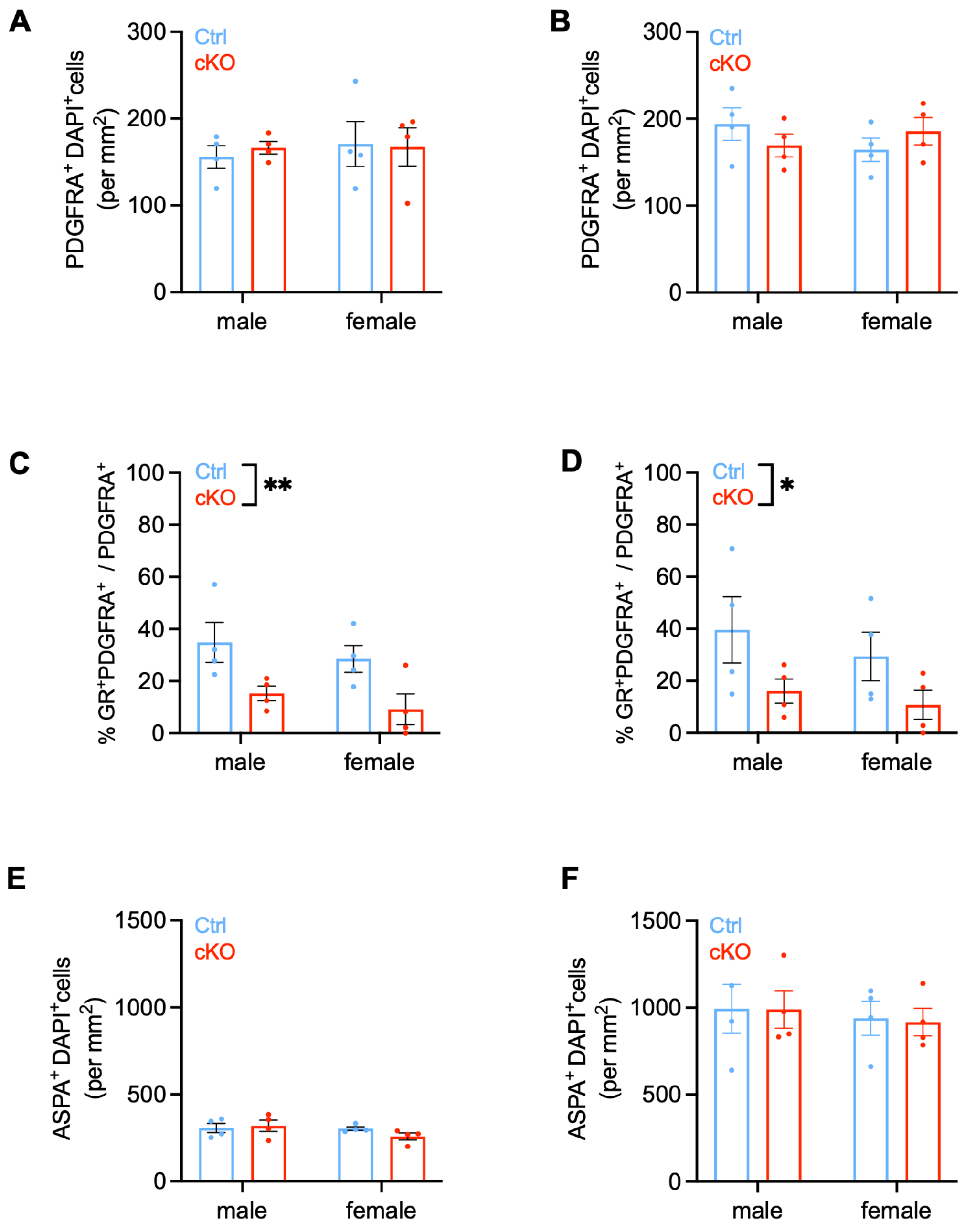

Fig. S15. Deletion of GR in OPC in adulthood is comparably efficient in both sexes. (**A**) OPC density in hippocampus (Two-way ANOVA, genotype, $F(1, 12) = 0.03999$, $p=0.8448$; sex, $F(1, 12) = 0.1837$, $p=0.6758$; genotype x sex, $F(1, 12) = 0.1381$, $p=0.7167$). (**B**) OPC density in cortex (Two-way ANOVA, genotype, F (1,

12) = 0.01109, p=0.9179; sex, F (1, 12) = 0.1854, p=0.6744; genotype x sex, F (1, 12) = 2.239, p=0.1604). (**C**) Percentage of GR+ OPCs in CA1 of the hippocampus (Two-way ANOVA, genotype, F (1, 12) = 11.89, p=0.0048; sex, F (1, 12) = 1.195, p=0.2958; genotype x sex, F (1, 12) = 0.0009083, p=0.9765). (**D**) Percentage of GR+ OPCs in the cortex (Two-way ANOVA, genotype, F (1, 12) = 5.891, p=0.0319; sex, F (1, 12) = 0.8020, p=0.3881; genotype x sex, F (1, 12) = 0.08102, p=0.7808. (**E**) OL density in hippocampus (Two-way ANOVA, genotype, F (1, 12) = 0.4844, p=0.4997; sex, F (1, 12) = 1.877, p=0.1958; genotype x sex, F (1, 12) = 1.410, p=0.2580). (**F**) OL density in cortex (Two-way ANOVA, genotype, F (1, 12) = 0.01408, p=0.9075; sex, F (1, 12) = 0.3486, p=0.5658; genotype x sex, F (1, 12) = 0.006014, p=0.9395).

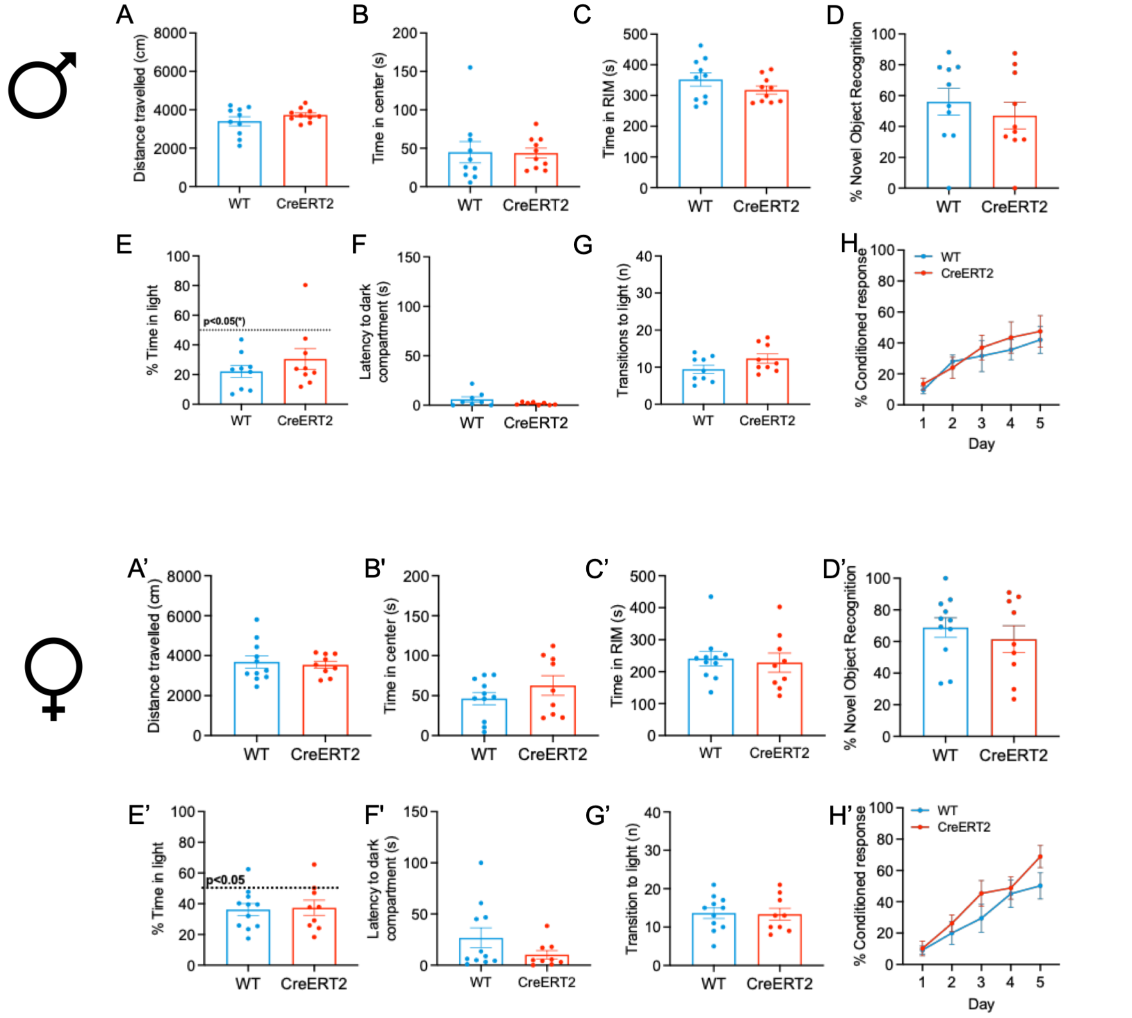

Fig. S16. Lack of an NG2 allele does not alter behavioral readouts, which are instead affected by the loss of GR in NG2-glia in early life. [GP1] (**A-H**) **Behavioral readouts in male mice**. (**A-C**) *Open field test:* (**A**) Locomotion (unpaired t test with Welch's correction: t = 1.247, df = 12.73, p = 0.2347). (**B**) Time spent in the centre of the arena (Mann-Whitney test: U=40.50, p = 0.4935). (**C**) Time spent in the rim of the arena (unpaired t test: t = 1.359, df = 18, p = 0.1909). (**D**) *Novel Object recognition test*: Percentage of time spent in the exploration of

the new object normalised to the overall exploration time (unpaired t test: t = 0.7386, df = 18, p = 0.4696). (**E-G**) *Light-and-dark box test*: (**E**) Percentage of time spent in the illuminated compartment (Mann-Whitney test: U=33, p = 0.5457). (**F**) Latency to access the dark compartment (Mann-Whitney test: U=23, p = 0.3667). (**G**) Number of transitions to the illuminated compartment (unpaired t test: t = 1.732, df = 16, p = 0.1024). (**H**) *Two-way active avoidance*: Percentage of conditioned response over 5 consecutive days (Two-Way RM ANOVA: Day F (2.827, 45.23) = 14.48, p < 0.0001; genotype F (1, 16) = 0.1835, p 0.6741; day x genotype F (2.827, 45.23) = 0.4450, p = 0.7107). (**A'-H'**) **Behavioral readouts in female mice**. (**A'-C'**) *Open field test:* (**A'**) Locomotion (Unpaired t test: t = 0.3743, df=18, p = 0.7126). (**B'**) Time spent in the centre of the arena (Unpaired t test: t = 1.166, df=18, p = 0.2589). (**C'**) Time spent in the rim of the arena (Mann-Whitney test: U = 40, p = 0.5027). (**D'**) Percentage of time spent in the exploration of the new object normalised to the overall exploration time (Unpaired t test: t = 0.7117, df=18, p = 0.4858). (**E'-G'**) *Light-and-dark box test*: (**E'**) Percentage of time spent in the illuminated compartment (Unpaired t test: t = 0.1793, df=18, p = 0.8597). (**F'**) Latency to access the dark compartment (Mann-Whitney test: U =32, p = 0.2014. (**G'**) Number of transitions to the illuminated compartment (Unpaired t test: t = 0.1459, df=18, p = 0.8856). (**H'**) *Two-way active avoidance*: Percentage of conditioned response over 5 consecutive days (Two-Way RM ANOVA: Day F (2.828, 50.90) = 34.70, p < 0.0001; genotype F (1, 18) = 1.171, p 0.2934; day x genotype F (2.828, 50.90) = 1.354, p = 0.2678). Data are expressed as the mean

± S.E.M, *P<0.05, **P < 0.01, *Males:* n = 8-10 WT and n = 8-10 CreERT2; *Females*: n = 11 WT and n = 9 CreERT2.

Supplementary Table S1. Comprehensive overview of the applied statistical models. The employed statistical models, the related output and the sample characteristics are specified for each single graph reported in every figure panel. The reference to the relevant chart and the related dataset identity are reported in the columns, labelled as "Figure" and "Data ID".

Statistics: Figure 1

Figure	Data ID	Method	Region	Sample Size	Sex	Normally distributed (Shapiro-Wilk test)?	Test	U, t or F values	p value
Fig. 1D	Hippo % GR+NG2+OLIG2/NG2+OLIG2	Histology	Hippocampus	Ctrl, n=4<br>cKO, n=5	M/F	NO	Mann Whitney test (2-tailed)	U = 0	**0.0159**
Fig. 1F	Hippo NG2+OLIG2+ cells mm2	Histology	Hippocampus	Ctrl, n=4<br>cKO, n=5	M/F	YES	Unpaied t test (2-tailed)	t=1.070, df=7	0.3202
Fig. 1H	Hippo ASPA+ cells mm2	Histology	Hippocampus	Ctrl, n=4<br>cKO, n=3	M/F	YES	Unpaired t test with Welch's correction (2-tailed)	t=4.038, df=3.129	0.0252
Fig. 1J	Hippo PDGFRalpha/Ki67/DAPI cells mm2	Histology	Hippocampus	Ctrl, n=10<br>cKO, n=16	M/F	YES	Unpaied t test (2-tailed)	t=0.6604, df=24	0.5153
Fig. 1K	% PDGFRA+ Hp	FACS	Hippocampus	Ctrl, n=8<br>cKO, n=7	M/F	YES	Unpaired t test with Welch's correction (2-tailed)	t=2.087, df=7.432	0.073
Fig. 1L	% CNPase+ Hp	FACS	Hippocampus	Ctrl, n=8<br>cKO, n=7*	M/F	YES	Unpaied t test (2-tailed)	t=2.384, df=13	0.0331

Statistic: Figure 2

Figure	Data ID	Method	Region	Sample Size	Sex	Normally distributed (Shapiro-Wilk test)?	Test	Factor	F, df	p value	Post-hoc test
Fig.2B	g-ratio vs. Axon diameter	Transmission Electron Microscopy	Hippocampus (including Schaffer collateral and CA1)	Ctrl, n= 5<br>cKO , n =5	M						
Fig.2C	g-ratio vs. Axon diameter	Transmission Electron Microscopy	Hippocampus (including Schaffer collateral and CA1)	Ctrl, n= 5<br>cKO , n =5	F						
Fig.2D	g-ratio	Transmission Electron Microscopy	Hippocampus (including Schaffer collateral and CA1)	Ctrl - M, n=5<br>Ctrl - F, n = 5<br>cKO - M, n =5<br>cKO - F, n = 5	M/F	Assumed	Two-way ANOVA	Interaction	F (1, 16) = 2.760	P=0.1161	
								Sex	F (1, 16) = 5.878	**P=0.0275**	
								Genotype	F (1, 16) = 1.559	P=0.2298	
Fig.2E	Axon diamter	Transmission Electron Microscopy	Hippocampus (including Schaffer collateral and CA1)	Ctrl - M, n=5<br>Ctrl - F, n = 5<br>cKO - M, n =5<br>cKO - F, n = 5	M/F	Assumed	Two-way ANOVA	Interaction	F (1, 16) = 6.245	**P=0.0237**	n.s. | Tukey's multiple comparisons test
								Sex	F (1, 16) = 1.131	P=0.3034	
								Genotype	F (1, 16) = 1.520	P=0.2354	
Fig.2F	Density of myelinated axons	Transmission Electron Microscopy	Hippocampus (including Schaffer collateral and CA1)	Ctrl - M, n=5<br>Ctrl - F, n = 5<br>cKO - M, n =5<br>cKO - F, n = 5	M/F	Assumed	Two-way ANOVA	Interaction	F (1, 16) = 0.001840	P=0.9663	
								Sex	F (1, 16) = 1.028	P=0.3258	
								Genotype	F (1, 16) = 7.468	**P=0.0147**	

Statistic: Figure 3

Figure	Data ID	Method	Region	Sample Size	Sex	Normally distributed (Shapiro-Wilk test)?	Test	Factor	F, df	p value	Post-hoc test
Fig.3C	Male: WT, KO, -Dex, +Dex	MEA recording	Hippocampus	WT(-Dex), n=18<br>cKO (-Dex), n=22<br>WT (+Dex), n=18<br>KO (+Dex), n= 22	M	Assumed	Three-way RM ANOVA	Input	F (9.000, 342.0) = 166.0	**P<0.0001**	
								Drug	F (0.1438, 5.465) = 0.7468	P=0.3929	
								Genotype	F (1, 38) = 0.007435	P=0.9317	
								Input x Drug	F (1.241, 47.15) = 0.3418	P=0.9604	
								Input x Genotype	F (9, 342) = 0.1920	P=0.9949	
								Drug x Genotype	F (1, 38) = 0.9950	P=0.3248	
								Input x Drug x Genotype	F (9, 342) = 0.9050	P=0.5209	
Fig.3D	Fem: WT, KO, -Dex, +Dex	MEA recording	Hippocampus	WT(-Dex), n=17<br>cKO (-Dex), n=17<br>WT (+Dex), n=16<br>KO (+Dex), 17	F	Assumed	Mixed-effects model (REML)	input	F (9.000, 288.0) = 156.1	**<0.0001**	
								Drug	F (0.1406, 4.498) = 5.752	0.0643	
								Genotype	F (1, 32) = 0.2774	0.6021	
								input x Drug	F (1.162, 35.88) = 9.631	**0.0025**	
								input x Genotype	F (9.000, 288.0) = 0.7044	0.7048	
								Drug x Genotype	F (0.1406, 4.498) = 2.577	0.1045	
								input x Drug x Genotype	F (1.162, 35.88) = 3.622	**0.0593**	
Fig.3D	Fem: WT (-Dex, +Dex)	MEA recording	Hippocampus	WT(-Dex), n=17<br>WT (+Dex), n=16	F	Assumed	Mixed-effects model (REML)	Input	F (1.120, 17.92) = 115.6	**<0.0001**	
								Drug	F (1.000, 16.00) = 5.110	**0.0381**	
								Input x Drug	F (1.128, 16.80) = 5.702	**0.0258**	Paired t test as post-hoc comparison:<br>- Fem: input 3.5: (-Dex)<(+Dex) , p=0.0406<br>- Fem: input 4: (-Dex)<(+Dex) , p=0.0329<br>- Fem: input 4.5: (-Dex)<(+Dex) ,

											p=0.0249<br>- Fem: input 5: (-Dex)<(+Dex) ,<br>p=0.0238
Fig.3D	Fem: cKO (-Dex, +Dex)	MEA recording	Hippocampus	cKO (-Dex), n=17<br>cKO (+Dex), 17	F	Assumed	Two-way RM ANOVA	Input	F (1.402, 22.43) = 90.15	**<0.0001**	
								Drug	F (1.000, 16.00) = 0.6879	0.4191	
								Input x Drug	F (1.267, 20.27) = 1.405	0.2581	
Fig.3E	Male vs. Female + Vehicle vs. Dex Control	MEA recording	Hippocampus	Male<br>WT(-Dex), n=18<br>WT (+Dex), n=18<br>Female<br>WT(-Dex), n=17<br>WT (+Dex), n=16	M/F	Assumed	Three-way RM ANOVA	Input	F (9, 279) = 103.3	P<0.0001	
								Sex	F (1, 31) = 0.1206	P=0.7307	
								Drug	F (1, 31) = 0.2678	P=0.6085	
								Input x Sex	F (9, 279) = 0.1992	P=0.9941	
								Input x Drug	F (9, 279) = 0.9668	P=0.4678	
								Sex x Drug	F (1, 31) = 2.275	P=0.1416	
								Input x Sex x Drug	F (9, 279) = 3.562	P=0.0003	
Fig.3F	Male vs. Female + Vehicle vs. Dex cKO	MEA recording	Hippocampus	Male<br>cKO (-Dex), n=22<br>KO (+Dex), n= 22<br>Female<br>cKO (-Dex), n=17<br>KO (+Dex), 17"	M/F	Assumed	Three-way RM ANOVA	Input	F (9, 288) = 76.00	P<0.0001	
								Sex	F (1, 32) = 0.9674	P=0.3327	
								Drug	F (1, 32) = 0.4098	P=0.5267	
								Input x Sex	F (9, 288) = 0.4053	P=0.9319	
								Input x Drug	F (9, 288) = 1.067	P=0.3872	
								Sex x Drug	F (1, 32) = 0.06509	P=0.8003	
								Input x Sex x Drug	F (9, 288) = 0.1460	P=0.9982	
Fig. 3H	male WT before vs after Dex	MEA recording	Hippocampus	Ctrl (-Dex), n =18<br>Ctrl (+Dex), n=18	M	NO	Wilcoxon matched-pairs signed rank test		W = 89.00	0.0539	
Fig. 3H	male KO before vs after Dex	MEA recording	Hippocampus	cKO (-Dex), n =23<br>cKO (+Dex), n= 23	M	NO	Wilcoxon matched-pairs signed rank test		W = 46.00	0.5009	

Fig. 3H	fem WT before vs after Dex	MEA recording	Hippocampus	Ctrl (-Dex), n =19<br>Ctrl (+Dex), n=19	F	YES	Paired t test	t=2.247, df=18	**0.0374**
Fig. 3H	fem KO before vs after Dex	MEA recording	Hippocampus	cKO (-Dex), n =17<br>cKO (+Dex), n=17	F	NO	Wilcoxon matched-pairs signed rank test	W = - 31.00	0.4874

Statistic: Figure 4

Figure	Data ID	Method	Region	Sample Size	Sex	Normally distributed (Shapiro-Wilk test)?	Test	F, df	p value	Post-hoc test
Fig. 4C	LTP female + DEX	LTP recording	Hippocampus	Ctrl, n = 9 slices from 3 mice,<br>cKO, n = 13 slices from 5 mice,<br>independent control pathway recordings, n = 6 slices from 3 mice	F	Assumed	One-way ANOVA	F (2, 25) = 19.96	P<0.0001	Tukey's multiple comparisons test:<br>Ctrl > cKO, p=0.0032<br>Ctrl > Control Pathway, p<0.0001<br>cKO> Control Pathway, p=0.0051

Statistic: Figure 5

Figure	Data ID	Behaviour Test	Sample Size	Sex	Normally distributed (Shapiro-Wilk test)?	Test	Factor	F, df	p value	Post-hoc test
Fig.5A	distance travelled M F	OF	Ctrl - M, n=18<br>Ctrl - F, n = 16<br>cKO - M, n =12<br>cKO - F, n = 16	M/F	Assumed	Two-way ANOVA	Interaction	F (1, 58) = 0.2718	0.6041	
							sex	F (1, 58) = 0.2837	0.5963	
							gen	F (1, 58) = 0.8710	0.3546	
Fig.5B	time in center (s) M F	OF	Ctrl - M, n=18<br>Ctrl - F, n = 16<br>cKO - M, n =12<br>cKO - F, n = 16	M/F	Assumed	Two-way ANOVA	Interaction	F (1, 58) = 0.8602	0.3575	
							sex	F (1, 58) = 0.2704	0.605	
							gen	F (1, 58) = 0.04087	0.8405	
Fig.5C	time RIM (s) M F	OF	Ctrl - M, n=18<br>Ctrl - F, n = 16<br>cKO - M, n =12<br>cKO - F, n = 16	M/F	Assumed	Two-way ANOVA	Interaction	F (1, 58) = 1.267	0.2649	
							sex	F (1, 58) = 4.707	**0.0342**	
							gen	F (1, 58) = 0.03028	0.8625	
Fig.5D	Object preference (L vs. R)	NORT	Ctrl - M, n=34<br>cKO - M, n =28	M/F	Assumed	Two-way ANOVA	Interaction	F (1, 116) = 0.4296	0.5135	
							Genotype	F (1, 116) = 0.000	0.9999	
							Object	F (1, 116) = 0.7413	0.391	
Fig.5E	% novel object recognition M F	NORT	Ctrl - M, n=13<br>Ctrl - F, n = 12<br>cKO - M, n =10<br>cKO - F, n = 13	M/F	Assumed	Two-way ANOVA	Interaction	F (1, 44) = 0.5164	0.4762	
							sex	F (1, 44) = 2.895	0.0959	
							Genotype	F (1, 44) = 10.02	**0.0028**	
Fig.5F	% sociability M F	Social Interaction	Ctrl - M, n= 18<br>Ctrl - F, n = 16<br>cKO - M, n =12<br>cKO - F, n = 16	M/F	Assumed	Two-way ANOVA	Interaction	F (1, 57) = 0.5986	0.4423	
							Sex	F (1, 57) = 1.479	0.229	
							Genotype	F (1, 57) = 0.001723	0.967	
Fig.5G	% Light M F	LD	Ctrl - M, n= 18<br>Ctrl - F, n = 16	M/F	Assumed	Two-way ANOVA	Interaction	F (1, 58) = 0.7191	0.3999	

			cKO - M, n =12<br>cKO - F, n = 16				Sex	F (1, 58) = 2.522	0.1177	
							Genotype	F (1, 58) = 0.2342	0.6302	
							Interaction	F (1, 58) = 0.7540	0.3888	
Fig.5H	Latency to dark M F	LD	Ctrl - M, n= 18<br>Ctrl - F, n = 16<br>cKO - M, n =12<br>cKO - F, n = 16	M/F	Assumed	Two-way ANOVA	Sex	F (1, 58) = 1.952	0.1677	
							Genotype	F (1, 58) = 0.09217	0.7625	
							Interaction	F (1, 58) = 0.1837	0.6698	
Fig.5I	Light transitions M F	LD	Ctrl - M, n= 18<br>Ctrl - F, n = 16<br>cKO - M, n =12<br>cKO - F, n = 16	M/F	Assumed	Two-way ANOVA	Sex	F (1, 58) = 1.055	0.3087	
							Genotype	F (1, 58) = 0.4665	0.4973	
Fig.5J	TWA	TWA	Ctrl, n = 27<br>cKO, n = 27	M/F	Assumed	Two-way RM ANOVA	days x genotype	F (4, 208) = 2.407	P=0.0506	
							days	F (4, 208) = 38.80	P<0.0001	
							genotype	F (1, 52) = 3.037	P=0.0873	
Fig.5K	TWA male	TWA	Ctrl - M, n = 17<br>cKO - M, n = 12	M	Assumed	Two-way RM ANOVA	days x genotype	F (4, 108) = 2.664	**0.0363**	Šídák's multiple comparisons test (ns)
							days	F (4, 108) = 17.06	**<0.0001**	
							genotype	F (1, 27) = 0.3981	0.5334	
Fig.5L	TWA female ELS19+20	TWA	Ctrl - F, n = 16<br>cKO - F, n = 15	F	Assumed	Two-way RM ANOVA	days x genotype	F (4, 116) = 3.010	**0.021**	Šídák's multiple comparisons test day 2: p=0.0339
							days	F (4, 116) = 34.66	**<0.0001**	
							genotype	F (1, 29) = 3.529	0.0704	

Statistic: Supplementary Figure 2

Figure	Data ID	Method	Region	Sample Size	Sex	Normally distributed (Shapiro-Wilk test)?	Test	U, t or F values	p value
Fig. S2B	Cortex % GR+NG2+OLIG2/NG2+OLIG	Histology	Cortex	Ctrl, n=4<br>cKO, n=5	M/F	YES	Unpaied t test (2-tailed)	t=4.956, df=7	**0.0016**
Fig. S2C	Cortex NG2+OLIG2+ cells mm2	Histology	Cortex	Ctrl, n=4<br>cKO, n=5	M/F	YES	Unpaied t test (2-tailed)	t=2.718, df=7	**0.0299**
Fig. S2D	Cortex ASPA+ cells mm2	Histology	Cortex	Ctrl, n=4<br>cKO, n=3	M/F	YES	Unpaired t test with Welch's correction (2-t	t=1.036, df=2.114	0.4041
Fig. S2E	Cortex PDGFRalpha/Ki67/DAPI	Histology	Cortex	Ctrl, n=10<br>cKO, n=16	M/F	YES	Unpaied t test (2-tailed)	t=1.123, df=24	0.2725
Fig. S2F	Hippo PDGFRalpha/DAPI cells mm2	Histology	Hippocampus	Ctrl, n=10<br>cKO, n=16	M/F	YES	Unpaied t test (2-tailed)	t=0.4133, df=24	0.683
Fig. S2G	Cortex PDGFRalpha/DAPI cells mm2	Histology	Cortex	Ctrl, n=10<br>cKO, n=16	M/F	YES	Unpaied t test (2-tailed)	t=1.441, df=24	0.1626
Fig. S2H	% PDGFRA+ Cx	FACS	Cortex	Ctrl, n=8<br>cKO, n=7*	M/F	YES	Unpaied t test (2-tailed)	t=0.5312, df=13	0.6042
Fig. S2I	% CNPase+ Cx	FACS	Cortex	Ctrl, n=8<br>cKO, n=7*	M/F	YES	Unpaied t test (2-tailed)	t=0.3893, df=13	0.7033

Statistic: Supplementary Figure 3

Figure	Sample ID	Method	Region	Sample Size	Sex	Normally distributed (Shapiro-Wilk test)?	Test	Factor	F, df	p value
Fig. S3A	%OPC - GR - Hippo	Histology	Hippocampus	Ctrl - M, n=7<br>Ctrl - F, n = 7<br>cKO - M, n =8<br>cKO - F, n = 7	M/F	Assumed	Two-way ANOVA	Interaction	F (1, 25) = 0.0002001	P=0.9888
								sex	F (1, 25) = 0.008369	P=0.9278
								genotype	F (1, 25) = 31.75	P<0.0001
Fig. S3B	%OPC - GR - Cortex	Histology	Cortex	Ctrl - M, n=7<br>Ctrl - F, n = 6<br>cKO - M, n =8<br>cKO - F, n = 8	M/F	Assumed	Two-way ANOVA	Interaction	F (1, 25) = 0.2711	P=0.6072
								sex	F (1, 25) = 0.5317	P=0.4727
								genotype	F (1, 25) = 24.58	P<0.0001

Statistic: Supplementary Figure 4

Figure	Data ID	Method	Region	Sample Size	Sex	Normally distributed (Shapiro-Wilk test)?	Test	U, t or F values	p value
Fig. S4K	% GR+ Pericyte in CA1	Histology	Hippocampus	Ctrl, n=15<br>cKO, n=16	M/F	No	Mann-Whitney Test	U=95.50	0.3407
Fig.S4L	% GR+ Pericyte in Cortex	Histology	Cortex	Ctrl, n=15<br>cKO, n=16	M/F	Yes	Unpaired t test with Welch's correction	t=1.454, df=21.10	0.1606

Statistic: Supplementary Figure 5

Figure	Data ID	Method	Region	Sample Size	Sex	Test	U, t or F values	p value
Fig.S5D	Filament length sum	Histology	Hippocampus	Ctrl (n=34 cells| n=3mice)<br>cKO (n=22 cells | n=2 mice)	F	F test to compare variances	F, DFn, Dfd = 1.056, 21, 33	0.867
Fig.S5E	Intersection sum	Histology	Hippocampus	Ctrl (n=34 cells| n=3mice)<br>cKO (n=22 cells | n=2 mice)	F	F test to compare variances	F, DFn, Dfd = 1.004, 21, 33	0.9676

Statistic: Supplementary Figure 6

Figure	Data ID	Region	Sample Size	Sex	Test	U, t or F values	p value
Fig.S6A	MBP	NA	Protein dose, n=5	NA	Simple linear regression	F (1, 8) = 20.53	**0.0019**
Fig.S6B	*aspa rabbit	NA	Protein dose, n=5	NA	Simple linear regression	F (1, 8) = 76.44	**<0.0001**
Fig.S6C	*ng2 rabbit v3 10-30ug 95	NA	Protein dose, n=5	NA	Simple linear regression	F (1, 8) = 32.84	**<0.0001**
Fig.S6D	*cnp ms	NA	Protein dose, n=5	NA	Simple linear regression	F (1, 8) = 10.07	**0.0131**
Fig.S6E	*plp rat 1-10ug v2	NA	Protein dose, n=5	NA	Simple linear regression	F (1, 8) = 3451	**<0.0001**

Statistic: Supplementary Figure 7

Figure	Data ID	Region	Sample Size	Sex	Normally distributed (Shapiro-Wilk test)?	Test	Factor	U, t or F values	p value
Fig.S7B	NG2 male vs fem	Hippocampus	Ctrl - M, n=4<br>cKO - M, n=4<br>Ctrl - F, n=3<br>cKO - F, n=3	M/F	Assumed	Two-way ANOVA	Interaction	F (1, 10) = 0.5008	0.4953
							Sex	F (1, 10) = 0.5008	0.4953
							Genotype	F (1, 10) = 19.76	**0.0012**
Fig.S7C	NG2 male vs fem	Cortex	Ctrl - M, n=4<br>cKO - M, n=4<br>Ctrl - F, n=3<br>cKO - F, n=3	M/F	Assumed	Two-way ANOVA	Interaction	F (1, 10) = 0.2729	0.6128
							Sex	F (1, 10) = 0.2729	0.6128
							Genotype	F (1, 10) = 5.060	**0.0482**
Fig.S7E	MBP male vs fem	Hippocampus	Ctrl - M, n=4<br>cKO - M, n=4<br>Ctrl - F, n=3<br>cKO - F, n=3	M/F	Assumed	Two-way ANOVA	Interaction	F (1, 10) = 0.2471	0.6299
							Sex	F (1, 10) = 0.2471	0.6299
							Genotype	F (1, 10) = 0.02837	0.8696
Fig.S7F	MBP male vs fem	Cortex	Ctrl - M, n=4<br>cKO - M, n=4<br>Ctrl - F, n=3<br>cKO - F, n=3	M/F	Assumed	Two-way ANOVA	Interaction	F (1, 10) = 0.07756	0.7863
							Sex	F (1, 10) = 0.09330	0.7663
							Genotype	F (1, 10) = 1.650	0.2279
Fig.S7H	PLP male vs fem	Hippocampus	Ctrl - M, n=4<br>cKO - M, n=4<br>Ctrl - F, n=3<br>cKO - F, n=3	M/F	Assumed	Two-way ANOVA	Interaction	F (1, 10) = 0.2573	0.623
							Sex	F (1, 10) = 0.2573	0.623
							Genotype	F (1, 10) = 0.1087	0.7484
Fig.S7I	PLP male vs fem	Cortex	Ctrl - M, n=4<br>cKO - M, n=4<br>Ctrl - F, n=3<br>cKO - F, n=3	M/F	Assumed	Two-way ANOVA	Interaction	F (1, 10) = 0.1371	0.7189
							Sex	F (1, 10) = 0.1260	0.73
							Genotype	F (1, 10) = 0.5673	0.4687
Fig.S7K	ASPA male vs fem	Hippocampus	Ctrl - M, n=4<br>cKO - M, n=4<br>Ctrl - F, n=3<br>cKO - F, n=3	M/F	Assumed	Two-way ANOVA	Interaction	F (1, 10) = 0.3819	0.5504
							Sex	F (1, 10) = 0.3819	0.5504
							Genotype	F (1, 10) = 2.253	0.1643
Fig.S7L	ASPA male vs fem	Cortex	Ctrl - M, n=4<br>cKO - M, n=4<br>Ctrl - F, n=3<br>cKO - F, n=3	M/F	Assumed	Two-way ANOVA	Interaction	F (1, 10) = 0.1039	0.7538
							Sex	F (1, 10) = 0.1039	0.7538
							Genotype	F (1, 10) = 1.532	0.2442
Fig.S7N	CNP male vs fem	Hippocampus	Ctrl - M, n=4<br>cKO - M, n=4<br>Ctrl - F, n=3<br>cKO - F, n=3	M/F	Assumed	Two-way ANOVA	Interaction	F (1, 10) = 0.1378	0.7182
							Sex	F (1, 10) = 0.1597	0.6978
							Genotype	F (1, 10) = 0.3203	0.5839
Fig.S7O	CNP male vs fem	Cortex	Ctrl - M, n=4<br>cKO - M, n=4<br>Ctrl - F, n=3<br>cKO - F, n=3	M/F	Assumed	Two-way ANOVA	Interaction	F (1, 10) = 0.3048	0.593
							Sex	F (1, 10) = 0.3204	0.5839
							Genotype	F (1, 10) = 1.292	0.2822

Statistic: Supplementary Figure 8

Model	Term	Estimate = Beta	StdError	tValue	pValue	Significance
model_N_G	(Intercept)	0.79847214	0.01752242	45.5685978	5.0354E-26	***
	factor(Genotype)transgenic	0.00254495	0.02414585	0.10539899	0.91686085	ns
model_N_G_lm	(Intercept)	0.79852888	0.01351105	59.1019309	0	***
	factor(Genotype)transgenic	0.0010953	0.01868479	0.05861988	0.95326142	ns
model_N_S	(Intercept)	0.7913921	0.01700683	46.5337745	3.3422E-27	***
	factor(Sex)female	0.01647036	0.02382503	0.69130505	0.49549129	ns
model_N_G_S	(Intercept)	0.78950212	0.02202782	35.8411382	6.5914E-23	***
	factor(Genotype)transgenic	0.00357759	0.02437475	0.14677438	0.88449005	ns
	factor(Sex)female	0.01657946	0.02434406	0.68104753	0.50209143	ns
model_N_G_S_i	(Intercept)	0.80119904	0.02619938	30.5808439	1.4975E-20	***
	factor(Genotype)transgenic	-0.01736	0.03509228	-0.494697	0.62521904	ns
	factor(Sex)female	-0.0050464	0.03562324	-0.1416614	0.88856072	ns
	factor(Genotype)transgenic:factor(Sex)female	0.04090199	0.04902038	0.83438747	0.41232093	ns
model_Pn_G	(Intercept)	1.79257954	0.03823403	46.8844027	2.4467E-24	***
	factor(Genotype)transgenic	0.03827054	0.05227194	0.73214317	0.47146662	ns
model_Pn_G_nd	(Intercept)	1.78545258	0.03790711	47.1007304	2.64E-24	***
	factor(Genotype)transgenic	0.04374584	0.05184502	0.84378104	0.40749193	ns
model_Pn_S	(Intercept)	1.80086779	0.0371433	48.4843287	9.7509E-25	***
	factor(Sex)female	0.02437545	0.05243609	0.46486016	0.64640666	ns
model_Pn_S_nd	(Intercept)	1.79623901	0.03694344	48.6213227	1.0274E-24	***
	factor(Sex)female	0.02523769	0.05217534	0.48370908	0.63317789	ns
model_Pn_G_S	(Intercept)	1.77790235	0.04836658	36.7589039	1.6949E-21	***
	factor(Genotype)transgenic	0.04034884	0.05336491	0.75609305	0.45748135	ns
	factor(Sex)female	0.02728032	0.05323416	0.51245887	0.61335121	ns
model_Pn_G_S_i	(Intercept)	1.78992755	0.05857251	30.5591734	2.5683E-19	***
	factor(Genotype)transgenic	0.0193243	0.07759717	0.24903361	0.8056677	ns
	factor(Sex)female	0.0050244	0.07978821	0.06297168	0.95036598	ns
	factor(Genotype)transgenic:factor(Sex)female	0.04192932	0.10902212	0.3845946	0.7042814	ns
model_Pn_G_S_i_nd	(Intercept)	1.78075221	0.05809127	30.6543865	2.6327E-19	***
	factor(Genotype)transgenic	0.02724725	0.07694172	0.35412844	0.72665894	ns
	factor(Sex)female	0.00877776	0.07911075	0.11095534	0.91267678	ns
	factor(Genotype)transgenic:factor(Sex)female	0.03734728	0.10813849	0.34536525	0.73315372	ns
model_TL_G	(Intercept)	4.24669677	0.06064947	70.020344	7.4655E-21	***
	factor(Genotype)transgenic	0.06755048	0.08350823	0.80890812	0.43049382	ns

model_TL_S	(Intercept)	4.25461803	0.05769068	73.7487902	5.7536E-20	***
	factor(Sex)female	0.0547227	0.0818367	0.66868164	0.51403218	ns
model_TL_G_S	(Intercept)	4.21165542	0.07786521	54.0890543	4.5732E-19	***
	factor(Genotype)transgenic	0.07586947	0.08606624	0.88152423	0.3912999	ns
	factor(Sex)female	0.06296397	0.08593421	0.73269972	0.47448323	ns
model_TL_G_S_i	(Intercept)	4.17410501	0.09250077	45.1250835	1.0463E-16	***
	factor(Genotype)transgenic	0.14163191	0.1223791	1.15732104	0.26618522	ns
	factor(Sex)female	0.13186817	0.12544332	1.05121712	0.31076893	ns
	factor(Genotype)transgenic:factor(Sex)female	-0.1303117	0.17266497	-0.7547083	0.46247285	ns

Figure	**Data ID**	**Method**	**Region**	**Sample Size**	**Sex**	**Normally distributed (Shapiro-Wilk test)?**	**Test**	Factors	**U, t or F values**	**p value**
Fig. S8G	Myelin content in CA1	Histology	Hippocampus	Ctrl - M, n = 4 cKo - M, n = 5 Ctrl - F, n = 5 cKO - F, n = 6	M/F	Assumed	Two-way ANOVA	Interaction	F (1, 16) = 0.5999	P=0.4499
								sex	F (1, 16) = 0.01383	P=0.9078
								genotype	F (1, 16) = 0.09833	P=0.7579
Fig. S8H	Internode length	Histology	Hippocampus	Ctrl - M, n = 4 cKo - M, n = 5 Ctrl - F, n = 5 cKO - F, n = 6	M/F	Assumed	Two-way ANOVA	Interaction	F (1, 16) = 0.2305	P=0.6377
								sex	F (1, 16) = 0.04783	P=0.8296
								genotype	F (1, 16) = 0.5837	P=0.4560
Fig. S8I	Frequency of internode with defined length	Histology	Hippocampus	Ctrl - M, n = 4 cKo - M, n = 5 Ctrl - F, n = 5 cKO - F, n = 6	M/F	Assumed	Three-way ANOVA	Internode range	F (7, 112) = 19.58	P<0.0001
								sex	F (1, 16) = 2.898	P=0.1080
								genotype	F (1, 16) = 1.619	P=0.2214
								Internode range x sex	F (7, 112) = 0.6832	P=0.6860
								Internode range x genotype	F (7, 112) = 0.2093	P=0.9827
								sex x genotype	F (1, 16) = 0.06740	P=0.7985
								Internode range x sex x genotype	F (7, 112) = 2.086	P=0.0507

Statistic: Supplementary figure 10

Figure	Data ID	Method	Region	Sample Size	Sex	Normally distributed (Shapiro-Wilk test)?	Test	Factor	F, df	p value	Post-hoc test
Fig. S10A	fEPSP	MEA recording	Hippocampus	Ctrl F, n = 16<br>Ctrl M, n = 18	M/F	Assumed	Two-way RM ANOVA	Input x Sex	F (9, 288) = 7.622	<0.0001	Sidak's Multiple comparison<br>M>F<br>Input 3, p = 0.0185<br>Input 3.5, p = 0.0043<br>input 4, p = 0.0012<br>Input 4.5, p = 0.0004<br>Input 5, p = 0.0002
								Input	F (9, 288) = 84.41	<0.0001	
								Sex	F (1, 32) = 8.700	0.0059	
Fig. S10B	fEPSP	MEA recording	Hippocampus	cKO F, n = 17<br>cKO M, n = 22	M/F	Assumed	Two-way RM ANOVA	Input x Sex	F (9, 333) = 9.396	<0.0001	Sidak's Multiple comparison<br>M>F<br>Input 3.5, p = 0.0412<br>input 4, p = 0.0039<br>Input 4.5, p = 0.0006<br>Input 5, p < 0.0001
								Input	F (9, 333) = 91.40	<0.0001	
								Sex	F (1, 37) = 5.307	0.027	
Fig. S10C	2wayANOVA of male, female, ctrl, cko (-DEX)	MEA recording	Hippocampus	Ctrl M, n=18<br>Ctrl F, n =19<br>cKO M, n =23<br>cKO F, n = 17*	M/F	Assumed	Two-way ANOVA	Interaction	F (1, 73) = 0.8740	0.3529	
								Sex	F (1, 73) = 0.4589	0.5003	
								Genotype	F (1, 73) = 3.934	0.0511	
Fig.S10D	fem WT before vs after Dex	MEA recording	Hippocampus	WT (-Dex), n = 16*<br>WT (+Dex), n=16*	F	NO	Wilcoxon matched-pairs signed rank test		W = -60.00	0.1297	
Fig.S10D	fem KO before vs after Dex	MEA recording	Hippocampus	cKO (-Dex), n = 15*<br>cKO (+Dex), n=15*	F	NO	Wilcoxon matched-pairs signed rank test		W = -46.00	0.2078	
Fig.S10D	male WT before vs after Dex	MEA recording	Hippocampus	WT (-Dex), n = 17<br>WT (+Dex), n=17	M	YES	Paired t test		t=1.044, df=16	0.3121	
Fig.S10D	male KO before vs after Dex	MEA recording	Hippocampus	cKO (-Dex), n = 20*<br>cKO (+Dex), n=20*	M	NO	Wilcoxon matched-pairs signed rank test		W = 29	0.6025	
Fig.S10E	2wayANOVA male, female, ctrl, cko (-DEX)	MEA recording	Hippocampus	Ctrl M (-Dex), n = 18<br>Ctrl f (-Dex), n = 15*<br>cKO M (-Dex) , n= 20*<br>cKO F (-Dex), n = 15*	M/F	Assumed	Two-way ANOVA	Interaction	F (1, 64) = 0.7046	0.4044	
								sex	F (1, 64) = 10.07	**0.0023**	
								genotype	F (1, 64) = 3.983	0.0502	

Statistic: Supplementary figure 12

Figure	Data ID	Sample Size	Sex	Normally distributed (Shapiro-Wilk test)?	Test	Factor	F, df	p value
Fig.S12B	dd testing NORT	Ctrl - M, n=18<br>Ctrl - F, n = 16<br>cKO - M, n =12<br>cKO - F, n = 16	M/F	Assumed	Three-way RM ANOVA	time	F (2.517, 146.0) = 3.113	P=0.0362
						(M vs F)	F (1, 58) = 0.000	P>0.9999
						(Ctrl vs cKO)	F (1, 58) = 0.000	P>0.9999
						time x (M vs F)	F (9, 522) = 4.064	**P<0.0001**
						time x (Ctrl vs cKO)	F (9, 522) = 1.526	P=0.1355
						(M vs F) x (Ctrl vs cKO)	F (1, 58) = 0.000	P>0.9999
						time x (M vs F) x (Ctrl vs cKO)	F (9, 522) = 0.9740	P=0.4606
Fig.S12C	da testing NORT	Ctrl - M, n=18<br>Ctrl - F, n = 16<br>cKO - M, n =12<br>cKO - F, n = 16	M/F	Assumed	Three-way RM ANOVA	time	F (3.355, 194.6) = 1.916	P=0.1211
						(M vs F)	F (1, 58) = 0.000	P>0.9999
						(Ctrl vs cKO)	F (1, 58) = 0.000	P>0.9999
						time x (M vs F)	F (9, 522) = 2.269	**P=0.0169**
						time x (Ctrl vs cKO)	F (9, 522) = 1.121	P=0.3456
						(M vs F) x (Ctrl vs cKO)	F (1, 58) = 0.000	P>0.9999
						time x (M vs F) x (Ctrl vs cKO)	F (9, 522) = 0.6864	P=0.7215

Statistic: Supplementary figure 13

Figure	Data ID	Behaviour Test	Sample Size	Sex	Normally distributed (Shapiro-Wilk test)?	Test	Factor	F, df	p value	Post-hoc test
Fig. S13	TWA F M	TWA	Ctrl - M, n=17* Ctrl - F, n = 16 cKO - M, n =12 cKO - F, n = 15*	M/F	Assumed	Three-way ANOVA	day	F (2.599, 145.5) = 48.75	**<0.0001**	
							sex	F (1, 56) = 0.5207	0.4736	
							genotype	F (1, 56) = 3.092	0.0842	
							day x sex	F (4, 224) = 0.5710	0.684	
							day x genotype	F (4, 224) = 3.118	**0.016**	
							sex x genotype	F (1, 56) = 0.7254	0.398	
							day x sex x genotype	F (4, 224) = 2.555	**0.0398**	Tested below with 2-way ANOVA per day
Fig. S13A	TWA day 1	TWA	Ctrl - M, n=17 cKO - M, n =12	M	Assumed?	Mixed-effects model (REML)	trial	F (1.349, 36.38) = 35.06	**<0.0001**	
							genotype	F (1, 27) = 2.169	0.1524	
							trial x genotype	F (24, 647) = 2.029	**0.0027**	Šídák's multiple comparisons test (ns)
Fig. S13B	TWA day 2	TWA	Ctrl - M, n=17 cKO - M, n =12	M	Assumed	Two-way RM ANOVA	trial x genotype	F (24, 648) = 4.590	**<0.0001**	Šídák's multiple comparisons test (ns)
							trial	F (1.248, 33.70) = 45.88	**<0.0001**	
							genotype	F (1, 27) = 4.248	**0.0491**	
Fig. S13C	TWA day 3	TWA	Ctrl - M, n=17 cKO - M, n =12	M	Assumed	Two-way RM ANOVA	trial x genotype	F (24, 648) = 0.9818	0.4884	
							trial	F (1.205, 32.54) = 59.25	**<0.0001**	
							genotype	F (1, 27) = 2.486	0.1265	
Fig. S13D	TWA day 4	TWA	Ctrl - M, n=17 cKO - M, n =12	M	Assumed	Two-way RM ANOVA	trial x genotype	F (24, 648) = 0.1459	>0.9999	
							trial	F (1.108, 29.92) = 60.78	**<0.0001**	
							genotype	F (1, 27) = 0.07047	0.7927	
Fig. S13E	TWA day 5	TWA	Ctrl - M, n=17 cKO - M, n =12	M	Assumed	Two-way RM ANOVA	trial x genotype	F (24, 648) = 0.1261	>0.9999	
							trial	F (24, 648) = 54.07	**<0.0001**	

							genotype	F (1, 27) = 0.1101	0.7426	
Fig. S13F	TWA day 1	TWA	Ctrl - F, n = 16<br>cKO - F, n = 15	F	Assumed	Two-way RM ANOVA	trial x genotype	F (24, 696) = 0.1068	>0.9999	
							trial	F (24, 696) = 21.03	**<0.0001**	
							genotype	F (1, 29) = 0.06781	0.7964	
Fig. S13G	TWA day 2	TWA	Ctrl - F, n = 16<br>cKO - F, n = 15	F	Assumed	Two-way RM ANOVA	trial x genotype	F (24, 696) = 9.818	**<0.0001**	Šídák's multiple comparisons test (ns)
							trial	F (1.200, 34.79) = 69.09	**<0.0001**	
							genotype	F (1, 29) = 8.782	**0.006**	
Fig. S13H	TWA day 3	TWA	Ctrl - F, n = 16<br>cKO - F, n = 15	F	Assumed	Two-way RM ANOVA	trial x genotype	F (24, 696) = 3.985	**<0.0001**	Šídák's multiple comparisons test (ns)
							trial	F (1.111, 32.22) = 74.06	**<0.0001**	
							genotype	F (1, 29) = 3.427	0.0743	
Fig. S13I	TWA day 4	TWA	Ctrl - F, n = 16<br>cKO - F, n = 15	F	Assumed	Two-way RM ANOVA	trial x genotype	F (24, 696) = 2.516	**<0.0001**	Šídák's multiple comparisons test (ns)
							trial	F (1.176, 34.10) = 96.89	**<0.0001**	
							genotype	F (1, 29) = 2.879	0.1004	
Fig. S13J	TWA day 5	TWA	Ctrl - F, n = 16<br>cKO - F, n = 15	F	Assumed	Two-way RM ANOVA	trial x genotype	F (24, 696) = 1.460	0.0727	
							trial	F (1.096, 31.79) = 90.33	**<0.0001**	
							genotype	F (1, 29) = 1.329	0.2584	

Statistic: Supplementary figure 14

Figure	Behaviour Test	Sample Size	Sex	Normally distributed (Shapiro-Wilk test)?	Test	Factor	F, df	p value	Post-hoc test
Fig. S14A	OFT - distance travelled	Ctrl - M, n=9<br>cKO - M, n =9<br>Ctrl - F, n=6<br>cKO - F, n =11	M/F	Assumed	Two-way ANOVA	Interaction	F (1, 31) = 0.03289	P=0.8573	
						sex	F (1, 31) = 19.53	**P=0.0001**	
						genotype	F (1, 31) = 0.07048	P=0.7924	
Fig. S14B	OFT - Time Center	Ctrl - M, n=9<br>cKO - M, n =9<br>Ctrl - F, n=6<br>cKO - F, n =11	M/F	Assumed	Two-way ANOVA	Interaction	F (1, 31) = 0.2312	P=0.6340	
						sex	F (1, 31) = 3.687	P=0.0641	
						genotype	F (1, 31) = 0.4649	P=0.5004	
Fig. S14C	OFT - Time RIM	Ctrl - M, n=9<br>cKO - M, n =9<br>Ctrl - F, n=6<br>cKO - F, n =11	M/F	Assumed	Two-way ANOVA	Interaction	F (1, 31) = 0.1065	P=0.7464	
						sex	F (1, 31) = 0.5076	P=0.4815	
						genotype	F (1, 31) = 4.474e-007	P=0.9995	
Fig. S14D	LDBT - distance travelled	Ctrl - M, n=9<br>cKO - M, n =9<br>Ctrl - F, n=6<br>cKO - F, n =11	M/F	Assumed	Two-way ANOVA	Interaction	F (1, 31) = 0.09117	P=0.7647	
						sex	F (1, 31) = 9.151	**P=0.0050**	
						genotype	F (1, 31) = 2.890	P=0.0991	
Fig. S14E	LDBT - time in light (%)	Ctrl - M, n=9<br>cKO - M, n =9<br>Ctrl - F, n=6<br>cKO - F, n =11	M/F	Assumed	Two-way ANOVA	Interaction	F (1, 32) = 0.2490	P=0.6212	
						sex	F (1, 32) = 3.895	P=0.0571	
						genotype	F (1, 32) = 0.04077	P=0.8413	
Fig. S14F	LDBT - Latency to dark	Ctrl - M, n=9<br>cKO - M, n =9<br>Ctrl - F, n=6<br>cKO - F, n =11	M/F	Assumed	Two-way ANOVA	Interaction	F (1, 31) = 1.438	P=0.2395	
						sex	F (1, 31) = 0.1600	P=0.6919	
						genotype	F (1, 31) = 1.065	P=0.3100	
Fig. S14G	Social Interaction	Ctrl - M, n=9<br>cKO - M, n =9<br>Ctrl - F, n=6<br>cKO - F, n =11	M/F	Assumed	Two-way ANOVA	Interaction	F (1, 30) = 3.773	P=0.0615	
						sex	F (1, 30) = 0.3074	P=0.5834	
						genotype	F (1, 30) = 1.158	P=0.2905	
Fig. S14H	Novel object recognition	Ctrl - M, n=9<br>cKO - M, n =9<br>Ctrl - F, n=6<br>cKO - F, n =11	M/F	Assumed	Two-way ANOVA	Interaction	F (1, 31) = 1.807	P=0.1886	
						sex	F (1, 31) = 0.01255	P=0.9115	
						genotype	F (1, 31) = 0.02386	P=0.8782	
Fig. S14I	TWA	Ctrl - M, n=9<br>cKO - M, n =9	M	Assumed	Two-way RM ANOVA	Time x genotype	F (2.005, 32.08) = 0.5761	P=0.5682	
						Time	F (2.005, 32.08) = 3.425	**P=0.0447**	
						genotype	F (1, 16) = 0.05503	P=0.8175	
Fig. S14J	TWA	Ctrl - F, n=6<br>cKO - F, n =11	F	Assumed	Two-way RM ANOVA	Time x genotype	F (1.980, 35.63) = 1.824	P=0.1765	
						Time	F (1.980, 35.63) = 19.79	**P<0.0001**	

						genotype	F (1, 18) = 2.330	P=0.1443
Fig. S14K	TWA	Ctrl - M, n=9<br>cKO - M, n =9<br>Ctrl - F, n=6<br>cKO - F, n =11	M/F	Assumed	Three-way ANOVA	time	F (2.101, 71.44) = 18.65	**P<0.0001**
						sex	F (1, 34) = 1.821	P=0.1861
						genotype	F (1, 34) = 0.7066	P=0.4065
						time x sex	F (2.101, 71.44) = 3.918	**P=0.0226**
						time x genotype	F (2.101, 71.44) = 1.238	P=0.2971
						sex x genotype	F (1, 34) = 1.423	P=0.2411
						time x sex x genotype	F (2.101, 71.44) = 1.112	P=0.3367

Statistic: Supplementary Figure 15

Figure	Data ID	Method	Region	Sample Size	Sex	Normally distributed (Shapiro-Wilk test)?	Test	Factors	U, t or F values	p value
Fig S15A	OPC Hippocampus	Histology	Hippocampus	Ctrl - M, n=4<br>cKO M, n=4<br>Ctrl - F, n = 4<br>cKO - F, n =4	M/F	Assumed	Two-way ANOVA	Interaction	F (1, 12) = 0.1381	P=0.7167
								Sex	F (1, 12) = 0.1837	P=0.6758
								Genotype	F (1, 12) = 0.03999	P=0.8448
Fig S15B	OPC Cortex	Histology	Cortex	Ctrl - M, n=4<br>cKO M, n=4<br>Ctrl - F, n = 4<br>cKO - F, n =4	M/F	Assumed	Two-way ANOVA	Interaction	F (1, 12) = 2.239	P=0.1604
								Sex	F (1, 12) = 0.1854	P=0.6744
								Genotype	F (1, 12) = 0.01109	P=0.9179
Fig S15C	%GR+ OPC/OPC Hippocampus	Histology	Hippocampus	Ctrl - M, n=4<br>cKO M, n=4<br>Ctrl - F, n = 4<br>cKO - F, n =4	M/F	Assumed	Two-way ANOVA	Interaction	F (1, 12) = 0.0009083	P=0.9765
								Sex	F (1, 12) = 1.195	P=0.2958
								Genotype	F (1, 12) = 11.89	P=0.0048
Fig S15D	%GR+ OPC/OPC Cortex	Histology	Cortex	Ctrl - M, n=4<br>cKO M, n=4<br>Ctrl - F, n = 4<br>cKO - F, n =4	M/F	Assumed	Two-way ANOVA	Interaction	F (1, 12) = 0.08102	P=0.7808
								Sex	F (1, 12) = 0.8020	P=0.3881
								Genotype	F (1, 12) = 5.891	P=0.0319
Fig S15E	OL Hippocampus	Histology	Hippocampus	Ctrl - M, n=4<br>cKO M, n=4<br>Ctrl - F, n = 4<br>cKO - F, n =4	M/F	Assumed	Two-way ANOVA	Interaction	F (1, 12) = 1.410	P=0.2580
								Sex	F (1, 12) = 1.877	P=0.1958
								Genotype	F (1, 12) = 0.4844	P=0.4997
Fig S15F	OL Cortex	Histology	Cortex	Ctrl - M, n=4<br>cKO M, n=4<br>Ctrl - F, n = 4<br>cKO - F, n =4	M/F	Assumed	Two-way ANOVA	Interaction	F (1, 12) = 0.006014	P=0.9395
								Sex	F (1, 12) = 0.3486	P=0.5658
								Genotype	F (1, 12) = 0.01408	P=0.9075

Statistic: supplementary figure 16

Figure	Data ID	Behavioural test	Sample Size	Sex	Normally distributed (Shapiro-Wilk test)?	Test	Factor	U, t or F values	p value
Fig. S16A	OFT - WT vs. CreERT2	OFT	WT, n=10<br>CreERT2, n=10	M	YES	Unpaired t test with Welch's correction (2-tailed)		t = 1.247, df = 12.73	0.2347
Fig. S16B	OFT - WT vs. CreERT2	OFT	WT, n=10<br>CreERT2, n=10	M	NO	Mann-Whitney test (2-tailed)		U = 40.50	0.4935
Fig. S16C	OFT - WT vs. CreERT2	OFT	WT, n=10<br>CreERT2, n=10	M	YES	Unpaired t test (2-tailed)		t = 1.359, df = 18	0.1909
Fig. S16D	NORT - WT vs. CreERT2	NORT	WT, n=10<br>CreERT2, n=10	M	YES	Unpaired t test (2-tailed)<br>One-sample t test WT (50% chance level)<br>One-sample t test CreERT2 (50% chance level)		t = 0.7386, df = 18<br>t=0.6948, df=9<br>t=0.3484, df=9	0.4696<br>0.5047<br>0.7355
Fig. S16E	LDB - WT vs. CreERT2	LDBT	WT, n=9*<br>CreERT2, n=9*	M	NO	Mann-Whitney test (2-tailed)<br>Wilcoxon Signed Rank Test WT (50% chance level)<br>Wilcoxon Signed Rank test CreERT2 (50% chance level)		U=33<br>W =-45<br>W=-31	0.5457<br>0.0039<br>0.0742
Fig. S16F	LDB - WT vs. CreERT2	LDBT	WT, n=8*<br>CreERT2, n=8*	M	NO	Mann-Whitney test (2-tailed)		U=23	0.3667
Fig. S16G	LDB - WT vs. CreERT2	LDBT	WT, n=10<br>CreERT2, n=10	M	YES	Unpaired t test (2-tailed)		t=1.732, df=16	0.1024
Fig. S16H	TWA - WT vs. CreERT2	TWA	WT, n=10<br>CreERT2, n=10	M	Assumed	Two-way RM ANOVA	Day	F (2.827, 45.23) = 14.48	P<0.0001
							Genotype	F (1, 16) = 0.1835	P=0.6741
							Day x Genotype	F (2.827, 45.23) = 0.4450	P=0.7107
Fig. S16A'	OFT - WT vs. CreERT2	OFT	WT, n=11<br>CreERT2, n=9	F	YES	Unpaired t test (2-tailed)		t = 0.3743, df=18	0.7126
Fig. S16B'	OFT - WT vs. CreERT2	OFT	WT, n=11<br>CreERT2, n=9	F	YES	Unpaired t test (2-tailed)		t = 1.166, df=18	0.2589
Fig. S16C'	OFT - WT vs. CreERT2	OFT	WT, n=11<br>CreERT2, n=9	F	NO	Mann-Whitney test (2-tailed)		U = 40	0.5027
Fig. S16D'	NORT - WT vs. CreERT2	NORT	WT, n=11<br>CreERT2, n=9	F	YES	Unpaired t test (2-tailed)<br>One-sample t test WT (50% chance level)<br>One-sample t test CreERT2 (50% chance level)		t=0.7117, df=18<br>t=3.018, df=10<br>t=1.345, df=8	0.4858<br>0.0129<br>0.2156
Fig. S16C'	LDB - WT vs. CreERT2	LDBT	WT, n=11<br>CreERT2, n=9	F	YES	Unpaired t test (2-tailed)<br>One-sample t test WT (50% chance level)<br>One-sample t test CreERT2 (50% chance level)		t=0.1793, df=18<br>t=3.516, df=10<br>t=2.535, df=8	0.8597<br>0.0056<br>0.0350
Fig. S16F'	LDB - WT vs. CreERT2	LDBT	WT, n=11<br>CreERT2, n=9	F	NO	Mann-Whitney test (2-tailed)		U=32	0.2014
Fig. S16G'	LDB - WT vs. CreERT2	LDBT	WT, n=11<br>CreERT2, n=9	F	YES	Unpaired t test (2-tailed)		t=0.1459, df=18	0.8856
Fig. S16H'	TWA - WT vs. CreERT2	TWA	WT, n=10<br>CreERT2, n=10	M	Assumed	Two-way RM ANOVA	Day	F (2.828, 50.90) = 34.70	P<0.0001
							Genotype	F (1, 18) = 1.171	P=0.2934
							Day x Genotype	F (2.828, 50.90) = 1.354	P=0.2678
